# Supplementary material for: Insights into expression and localization of HPV16 LCR-associated transcription factors and association with LCR activity in HNSCC
Source: Mol Ther Oncol. 2024 Dec 21;33(1):200926. doi: 10.1016/j.omton.2024.200926 (PMC11780949; doi:10.1016/j.omton.2024.200926)
Supplement: Document S1. Figures S1–S7 and Tables S3–S7 [file mmc1.pdf]

OMTON, Volume *33*

Supplemental information

Insights into expression and localization of HPV16 LCR-associated transcription factors and association with LCR activity in HNSCC

Nikita Aggarwal, Divya Janjua, Apoorva Chaudhary, Udit Joshi, Tanya Tripathi, Chetkar Chandra Keshavam, Joni Yadav, Arun Chhokar, and Alok Chandra Bharti

Figure S1a

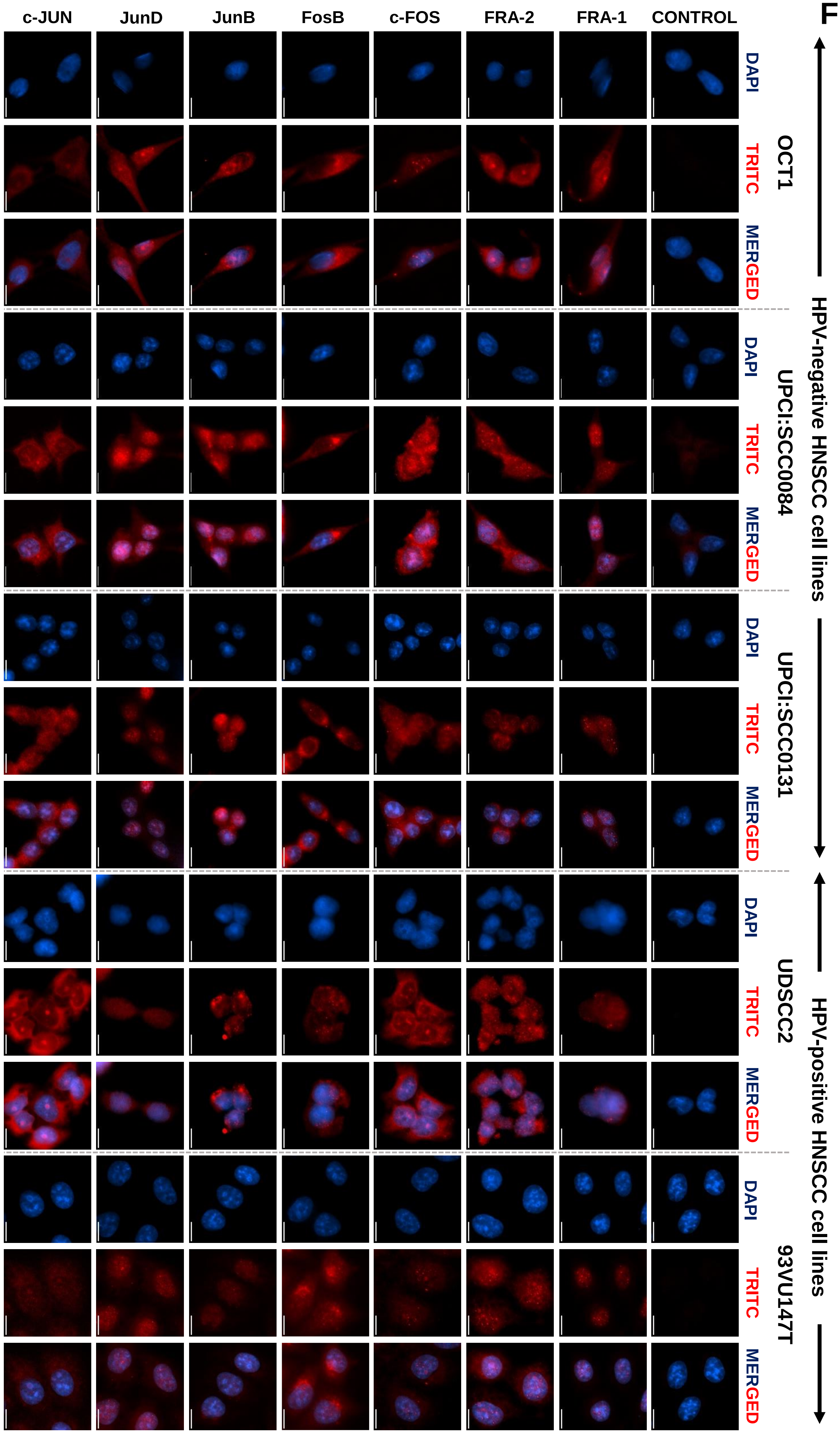

c-JUN
JunD
JunB
FosB
c-FOS
FRA-2
FRA-1
CONTROL
DAPI
TRITC
MERGED
OCT1
UPCI:SCC0084
UPCI:SCC0131
UDSCC2
93VU147T
HPV-negative HNSCC cell lines
HPV-positive HNSCC cell lines

Figure S1b

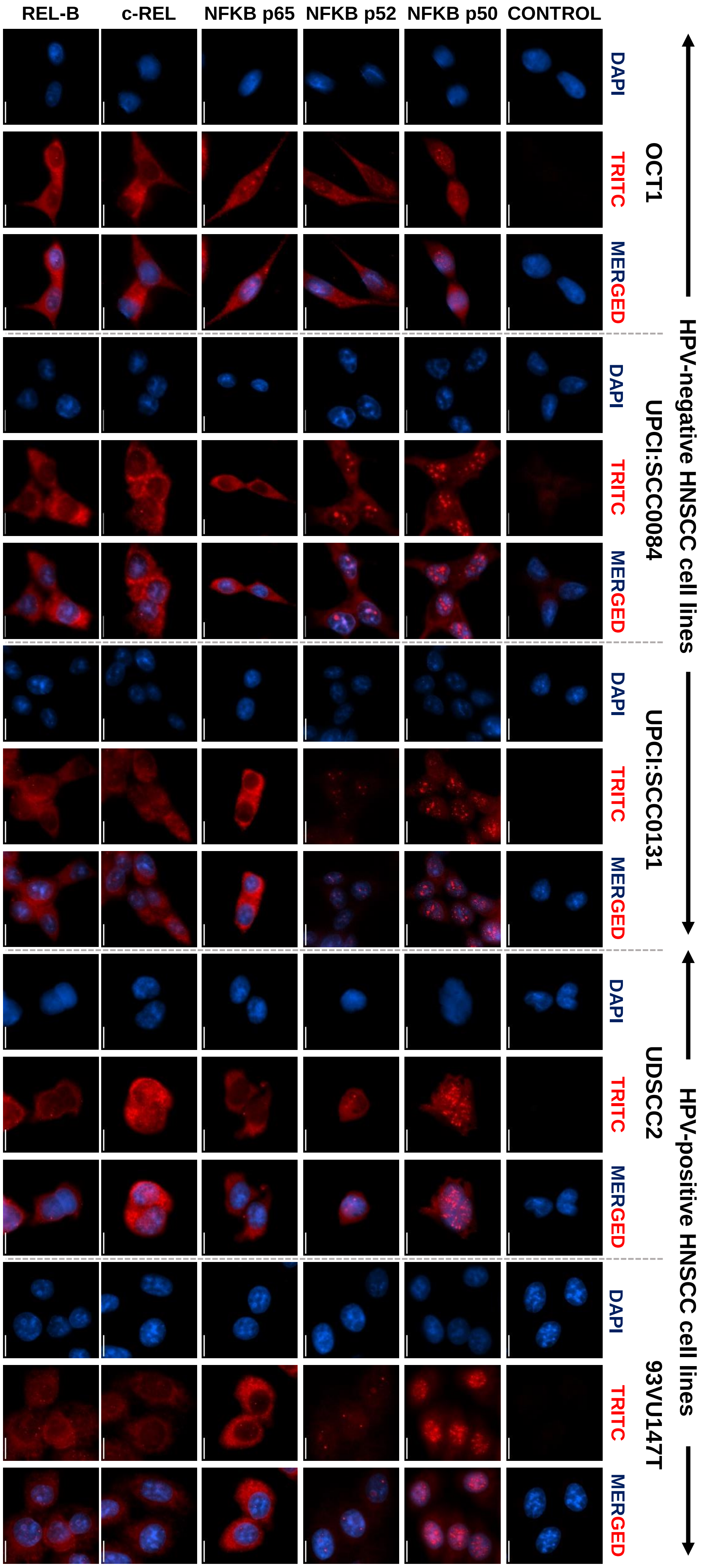
REL-B
c-REL
NFKB p65
NFKB p52
NFKB p50
CONTROL
DAPI
TRITC
MERGED
OCT1
UPCI:SCC0084
UPCI:SCC0131
UDSCC2
93VU147T
HPV-negative HNSCC cell lines
HPV-positive HNSCC cell lines

Figure S1c

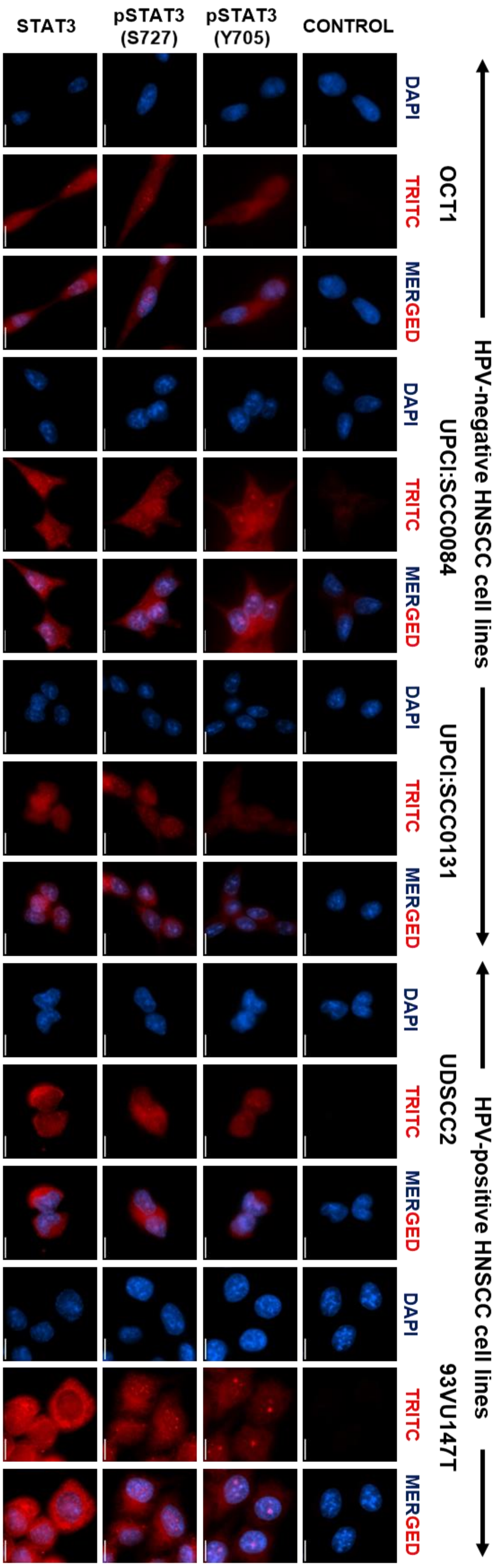

STAT3
pSTAT3 (S727)
pSTAT3 (Y705)
CONTROL
DAPI
TRITC
MERGED
OCT1
UPCI:SCC0084
UPCI:SCC0131
HPV-negative HNSCC cell lines
UDSCC2
93VU147T
HPV-positive HNSCC cell lines

Figure S1d

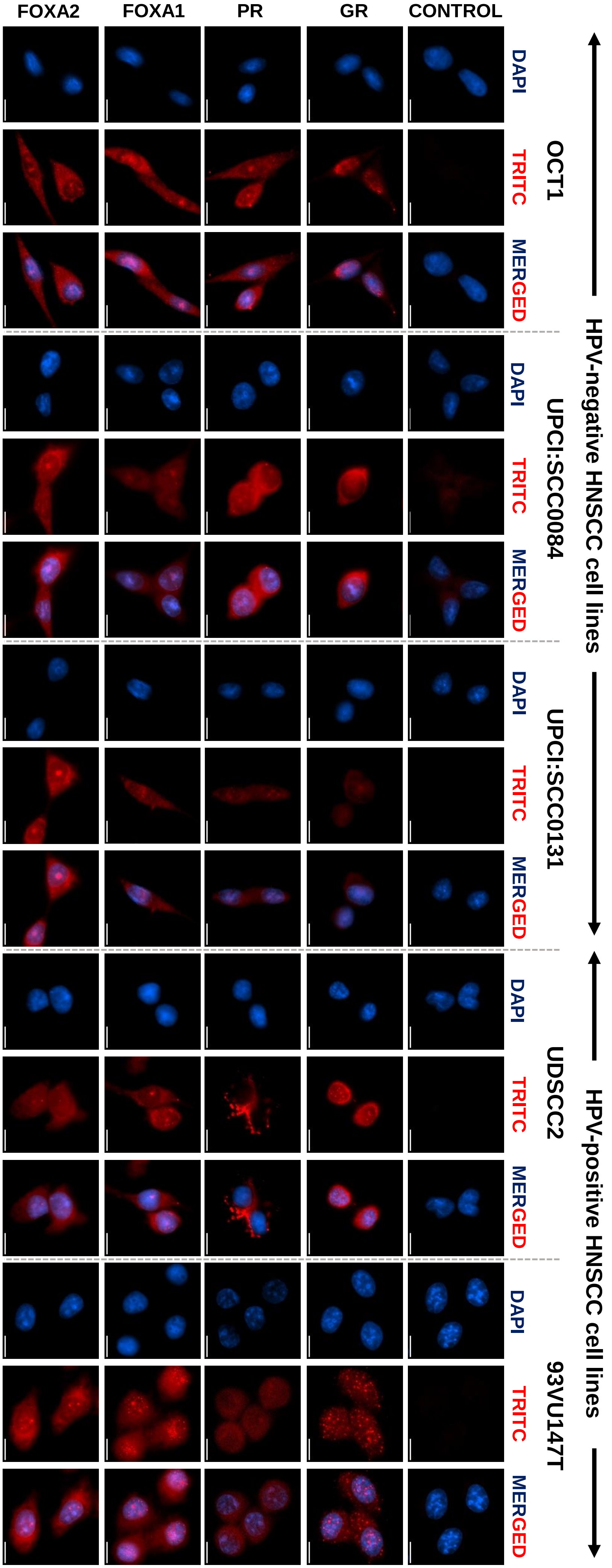
FOXA2
FOXA1
PR
GR
CONTROL
DAPI
TRITC
MERGED
OCT1
UPCI:SCC0084
UPCI:SCC0131
UDSCC2
93VU147T
HPV-negative HNSCC cell lines
HPV-positive HNSCC cell lines

Figure S1e

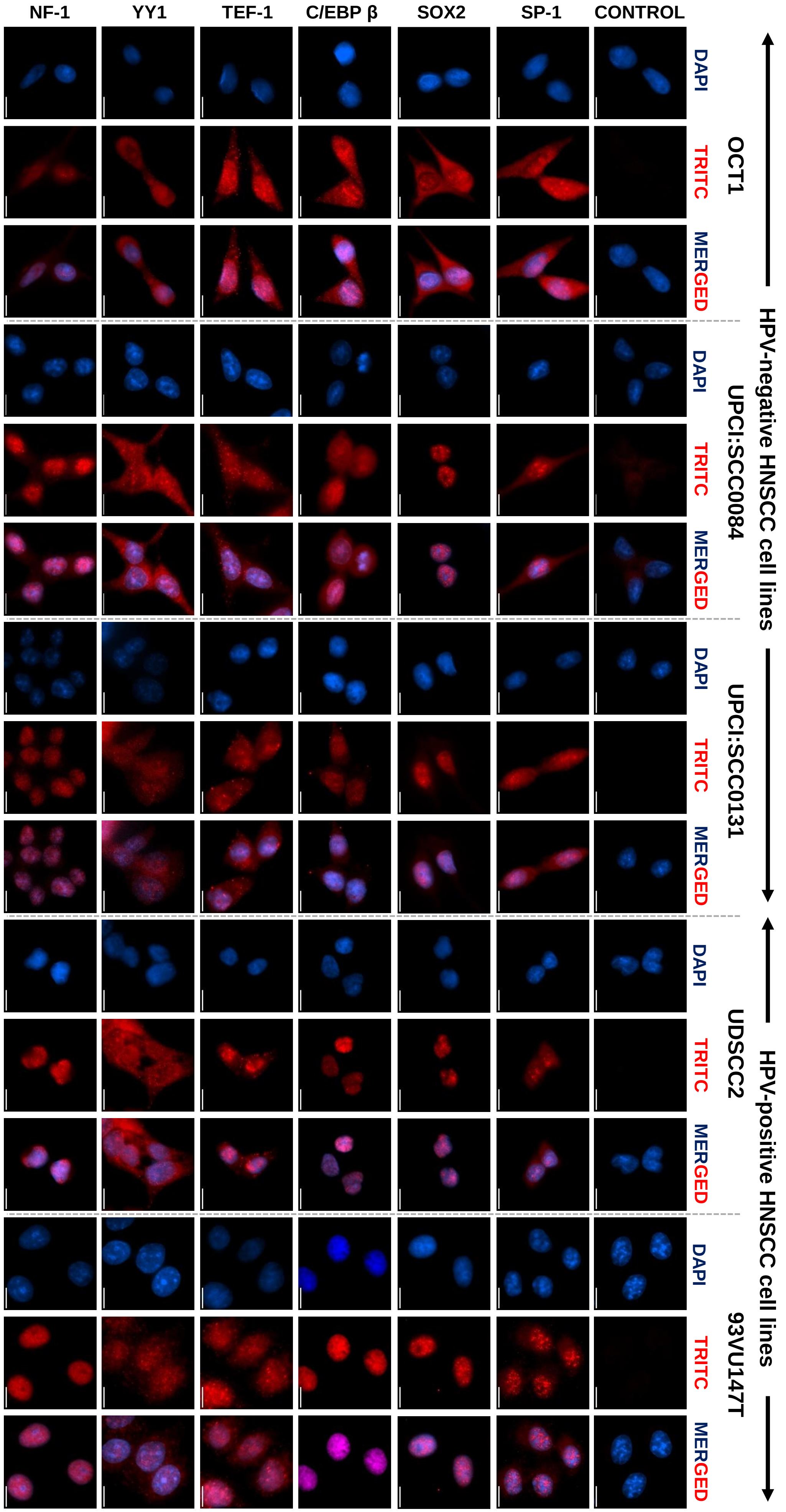

Figure S1: Representative fluorescence photomicrographs showing subcellular localization of LCR-associated transcription factors in HNSCC cell lines. (a) AP-1 family (b) NF-κB family (c) STAT3 and its phosphorylated forms (d) Other inducible transcription factors (e) Constitutive transcription factors *(High resolution images for Figure 3A)*

Figure S2a

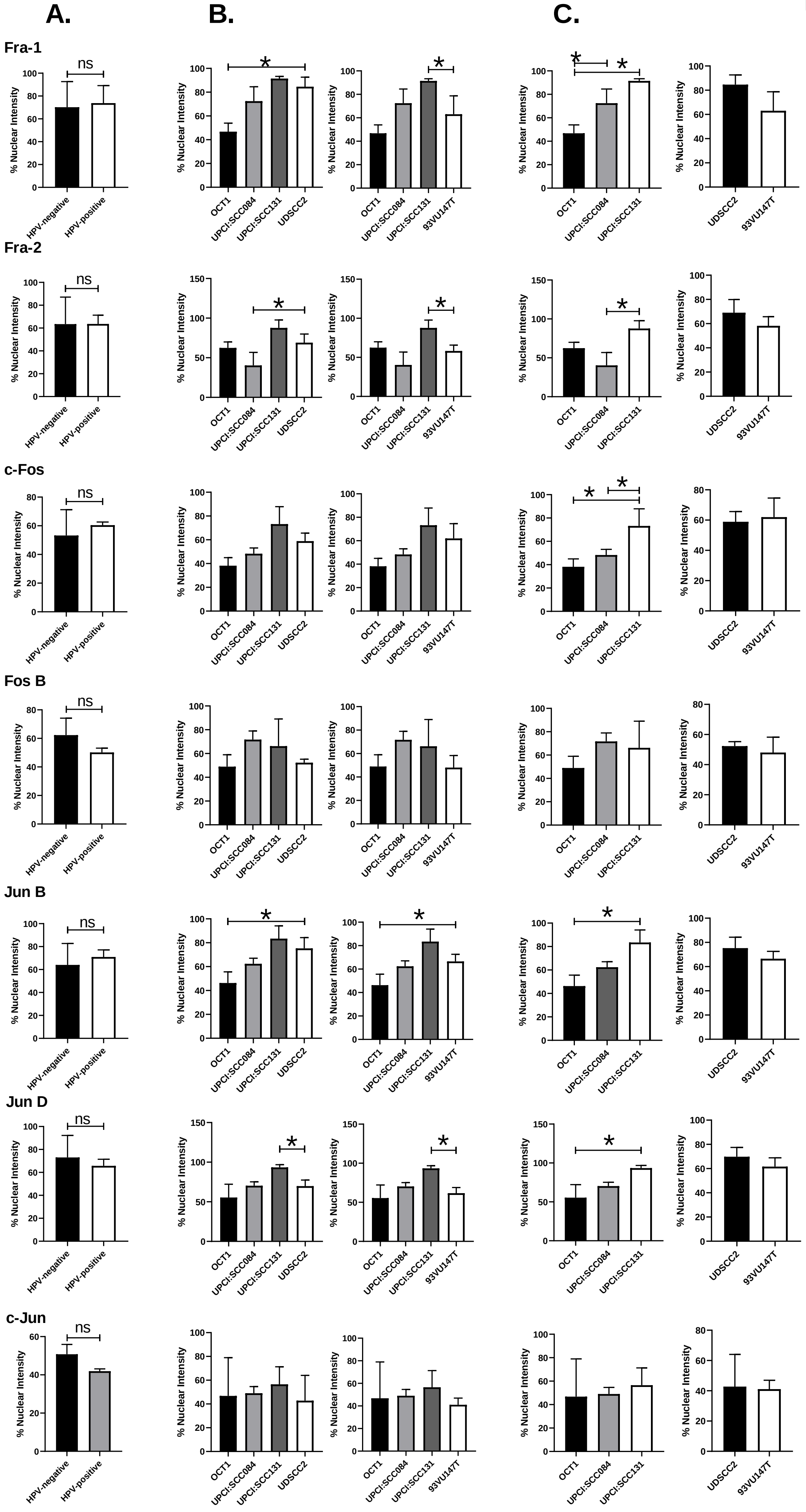
A.
B.
C.
Fra-1
Fra-2
c-Fos
Fos B
Jun B
Jun D
c-Jun
% Nuclear Intensity
ns
*
HPV-negative
HPV-positive
OCT1
UPCI:SCC084
UPCI:SCC131
UDSCC2
93VU147T

Figure S2b

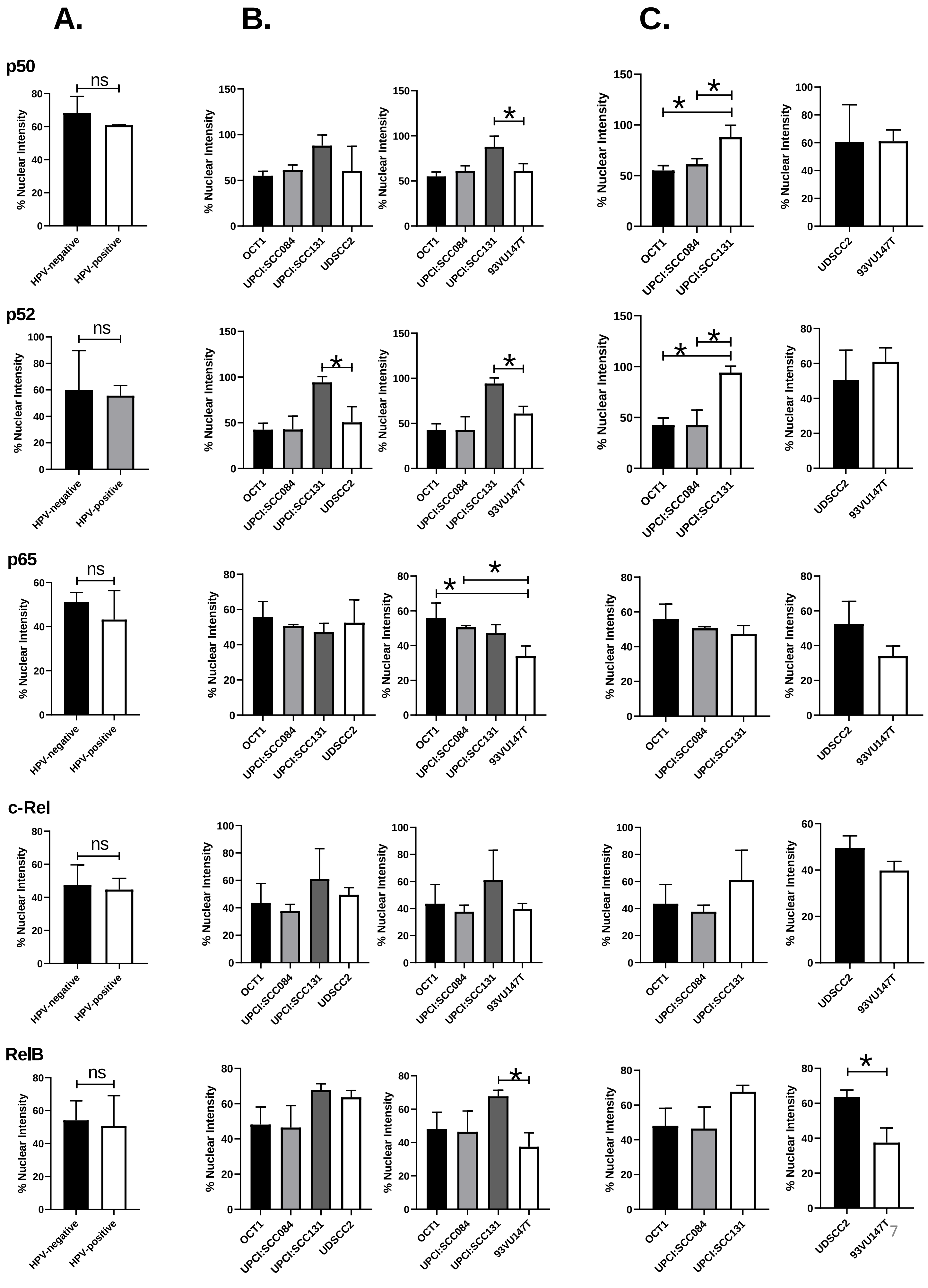
A.
B.
C.
p50
p52
p65
c-Rel
RelB
ns
*
% Nuclear Intensity
HPV-negative
HPV-positive
OCT1
UPCI:SCC084
UPCI:SCC131
UDSCC2
93VU147T

Figure S2c

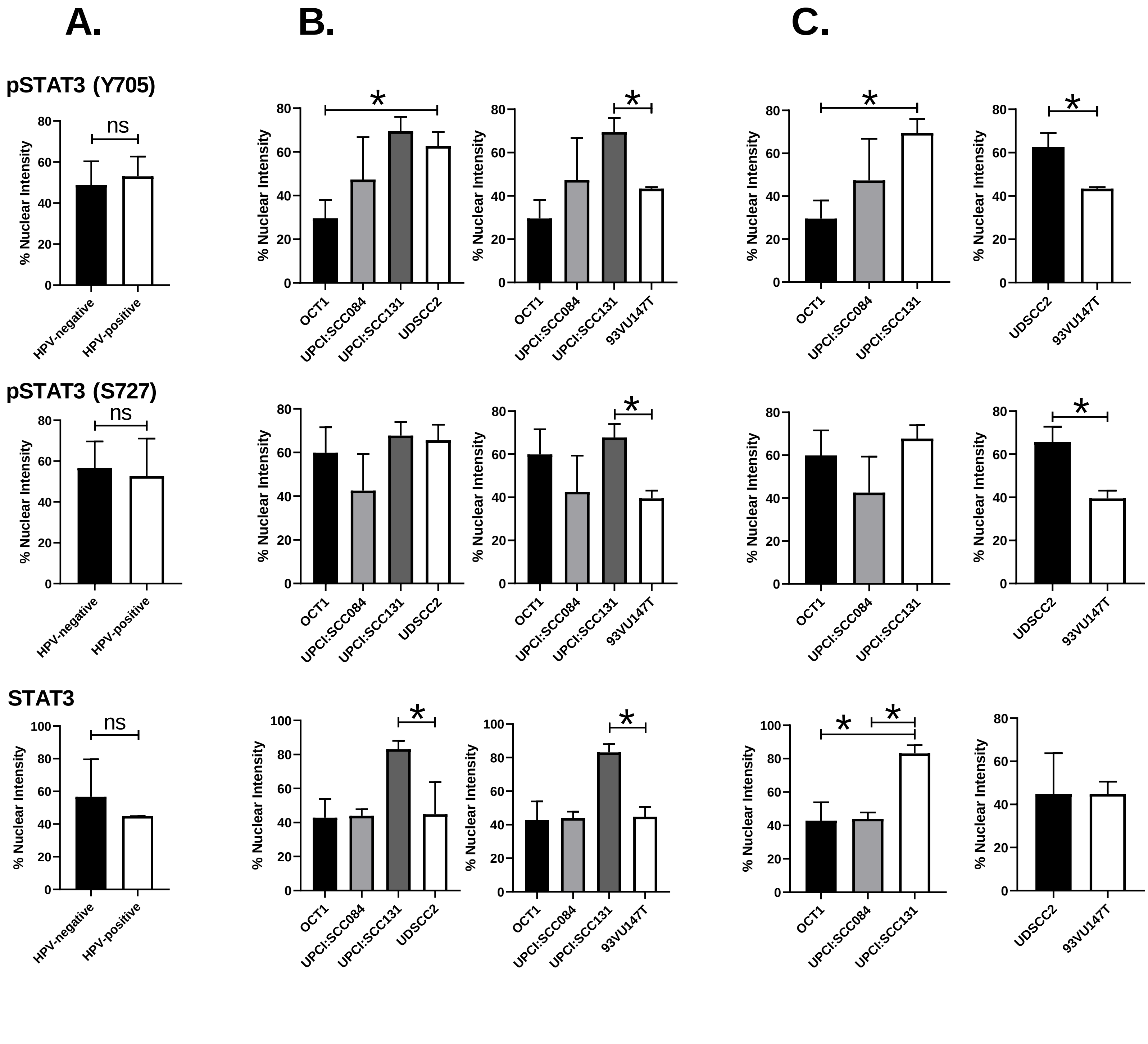
A.
B.
C.
pSTAT3 (Y705)
pSTAT3 (S727)
STAT3
% Nuclear Intensity
ns
*
HPV-negative
HPV-positive
OCT1
UPCI:SCC084
UPCI:SCC131
UDSCC2
93VU147T

Figure S2d

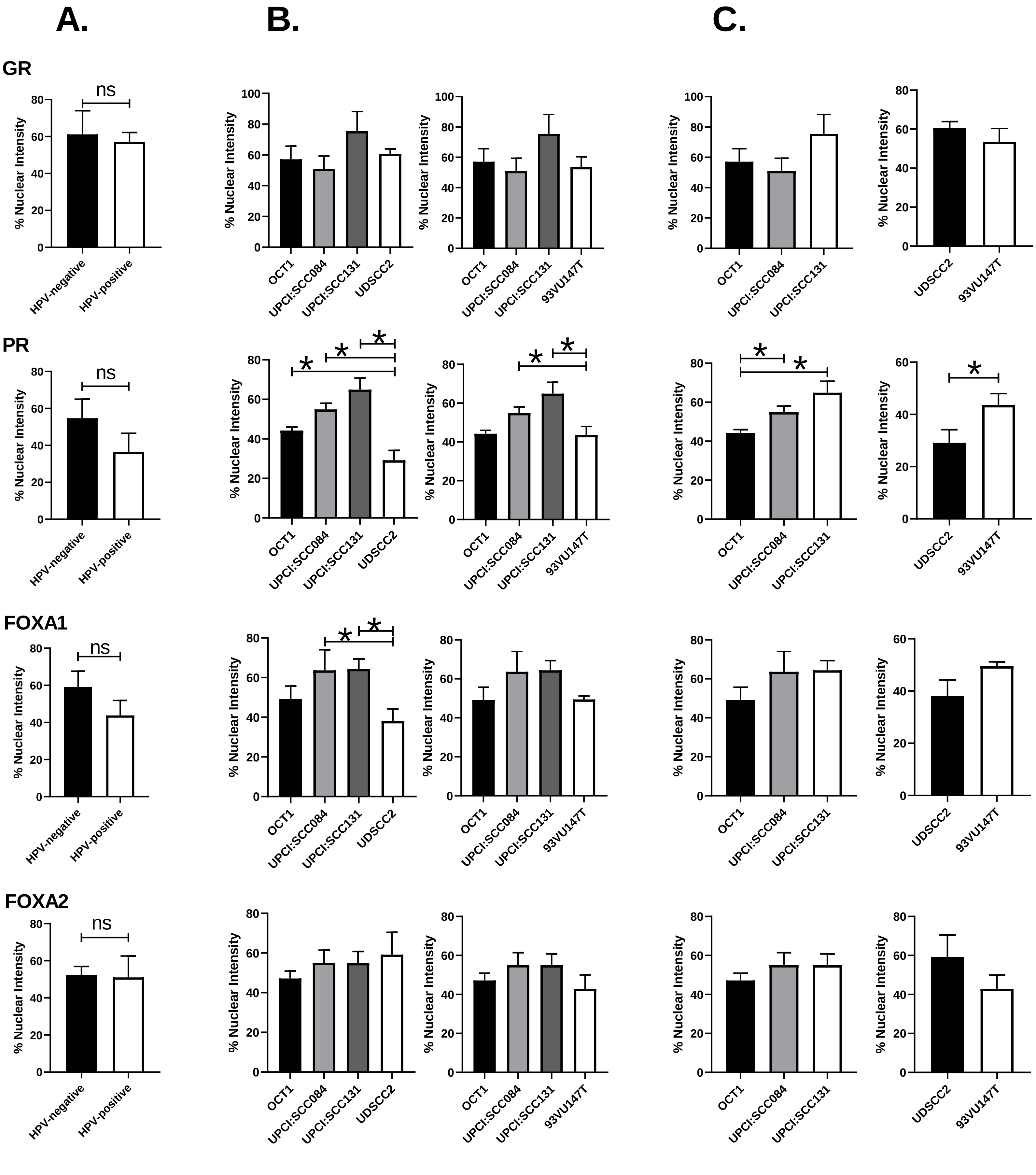
A.
B.
C.
GR
PR
FOXA1
FOXA2
ns
*
% Nuclear Intensity
HPV-negative
HPV-positive
OCT1
UPCI:SCC084
UPCI:SCC131
UDSCC2
93VU147T

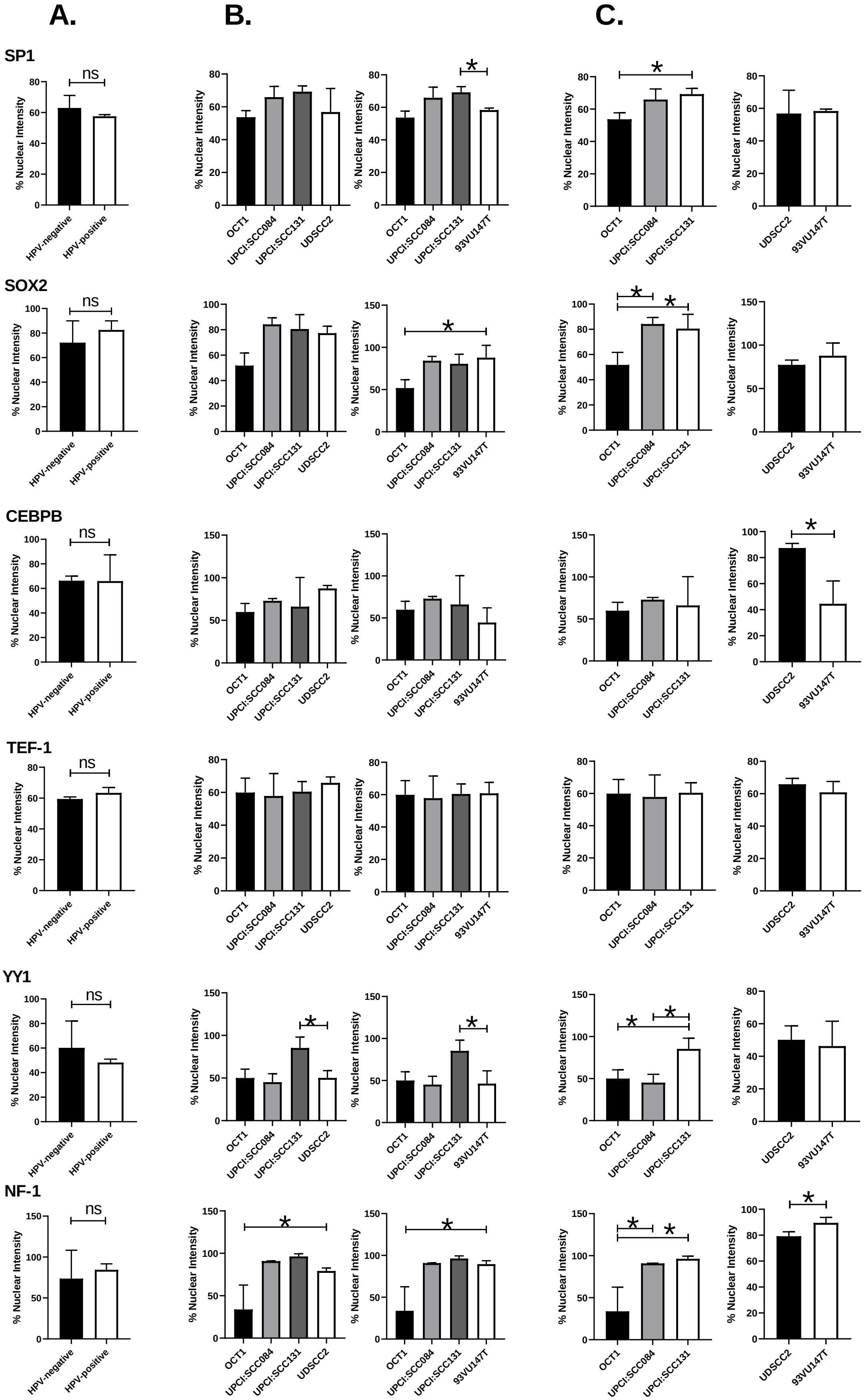

Figure S2:Quantitative estimation of nuclear positivity of LCR-associated transcription factors in HNSCC cell lines. a. AP-1 family **b.** NF-κB family **c.** STAT3 **d.** Other inducible transcription factors **e.** Constitutively-active transcription factors. **A.** The bar graph represents the aggregated normalized nuclear intensity of LCR-associated transcription factor family members that were grouped into HPV-positive and HPV-negative as per the HPV status of the cells. Data plotted as mean band intensity of LCR-associated transcription factor members ± SEM (n=3 for HPV-negative and n=2 for HPV-positive) of the representative experiment out of three independent experiments *p value <0.05. **B.** The bar graphs represent the percentage nuclear positivity of LCR-associated transcription factor family members in HPV-negative HNSCC cells w.r.t. individual HPV-positive HNSCC cells (left panel: UDSCC2 and right panel: 93VU147T). **C.** The bar graphs represent the percentage of nuclear positivity of LCR-associated transcription factor family members within HPV-negative HNSCC cells (left panel) and HPV-positive HNSCC cells (right panel). Data plotted as mean of percentage nuclear positivity ± SD of the representative experiment out of three independent experiments **p value <0.05*.

Figure S3

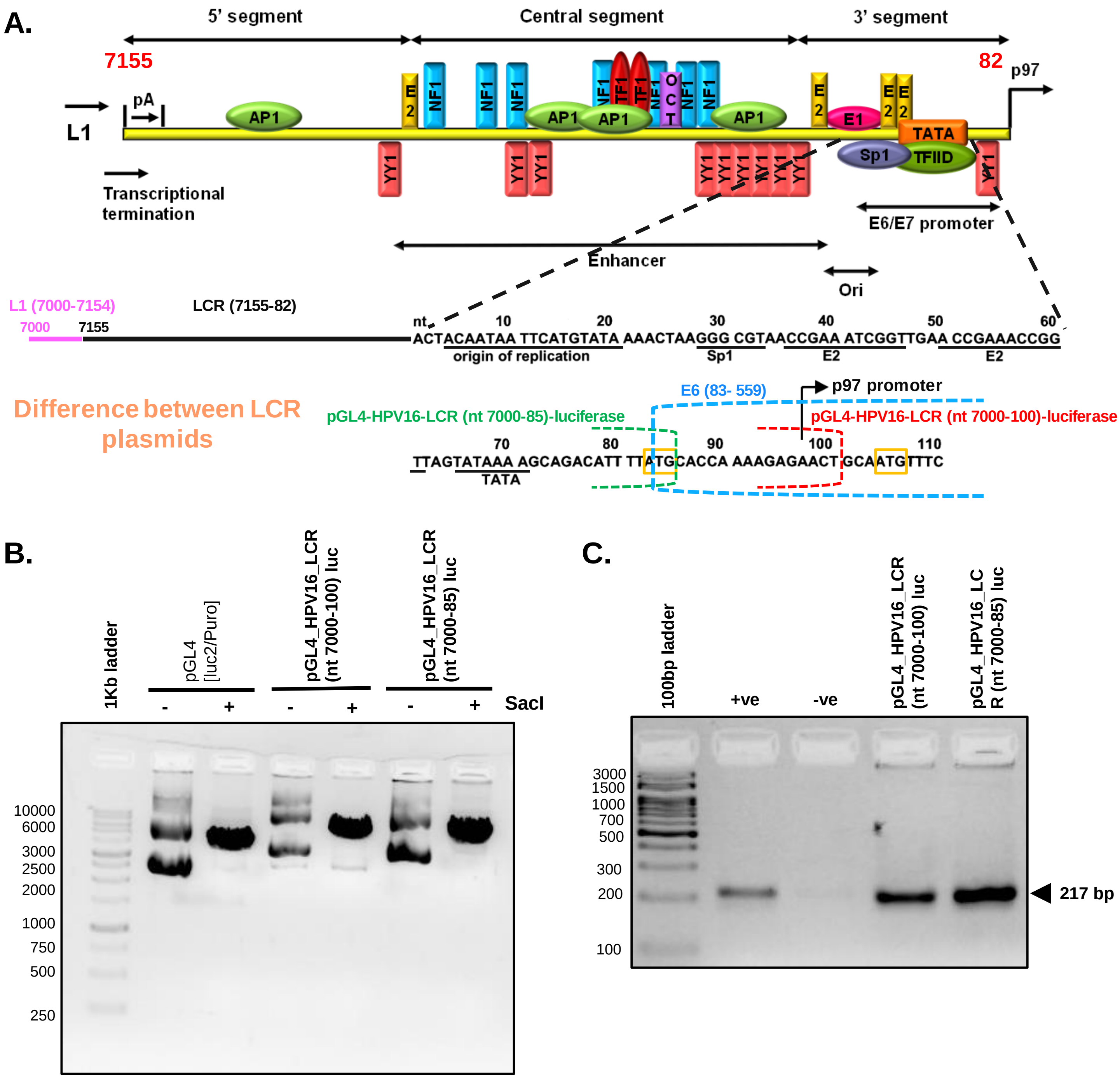

Figure S3: HPV16-LCR plasmids structural analysis, expansion and validation. A. Schematic representation of the key differences in the vector pGL4-LCR-HPV16 (nt 7000-100) and pGL4-LCR-HPV16 (nt 7000-85). **B.** Images of the LCR plasmids digested with *SacI* with undigested plasmids run on 1% agarose gel to check the expected size of the plasmid. **C.** Images of PCR products run on 2% agarose gel showing amplification of specific amplification of HPV16 LCR DNA in pGL4-LCR-HPV16 (nt 7000-100) and pGL4-LCR-HPV16 (nt 7000-85) plasmid. Positive control: International standard of HPV16 (10,000 copy number/µL). Nuclease-free water was used as a negative control. 100ng DNA was used for each PCR. Marker: 100bp ladder.

Figure S4

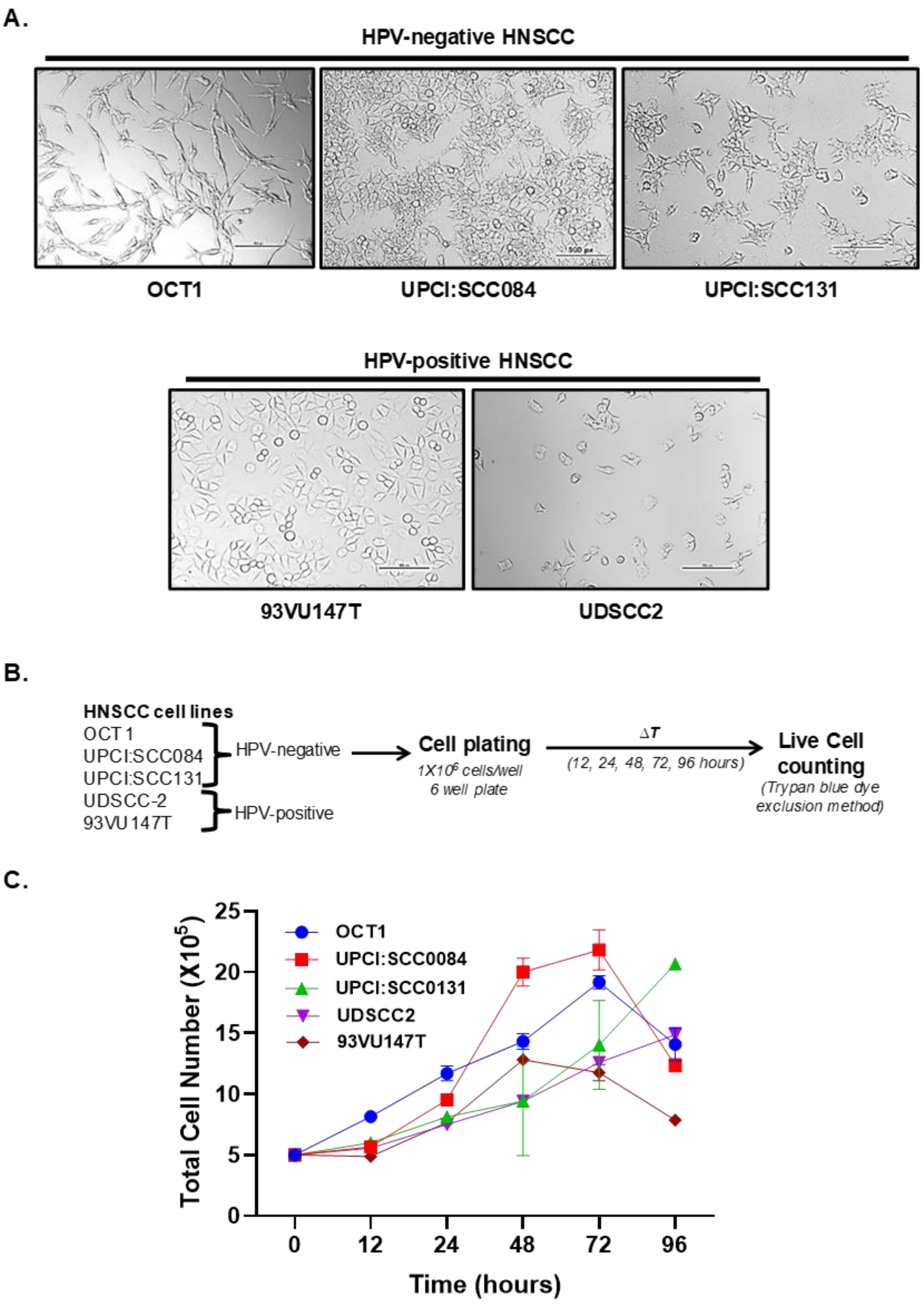

Figure S4: Cell culture characteristics of HNSCC cells used for present study. A. Photomicrographs of cultures of 93UV147T, UDSCC-2 (HPV16-positive) and OCT-1, UPCI:SCC084, UPCI:SCC131 (HPV-negative) captured by ECLIPSE Ti inverted microscope (Nikon) at original magnification, 100X. **B.** Schematic representation of the experiment to check cell growth in HNSCC cells. **C.** Cell growth of HNSCC cell lines. Graph showing the total live cell count of the cultures seeded with initial density of $5x10^5$ cells/well. Values are mean of total cell count ±SD of a representative experiment conducted in triplicate.

Figure S5

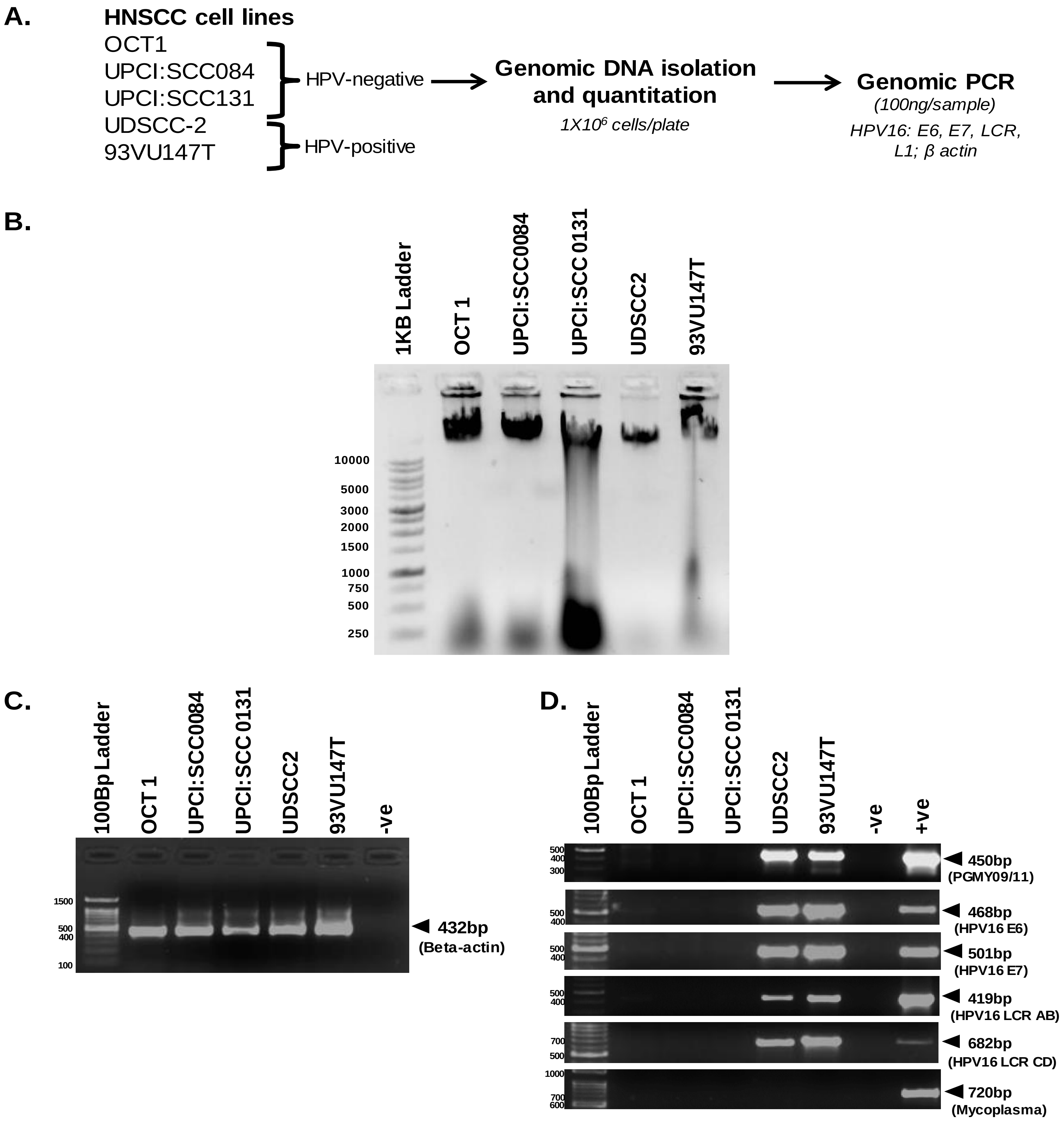

Figure S5: Confirmation of mycoplasma-free cultures and confirmation of HPV-status of HNSCC cell lines using HPV-type specific PCRs. A. Representative image of isolated genomic DNA (1µg/lane) of different HNSCC cells using QIAamp DNA mini kit and run on agarose gel (1%). Marker: 1 Kb ladder. **B.** Images of PCR products run on 2% agarose gel showing amplification of β-actin to check the integrity of genomic DNA. **C.** Images of PCR products run on 2% agarose gel showing amplification of specific amplification of HPV L1 (PGMY09/11), HPV16 E6, E7, LCR, and mycoplasma DNA in HNSCC cell lines. Positive control for HPV-type specific PCRs: International standard of HPV16 (10,000 copy number/µL) and for mycoplasma PCR: known mycoplasma positive cells' DNA. Nuclease free water was used as negative control. 100ng DNA was used for each PCR. Marker: 100bp ladder.

Figure S6

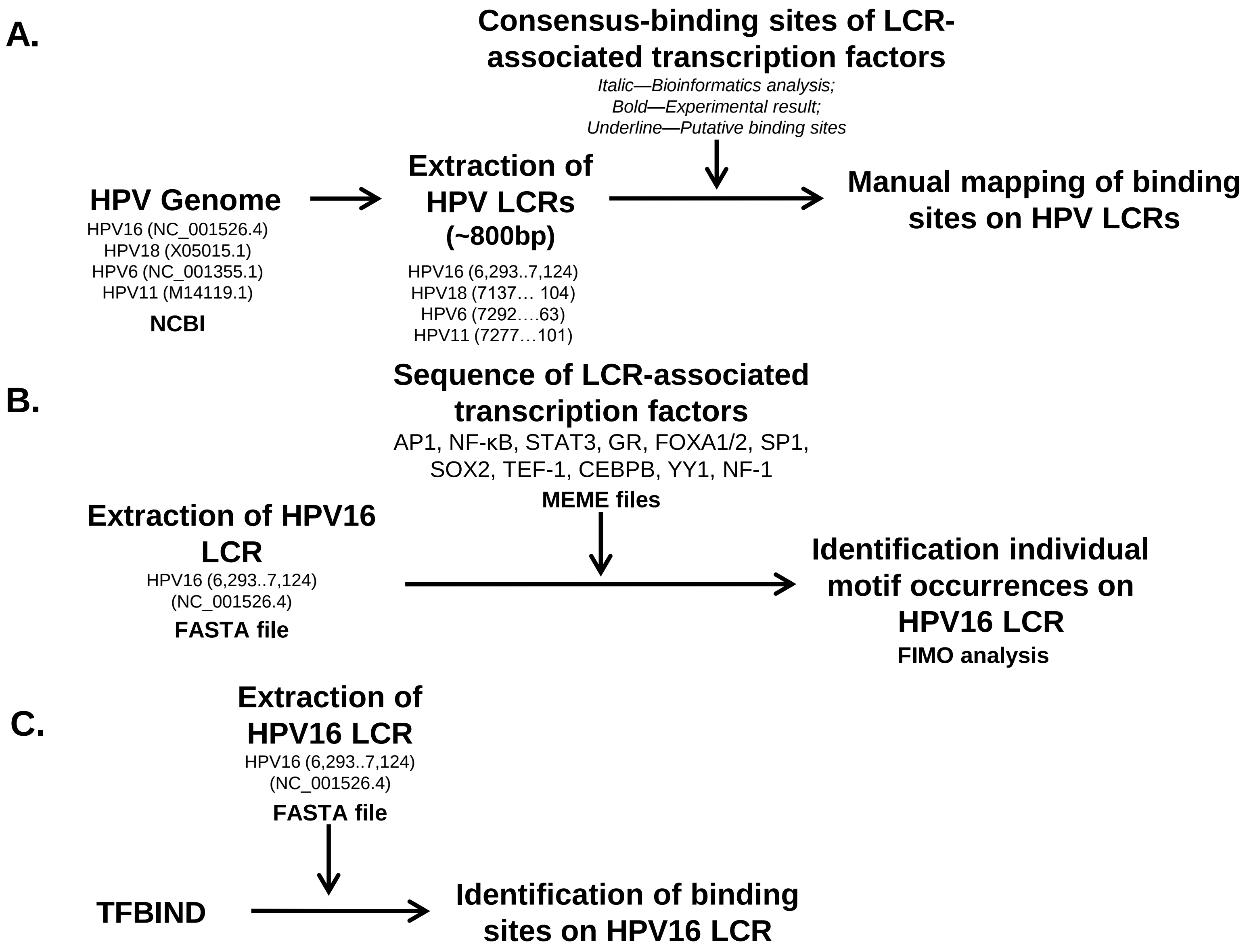

Figure S6: Identification of HPV LCR-associated transcription factor using bioinformatics tools. Schematic representation of the analysis steps followed for manual mapping of transcription factor consensus sites on HPV LCRs (A). Identification of binding sites present on HPV16 LCR using TFBIND (B) and individual motif occurrences of LCR-associated transcription factors on HPV16 LCR using FIMO analysis (C). LCR: Long Control Region.

Figure S7

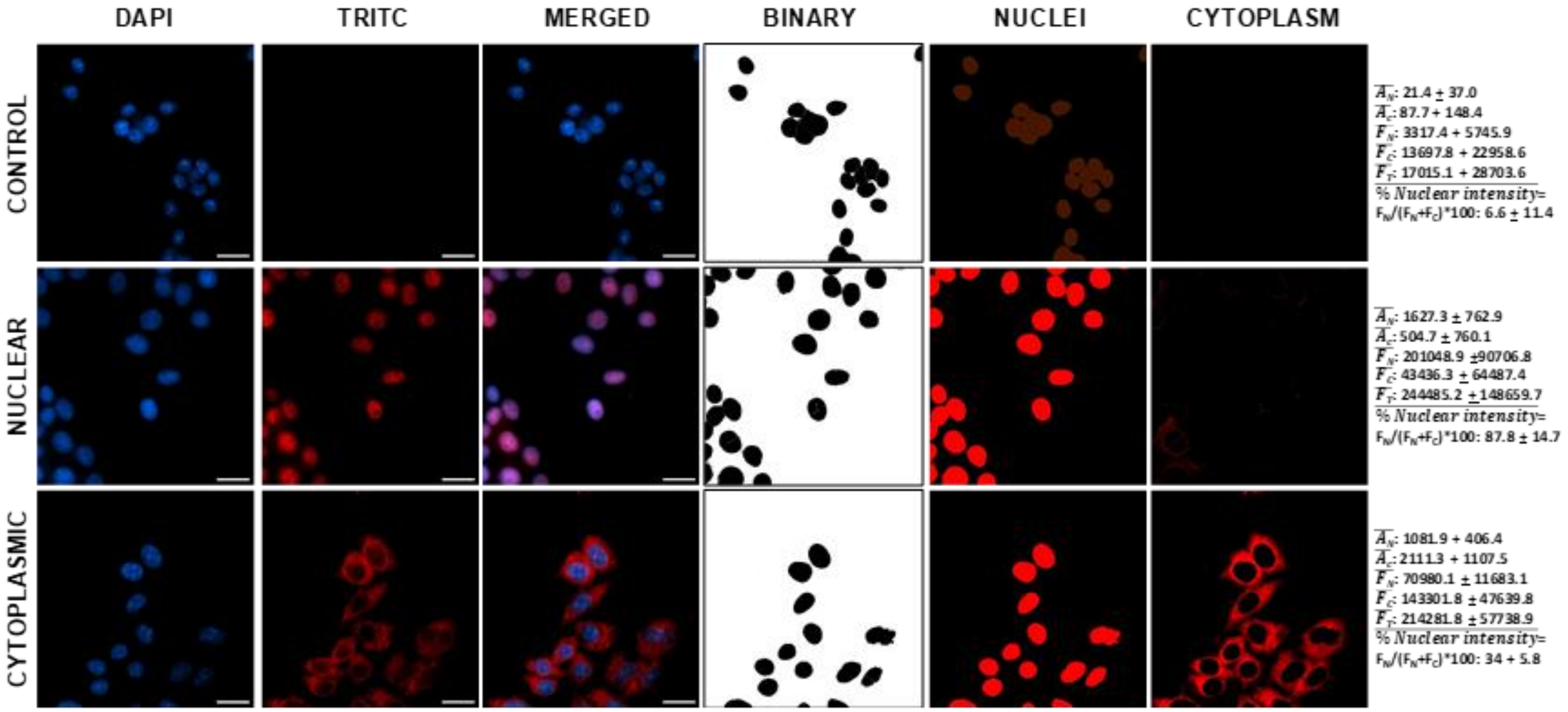

Figure S7: Representative images showing image processing for determination of nuclear localization of respective transcription factors. Representative images of control (upper panel), nuclear (middle panel), and cytoplasmic (lower panel) localization of transcription factor using ImageJ. Binary images were used for the generation of masks to mark the nuclear region of interest. The nuclear mask was overlayed over the cytoplasmic mask to determine the integrated density of the nucleus and cytoplasm, respectively. *(Scale: 20μm).*

Table S1: List of transcription factors motif occurrence on HPV16 LCR (NC_001526.4) using FIMO analysis.

Table S2: List of transcription factors motif occurrence on HPV16 LCR (NC_001526.4) using TFBIND

Table S3: List of different human HNSCC cell lines used in the present study.

Cell line	Tissue of origin	Key Characteristics	Cell Doubling Time (Cellosaurus) (hours)	Culture Media	Source	Ref.
**OCT1**	Lateral Tongue	**HPV-negative**<br>Morphology: epithelial<br>Growth property: adherent	NA	DMEM low glucose	INMAS, DRDO	**(Bhatt *et al.* 2015)**
**UPCI: SCC084**	Oral cavity, retromolar trigone	**HPV–negative**<br>Morphology: epithelial<br>Growth property: adherent<br>Male, 52-year-old	~22	DMEM F12	University of Pittsburgh	**(Telmer *et al.* 2003)**
**UPCI: SCC131**	Floor of mouth	**HPV-negative**<br>Morphology: epithelial<br>Growth property: adherent<br>Male, 73-year-old	~50-60	DMEM F12	University of Pittsburgh	**(White *et al.* 2007)**
**UDSCC2**	Hypophary nx	**HPV16-positive**<br>Morphology: epithelial<br>Growth property: adherent<br>Male, 58-year-old	42	MEM	University of Dusseldorf	**(Ballo *et al.* 1999)**
**93VU147T**	Floor of mouth	**HPV16-positive**<br>Morphology: epithelial<br>Growth property: adherent<br>Male, 58-year-old	52	DMEM high glucose	Vrije Universiteit	**(Steenb ergen *et al.* 1995)**

Abbreviations: DMEM: Dulbecco's Modified Eagle's Medium; HPV: Human Papillomavirus; INMAS: Institute of Nuclear Medicine & Allied Sciences-DRDO; MEM: Minimum Essential Medium; NA: Not available

Table S4: List of primers used for genomic and reverse transcriptase PCR, and their key characteristics.

Primer sets (Lab ID)	Target	PCR Type	Primer sequence	Amplicon size (bp)	Annealing temp. (°C)
**β –actin** (P1019A P1019B)	Human β -actin	Genomic	F 5'-CGGCCAGCCAGGTCCAGACG-3'<br>R 5'-CCAGGCACCAGGGCGTGATG-3'	436	55
**Mycoplasma** (P1014A, P1014B)	Mycoplasma	Genomic	F 5'-ACTCCTACGGGAGGCAGCAGTA-3'<br>R 5'-TGCACCATCTGTCACTCTGTTAACCTC-3'	720	55
**HPV16 E6** (P1008A P1008B)	E6	Genomic	F 5'-GAAACCGGTTAGTATAAAAGCAGAC– 3'<br>R 5'-AGCTGGGTTTCTCTACGTGTTCT– 3'	468	55
**HPV16 E7** (P1009A P1009B)	E7	Genomic	F 5'-CCATAATATAAGGGGTCGGTGGA-3'<br>R 5'-TTTTTCACTACAGCCTCTACAT-3'	501	55
**PGMY09/11** (P1001A-R)	L1	Genomic	F: PGMY09-F: 5'-CGTCCCAAAGGAAACTGATC-3'<br>PGMY09-G: 5'-CGACCTAAAGGAAACTGATC-3'<br>PGMY09-H: 5'-CGTCCAAAAGGAAACTGATC-3'<br>PGMY09-I: 5'-GCCAAGGGGAAACTGATC-3'<br>PGMY09-J: 5'-CGTCCCAAAGGATACTGATC-3'<br>PGMY09-K: 5'-CGTCCAAGGGGATACTGATC-3'<br>PGMY09-L: 5'-CGACCTAAAGGGAATTGATC-3'<br>PGMY09-M: 5'-CGACCTAGTGGAAATTGATC-3'<br>PGMY09-N: 5'-CGACCAAGGGGATATTGATC-3'<br>PGMY09-P: 5'-GCCCAACGGAAACTGATC-3'<br>PGMY09-Q: 5'-CGACCCAAGGGAAACTGGTC-3'<br>PGMY09-R: 5'-CGTCCTAAAGGAAACTGGTC-3'<br>R: PGMY11-A: 5'-GCACAGGGACATAACAATGG-3'<br>PGMY11-B: 5'-GCGCAGGGCCACAATAATGG-3'<br>PGMY11-C: 5'-GCACAGGGACATAATAATGG-3'<br>PGMY11-D: 5'-GCCCAGGGCCACAACAATGG-3'<br>PGMY11-E: 5'-GCTCAGGGTTTAAACAATGG-3'	450	55
**LCR AB** (P1259A P1259B)	LCR	Genomic	F 5'-CCTCATCTACCTCTACAACTGCTAAACGG-3'<br>R 5'-GTTTAAACCATAGTTGCTGACATAGAAC-3'	419	56
**LCR CD** (P1259C P1259D)	LCR	Genomic	F 5'-GCTTCAACCGAATTCGGTTGCATG-3'<br>R 5'-CGTCGCAGTAACTGTTGCTTGCAGTACACAC-3'	682	56
**RT-HPV16 E6** (P1290A P1290B)	HPV16 E6	Transcript	F 5'-AATGTTTCAGGACCCTACGG-3'<br>R 5'-TCAGGACACAGTGGCTTTTG-3'	157	55
**RT-HPV16 E7** (P1291 A P1291 B)	HPV16 E7	Transcript	F 5'-TTTGCAACCAGAGACAACTGA-3'<br>R 5'-GCCCATTAACAGGTCTTCCA-3'	214	55
**RT-GAPDH** (P1284 A P1284 B)	Human GAPDH	Transcript	F 5'-GTCTCCTCTGACTTCAACAGCG-3'<br>R5'-ACCACCCTGTTGCTGTAGCCAA-3'	131	60

Abbreviations: bp: base pair; β-actin: Beta-Actin; E6/E7: Early protein 6/7; GAPDH: Glyceraldehyde 3-phosphate dehydrogenase; HPV: Human Papillomavirus; LCR: Long Control Region.

Table S5: List of antibodies used for immunoblotting and immunocytochemistry experiments.

Antibodies (Lab ID)	Source (Cat. No.)	Origin and type	Dilution*	Dilution#
**HPV 16/18 E6** (#397)	Santa Cruz (SC-460)	Mouse monoclonal IgG	1:5000	1:100
**HPV 16 E7** (#356)	Abcam (ab-20191)	Mouse monoclonal IgG	1:5000	1:100
**Fra-1** (#40)	Santa Cruz (SC-605)	Rabbit polyclonal IgG	1:5000	1:100
**Fra-2** (#51)	Santa Cruz (SC-171)	Rabbit polyclonal IgG	1:5000	1:100
**c-Fos** (#49)	Santa Cruz (SC-7202)	Rabbit polyclonal IgG	1:5000	1:100
**Fos-B** (#54)	Santa Cruz (SC-48)	Rabbit polyclonal IgG	1:5000	1:100
**JunB** (#55)	Santa Cruz (SC-73)	Rabbit polyclonal IgG	1:5000	1:100
**JunD** (#48)	Santa Cruz (SC-74)	Rabbit polyclonal IgG	1:5000	1:100
**c-Jun** (#288)	Santa Cruz (SC-45)	Rabbit polyclonal IgG	1:5000	1:100
**NF-κB p50**(#18)	Santa Cruz (SC-7178)	Rabbit polyclonal IgG	1:5000	1:100
**NF-κB p52** (#22)	Santa Cruz (SC-298)	Rabbit polyclonal IgG	1:5000	1:100
**NF-κB p65** (#404)	Santa Cruz (SC-8008)	Mouse monoclonal IgG	1:5000	1:100
**c-Rel** (#28)	Santa Cruz (SC-70)	Rabbit polyclonal IgG	1:5000	1:100
**RelB** (#26)	Santa Cruz (SC-226)	Rabbit polyclonal IgG	1:5000	1:100
**pSTAT3 (Y705)** (#132)	BD Bio. (612543)	Mouse monoclonal IgG	1:5000	1:100
**pSTAT3 (s727)** (#211)	BD Bio. (612357)	Mouse Monoclonal IgG	1:5000	1:100
**STAT3** (#134)	BD Bio.(610190)	Mouse monoclonal IgG	1:5000	1:100
**GR** (#407)	Santa Cruz (SC-393232)	Mouse monoclonal IgG	1:5000	1:100
**PR** (#408)	Santa Cruz (SC-166169)	Mouse monoclonal IgG	1:5000	1:100
**FOXA1** (#409)	Santa Cruz (SC-101058)	Mouse monoclonal IgG	1:5000	1:100
**FOXA2** (#402)	Santa Cruz (SC-374376)	Mouse monoclonal IgG	1:5000	1:100
**SP1** (#405)	Santa Cruz (SC-17824)	Mouse monoclonal IgG	1:5000	1:100
**SOX2** (#406)	Santa Cruz (SC-365823)	Mouse monoclonal IgG	1:5000	1:100
**CEBPB** (#401)	Santa Cruz (SC-7962)	Mouse monoclonal IgG	1:5000	1:100
**TEF-1** (#403)	Santa Cruz (SC-393976)	Mouse monoclonal IgG	1:5000	1:100
**YY1** (#78)	Santa Cruz (SC-1703)	Rabbit polyclonal IgG	1:5000	1:100
**NF-1** (#86)	Santa Cruz (SC-5567)	Rabbit polyclonal IgG	1:5000	1:100
**Beta Actin** (#383)	Santa Cruz (SC-47778)	Mouse monoclonal IgG	1:10000	-
**Anti-mouse-HRP** (#395)	Santa Cruz (SC-2031)	Goat anti-mouse IgG	1:5000	-
**Anti- Rabbit-HRP** (#362)	Santa Cruz (SC-2030)	Goat anti-rabbit IgG	1:5000	-
**Anti-mouse-AlexaFluor 594** (#268)	Invitrogen (A11005)	Goat polyclonal IgG	-	1:250
**Anti-rabbit-AlexaFluor 594** (#269)	Invitrogen (A11037)	Goat polyclonal IgG	-	1:250

*****Dilution in Immunoblotting #Dilution in Immunocytochemistry **Abbreviation:** CEBPB: CCAAT enhancer binding protein beta; c-Fos: proto-oncogene c-FBJ murine osteosarcoma; E6/E7: Early protein 6/7; Fos-B: FBJ murine osteosarcoma viral oncogene homolog B; FOXA: Forkhead box A; Fra-1: Fos-related antigen 1; GR: Glucocorticoid receptor; HPV: Human Papillomavirus; HRP: Horseradish peroxidase; NF-1: Nuclear Factor 1; NF-κB: Nuclear factor kappa B; PR: Progesterone receptor; pSTAT3(Y701): phospho- Signal Transducer And Activator Of Transcription 3; RelB: V-rel reticuloendotheliosis viral oncogene homolog B; SOX2: SRY-related HMG-box 2; SP1: Specificity protein 1; STAT3: Signal Transducer And Activator Of Transcription 3; TEF-1: Translation elongation factor-1; YY1: Yin Yang 1.

Table S6: List of different plasmids used in the present study.

**Plasmids** (Lab ID)	**Source**	**Cat No.**	**Vector Backbone**	**Size**	**Cloning Sites**	**Bacterial Resistance**	**Growth Strain (Temperature)**
**pGL4-HPV16-LCR (nt 7000-85)-Luciferase** (V0020)	Addgene	32888	pGL4	*~5kb*	*SacI BglII*	Ampicillin, 100µg/mL	DH5alpha (37ºC)
**pGL4-HPV16-LCR (nt 7000-100)-Luciferase** (V0021)	Addgene	32889	pGL4	*~5kb*	*SacI BglII*	Ampicillin, 100µg/mL	DH5alpha (37ºC)
**pRL-TK Vector** (V0025)	Promega	E2241	pRL	4045	*BamHI*	Ampicillin, 100µg/mL	DH5alpha (37ºC)
**pGL4.20[luc2/Puro]** (V0024)	Promega	E6751	pGL4	4242	*SacI BglII*	Ampicillin, 100µg/mL	DH5alpha (37ºC)

Abbreviation: DH5alpha: Douglas Hanahan 5 alpha; HPV: Human Papillomavirus; LCR: Long Control Region.

Table S7: List of the reagents used (with their company and catalog number).

Material	Company	Catalog no.
Dulbecco's Modified Eagle Medium (DMEM), High glucose, L-Glutamine, Sodium bicarbonate and Sodium pyruvate	HiMEDIA	AL007A
Dulbecco's Modified Eagle Medium (DMEM), Low glucose, L-Glutamine, Sodium bicarbonate and Sodium pyruvate	HiMEDIA	AL006
Minimum Essential Medium (MEM), w/ Earle's salts, L-Glutamine, Sodium pyruvate, NEAA and Sodium bicarbonate	HiMEDIA	AL047S
Dulbecco's Modified Eagle Medium / Nutrient Mixture F-12 Ham (DMEM / F12, 1:1 mixture) L-Glutamine, HEPES buffer, Sodium pyruvate and Sodium bicarbonate	HiMEDIA	AL140S
Gibco™ Fetal Bovine Serum, qualified, Brazil	Thermo Scientific™	11573397
Antibiotic Antimycotic Solution 100X Liquid, Endotoxin tested	HiMEDIA	A002A
Trypsin Phosphate Versene Glucose (TPVG)	HiMEDIA	TCL022
QIAamp DNA mini kit	QIAGEN	56304
PCR Master Mix	Thermo Scientific™	K0171
100bp DNA Ladder RTU	GeneDirex	DM001-R500
1Kb DNA Ladder RTU	GeneDirex	DM010-R500
RNeasy Mini Kit	QIAGEN	74104
Verso cDNA Synthesis Kit	Thermo Scientific™	AB1453A
Precision Plus Protein Dual Color Standards	Bio-Rad	1610374
Luminol detection kit	Santa Cruz Biotechnology	sc-2048
Fluoroshield™ with DAPI	Merck	F6057
EndoFree Plasmid Maxi Kit	Qiagen	12362
SacI-HF	New England Biolabs	R3156
BamHI-HF	New England Biolabs	R3136
Lipofectamine™ 2000 Transfection Reagent	Invitrogen™	11668019
Dual-luciferase reporter assay system	Promega	E1910

Abbreviations: cDNA: copy DNA; DAPI: (2-(4-Amidinophenyl)-6-indolecarbamidine dihydrochloride); DMEM: Dulbecco's Modified Eagle Medium; DNA: Deoxyribonucleic Acid; HEPES: (4-(2-hydroxyethyl)-1-piperazineethanesulfonic acid); MEM: Minimum Essential Medium Eagle; NEAA: Non-Essential Amino Acid; PCR: Polymerase Chain Reaction; RPMI: Roswell Park Memorial Institute; RTU: Ready to use; TPVG: Trypsin Phosphate Versene Glucose.
