# Supplementary material for: Insights into expression and localization of HPV16 LCR-associated transcription factors and association with LCR activity in HNSCC
Source: Mol Ther Oncol. 2024 Dec 21;33(1):200926. doi: 10.1016/j.omton.2024.200926 (PMC11780949; doi:10.1016/j.omton.2024.200926)
Supplement: Document S2. Article plus supplemental information [file mmc4.pdf]

Molecular Therapy
Oncology
Original Article

Insights into expression and localization of HPV16 LCR-associated transcription factors and association with LCR activity in HNSCC

Nikita Aggarwal,[1] Divya Janjua,[1] Apoorva Chaudhary,[1] Udit Joshi,[1] Tanya Tripathi,[1] Chetkar Chandra Keshavam,[1] Joni Yadav,[1] Arun Chhokar,[1,2] and Alok Chandra Bharti[1]

[1]Molecular Oncology Laboratory, Department of Zoology, University of Delhi (North Campus), New Delhi, India; [2]Department of Zoology, Deshbandu College, University of Delhi, Delhi, India

Human papillomavirus (HPV)-positive head and neck squamous cell carcinoma (HNSCC) encompasses a heterogeneous group of malignancies characterized by diverse clinical manifestations. Notably, HPV-positive HNSCC exhibits a more favorable prognosis, particularly when the virus is transcriptionally active. This study aimed to elucidate the role of key transcription factors in activating the HPV long control region (LCR), responsible for its oncogenic potential. Utilizing immunoblotting and immunofluorescence techniques, we analyzed the expression and nuclear localization of LCR-associated transcription factors in HPV-negative and HPV-positive HNSCC cell lines. High expression of JunB and low expression of Fra-1, pSTAT3(S727), SP1, and SOX2 were observed in HPV-positive HNSCC cells. Transcriptomic analysis corroborated these findings, revealing differential expression of transcription factors in HPV-positive lesions. Moreover, the study identified strong correlation of LCR-specific transcription factors with HNSCC patient survival. Evaluation of HPV16 LCR reporter activity further underscored the heterogeneous nature of HNSCC, with some HPV-negative cell lines exhibiting comparable LCR activity to HPV-positive counterparts. These findings elucidate the intricate regulatory mechanisms underlying HPV-associated HNSCC and provide insights into potential prognostic markers and therapeutic targets.

INTRODUCTION

Squamous cell carcinoma (SCC) originating in the upper aerodigestive tract within the head and neck (HN) represents a diverse array of malignancies, collectively presenting a significant global health challenge. Because of the heterogeneity, HNSCC has been designated with a specific identifier under *International Classification of Diseases*, 10th edition, for the individual anatomical site of origin of the tumor: lip and oral cavity (C00-06), salivary glands (C07-08), oropharynx (C09-10), nasopharynx (C11), hypopharynx (C12-13), and larynx (C32). As per the GLOBOCAN 2020 report, India is at the forefront of global HN cancer incidence, reporting 206,344 cases. Additionally, it registers the highest mortality rate at 115,793 and exhibits the highest 5-year prevalence, with a total of 443,221 cases.[1] Over the last five decades, there has been a noteworthy shift in the incidence and prevalence of SCC in various constituent subsites, attributed to changes in exposure to different known carcinogens and risk factors. Traditionally, HNSCC has been associated with tobacco and alcohol abuse. However, cancer registry data and multiple cohort studies provided evidence that human papillomavirus (HPV) infection has been detected abnormally in the oral cavity and tumor tissues of the HN region.[2–5] Owing to the prominent connection between sexual transmission of HPV and uterine cervix-related cases, HPV-positive tumors are correlated with the changing societal practices and sexual behaviors leading to oro-genital contact or vertical transmissions of HPV.[6]

In a breakdown of HPV types, HPV16 stands out as the predominant HPV infection observed in HNSCC cases.[7] HPV16 singularly constituted 87% of oropharyngeal SCCs (OSCCs), 68% of oral SCCs, and 69% for laryngeal SCCs. These HPV-positive tumors were observed in early stages with well-differentiated histology and no lymph node involvement.[8–10] Notably, they exhibited improved overall and disease-free survival rates.[11–13]

Not every individual who tests positive for oral HPV DNA develops carcinoma, indicating the presence of cellular heterogeneity in the HN region. Moreover, not all individuals encounter high-risk HPV infection in their lifetime. These variations point to individualistic differences in the cellular environment, which may either facilitate or impede productive HPV infection.[14] Despite identifying transcriptionally active HPV infection in various HN sites, a distinct region in these tissues analogous to the well-defined HPV-susceptible squamocolumnar junction in the cervix is absent. Transcriptionally active HPV, recognized either by the presence of viral oncoproteins (E6 and E7) or their transcripts, has been observed in nearly all major HN sites including the oropharyngeal region,[15] oral cavity,[16] larynx,[17] and the

Received 18 April 2024; accepted 18 December 2024;
https://doi.org/10.1016/j.omton.2024.200926.

Correspondence: Alok Chandra Bharti, Molecular Oncology Laboratory, Department of Zoology, University of Delhi (North Campus), Delhi 110007, India
E-mail: alokchandrab@yahoo.com

Published by Elsevier Inc. on behalf of The American Society of Gene and Cell Therapy.
This is an open access article under the CC BY license (http://creativecommons.org/licenses/by/4.0/).

tonsillar region.[18] However, the spectrum of infecting HPV types and their relative prevalence differ in each subsite.

For HPV infection to participate in the process of tumorigenesis, it must surmount numerous barriers and challenges.[6] First and foremost is reaching the target site. During the establishment of HPV infection, the transcriptional milieu of the host plays a pivotal role in regulating the expression of early HPV genes and they hold independent prognostic value for a productive HPV infection. In the absence of these host transcription factors, the virus remains inactive and is either eliminated or maintains its genome within the host cell cycle until the necessary factors are expressed or activated during the cell differentiation stage or due to local inflammation.[19] A specific non-coding region on the HPV genome referred to as the upstream regulatory region, more commonly known as the long control region (LCR). The LCR plays a pivotal role in coupling the HPV life cycle and expression of viral oncoproteins/gene with host's cellular transcriptional milieu. HPV16 LCR contains binding sites of various transcription factors like activator protein-1 (AP-1),[20] nuclear factor-kappa B (NF-κB),[21] SRY Box 2 (SOX2),[22] glucocorticoid receptor (GR)/progesterone receptor (PR),[23] specificity protein 1 (SP1),[24] transcriotional enhancer factor-1 (TEF-1),[25] CCAAT enhancer binding protein beta (CEBPB),[26] forkhead box A1/2 (FOXA1/2),[27] yin yang 1 (YY1),[28] and nuclear factor-1 (NF-1).[29,30] Notably, slight modifications in the sequences within the LCR region may lead to the removal or addition of binding sites for transcription factors, impacting the efficiency of their interaction.[27,31–33]

Apart from the variations in transcription factor binding sites on LCR, the magnitude and duration of viral transcription are determined chiefly by the expression, availability, and activation of regulatory host cell transcription factors. These transcription factors can be broadly categorized as inducible or constitutive, depending on the fluctuations in their expression and activity level within the cell. It is important to note that almost all inducible factors, such as AP-1, NF-κB, signal transducer and activator of transcription (STAT3), GR, PR, and FOXA1/2 reported to have binding sites on HPV16 LCR, exhibit altered expression and activity in HNSCC.[6] Their constitutive activation independently contributes to the carcinogenic inflammation, transformation, and maintenance of cancer stemness in tumor tissues.[34] Similarly, constitutive transcription factors SP1, SOX2, CEBPB, TEF-1, YY1, and NF-1 having binding sites on HPV16 LCR are also differentially expressed and correlate in HN carcinogenesis.

Despite comprehensive literature showing the involvement of key factors in HNSCC, no study has collectively addressed the heterogeneity of the distribution of these transcription factors in HN cancer. There are studies from our and other groups that demonstrate a skewed representation of a set of transcription factors in HPV-positive and HPV-negative HNSCCs.[8,35–38] In the present study, we aimed to profile the expression levels and nuclear positivity of various LCR-specific inducible and constitutive transcription factors in HPV-negative and HPV-positive HNSCC cell lines. Additionally, we explored how this distinct transcriptional milieu influenced the permissiveness of host cellular environment to HPV infection. Likely, the differences in transcription factor expression and activity profile may govern the response of LCR to produce viral oncoproteins apart from controlling the pathology of the lesion, and therefore patients' response to the therapy.

RESULTS

To assess the expression and activity of transcription factors having binding sites on HPV16 LCR in both HPV-positive and HPV-negative HNSCC cell lines

HPV16-positive HNSCC cells showed transcriptionally active infection

We checked HPV16-positive HNSCC cell lines for transcriptionally active HPV infection by evaluating the presence of transcripts of oncogenes E6 and E7 as well as oncoproteins E6 and E7 by RT-PCR, immunoblotting, and immunocytochemistry, respectively as depicted in Figure 1A. RT-PCR was performed on the cDNA of HNSCC cells to detect HPV16E6 and E7 transcripts revealed faint but distinct bands in both UDSCC2 and 93VU147T HNSCC cells (Figure 1B). In contrast, there were no E6 and E7 signals in the cDNA of any HPV-negative HNSCC cells included in the study. Further, immunoblotting for HPV16 oncoproteins E6 and E7 showed a distinct constitutive expression in both, UDSCC2 and 93VU147T HNSCC cells (Figure 1C). However, the level of E6 protein was low in UDSCC2. Similar to the RT-PCR results oncoproteins E6 and E7 were not detected in the protein isolated from HPV-negative cells. In agreement with the above, immunocytochemistry performed for E6 and E7 revealed nuclear and cytoplasmic expression of E6 and E7 oncoproteins in UDSCC2 and 93VU147T cells (Figure 1D). There was no fluorescence signal in HPV-negative OCT1, UPCI:SCC084, or UPCI:SCC131 HNSCC cells.

In silico *analysis of binding sites of LCR-associated transcription factors on HPV16 LCR*

Manual screening of binding sites for various host transcription factors identified, i.e., AP-1, NF-κB, STAT3, GR/PR, FOXA1, FOXA2, SP1, SOX2, CEBPB, TEF-1, YY1, and NF-1, on reference HPV16-LCR region [RefSeq: NC_001526.4 (6,293–7,124)].[6] These transcription factors were categorized into two groups: Inducible transcription factors (AP-1, NF-κB, STAT3, GR/PR, and FOXA1/2) and constitutive transcription factors (SP1, SOX2, CEBPB, TEF-1, YY1, and NF-1). An *in silico* analysis was performed using MEME files of transcription factors that were downloaded from the JASPAR tool and were subjected to FIMO. The analysis showed motif occurrences of various host transcription factors on the LCR of HPV16, a set of 139 sites that resembled with AP-1 (MA0490.3, MA0492.2, MA0099.3, MA1135.2, MA1130.1, and MA1128.1), 27 for NF-κB (MA0105.4, MA0778.1, MA0107.1, MA0101.1, and MA1117.1), 22 for STAT3 (MA0144.3), 23 for GR (MA0113.3), 54 for FOXA1 (MA0148.5), 45 for FOXA2 (MA0047.4), 5 for SP1 (MA0079.5), 33 for SOX2 (MA0143.5), 34 for CEBPB (MA0466.4), 16 for TEF-1 (MA0090.1), 13 for YY1 (MA0095.2), and 17 for NF-1 (MA0670.1) motifs ($p < 0.05$) (Table S1). The MEME file for PR was not available. Upon comparison of these sites with manually curated data, sites for AP-1, GR/PR, SP1, CEBPB, TEF-1, and NF-1

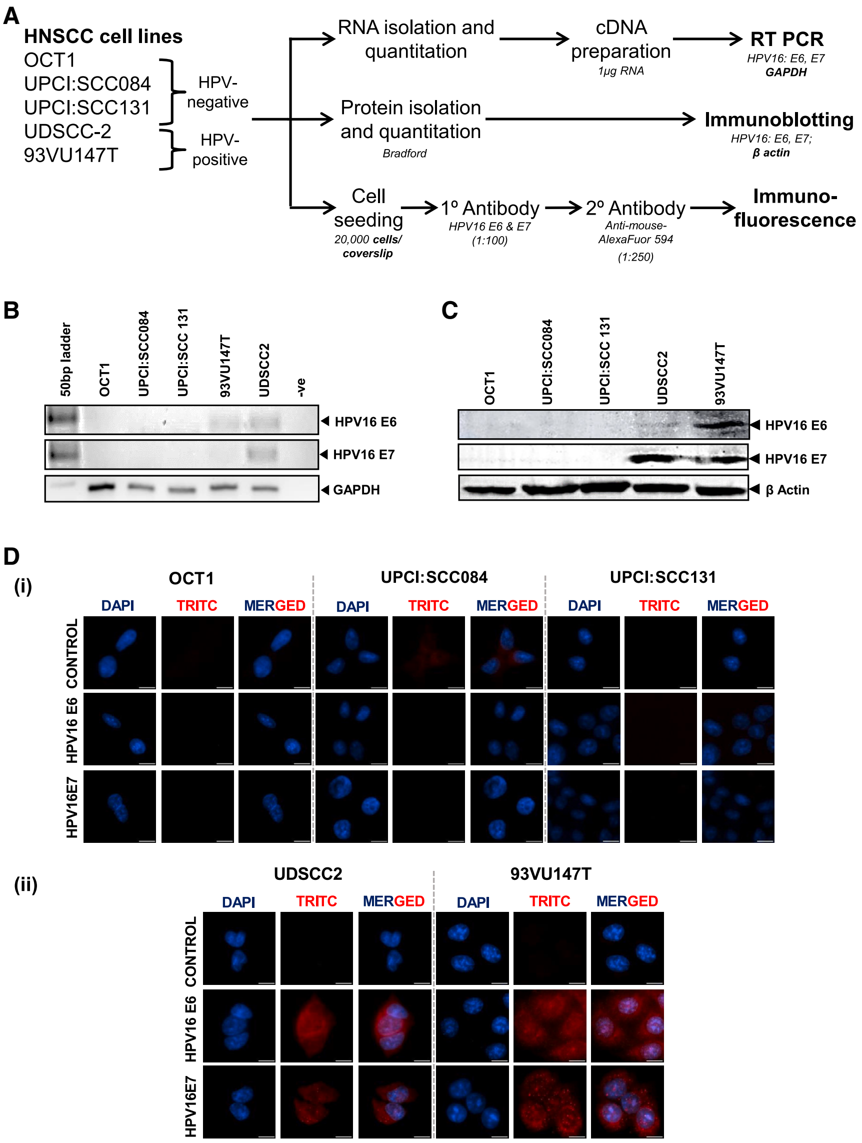

Figure 1. HPV16-positive HNSCC cells showed transcriptionally active infection

(A) Schematic presentation of the experiment. (B) Representative gel photographs of HPV16 E6/E7 and GAPDH RT-PCR products. RT-PCR run on 2% agarose gel. Image showing representative transcript levels in HNSCC cell lines. (C) Representative immunoblots of total protein isolated and detected for HPV16 E6 and E7 from HNSCC cells. Blots were stripped and detected for levels of β-actin protein, as loading control. (D) Detection of HPV16 E6/E7 oncoproteins in HNSCC cell lines by immunocytochemistry. Representative photomicrographs of HNSCC cells were fixed and stained with HPV16 E6 or E7 antibodies and detected with goat anti-mouse IgG-Alexa-594 (Red) and counterstained with DAPI (blue) (scale bar, 10 μm). The data shown is representative of experiments performed at least three times.

showed exact matches, whereas other transcription factors were detected in different locations. Another bioinformatics tool TFBIND identified 1,107 sites on HPV16 LCR (Table S2), which included manual mapped sites for only AP-1, NF-κB, STAT3, FOXA1/2, SP1, CEBPB, YY1, and NF-1 on their respective position, whereas other sites were also seen in different locations, except SOX2 and TEF-1, which could not be detected.

Expression analysis of inducible HPV-related transcription factors in an HNSCC cell line

The expression profiles of various inducible LCR-specific transcription factors (AP-1 family, NF-κB family, STAT3, GR, PR, FOXA1, and FOXA2) was performed in HNSCC cell lines as demonstrated in Figure 2A.

AP-1

Active AP-1 family of transcription factors formed by homodimer of Jun family proteins (JunB, JunD, and c-Jun) or its heterodimer with members of Fos family (Fra-1, Fra-2, FosB, and c-Fos). Immunoblotting for different members of AP-1 revealed a detectable expression of all AP-1 members in both HPV-positive and HPV-negative HNSCC cells (Figure 2B). However, the level of each member differed in the cell lines tested. Densitometric analysis of the band obtained showed level of Fra-1 was particularly high in HPV-negative HNSCC cells and JunB showed a high level in HPV-positive HSNCC cells ($p < 0.05$). Other members of the AP-1 family also showed alteration in the level but the differences were statistically not significant.

NF-κB

The mammalian NF-κB family includes NF-κB1 (p50), NF-κB2 (p52), RelA (p65), RelB, and c-Rel. Immunoblotting for different members of NF-κB revealed a detectable expression in HPV-positive and HPV-negative HNSCC cells (Figure 2C). OCT1 uniformly showed low levels of all the members. The levels of p105 precursor of p50 and p100 precursor of p52 were characteristically very high in the rest of the cell lines. There were no HPV infection-associated specific differences in any member of the NF-κB family. Nevertheless, UPCI:SCC084 and UPCI:SCC131 showed a high level of p65. 93VU147T uniquely had a high level of c-Rel.

STAT3

STAT3 is a widely recognized oncogene and is known for its involvement in promoting epithelial carcinogenesis. Immunoblotting revealed a detectable expression of STAT3 in both HPV-positive and

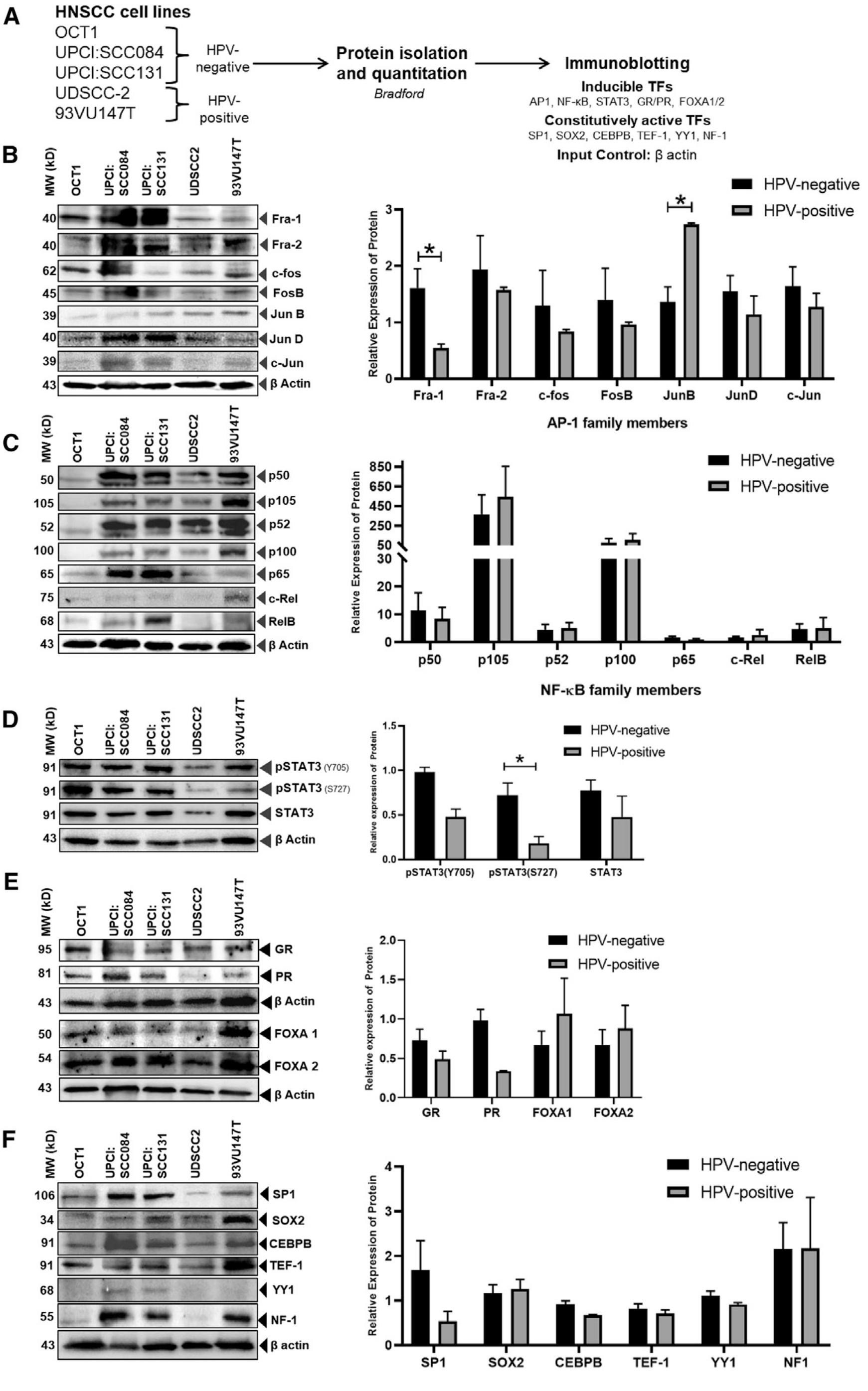

Figure 2. Analysis of levels LCR-associated transcription factors in HNSCC cell lines

(A) Schematic representation of the workflow. Representative immunoblots of total cellular proteins (25 μg/lane) isolated from HNSCC cells separated on 10% SDS-PAGE were transferred on PVDF membrane. (B) Blots were probed for AP-1 family members. First probed for Fra-1, and then reprobed for Fra-2, c-FOS, FosB, JunB, JunD, c-Jun, and β-actin. (C) Blots were probed for NF-κB family members. First probed for p50/p105, and then reprobed for p52/p100, p65, c-Rel, Rel-B, and β-actin. (D) Blots were probed for pSTAT3(Y705/S727) and STAT3. First probed for pSTAT3 (Y705), pSTAT3 (S727), STAT3, and β-actin. (E) Blots were probed for other inducible transcription factors (GR, PR, FOXA1, and FOXA2). (F) Blots were probed for constitutive transcription factors (SP1, SOX2, CEBPB, TEF1, YY1, and NF-1). The bar graphs represent aggregated normalized band intensities of the immunoblots performed that were grouped into HPV-positive and HPV-negative as per the HPV status the cells. Data plotted as mean band intensity of AP-1 members ± SEM ($n = 3$ for HPV-negative and $n = 2$ for HPV-positive) of the representative experiment out of three independent experiments. $^*p < 0.05$. The numbers next to the western blot represent the molecular weight (kD) of the indicated transcription factors.

HPV-negative HNSCC cells (Figure 2D). High levels of STAT3 were found in all cells except UDSCC2. STAT3 gets phosphorylated at key tyrosine (Y705) and serine (S727) residues in its active form. Analysis of the level of phosphorylated STAT3 at Y705 and S727 showed that STAT3 protein with two types of phosphorylation states were detected in HPV-positive and HPV-negative HNSCC cells. The level of pSTAT3(S727) was characteristically and uniformly high in HPV-negative HNSCC cells ($p < 0.05$). Total STAT3 or pSTAT3(Y705) pools were also elevated, but the differences did not cross the level of significance.

GR and PR

The GR and PR are hormone-dependent transcription factors. GR governs the expression of glucocorticoid-responsive genes, while PR displays cell-dependent transcriptional activity. Immunoblotting revealed a detectable expression of GR and PR in both HPV-positive and HPV-negative HNSCC cells (Figure 2E). The level of GR was nearly uniform in both HPV-negative and HPV-positive HNSCC cells. Whereas, the level of PR was higher in all HPV-negative HNSCC cells ($p < 0.05$).

FOXA1 and FOXA2

FOXA1 (hepatocyte nuclear factor 3 alpha) and FOXA2 (hepatocyte nuclear factor beta) belong to the forkhead box (FOX) family of transcription factors. These factors play a role in promoting gene expression and modifying chromatin structure, facilitating the binding of other factors that regulate transcription. Immunoblotting revealed a detectable expression of FOXA1 and FOXA2 in both HPV-positive and HPV-negative HNSCC cells (Figure 2E). FOXA1 and FOXA2 pools were apparently elevated in 93VU147T.

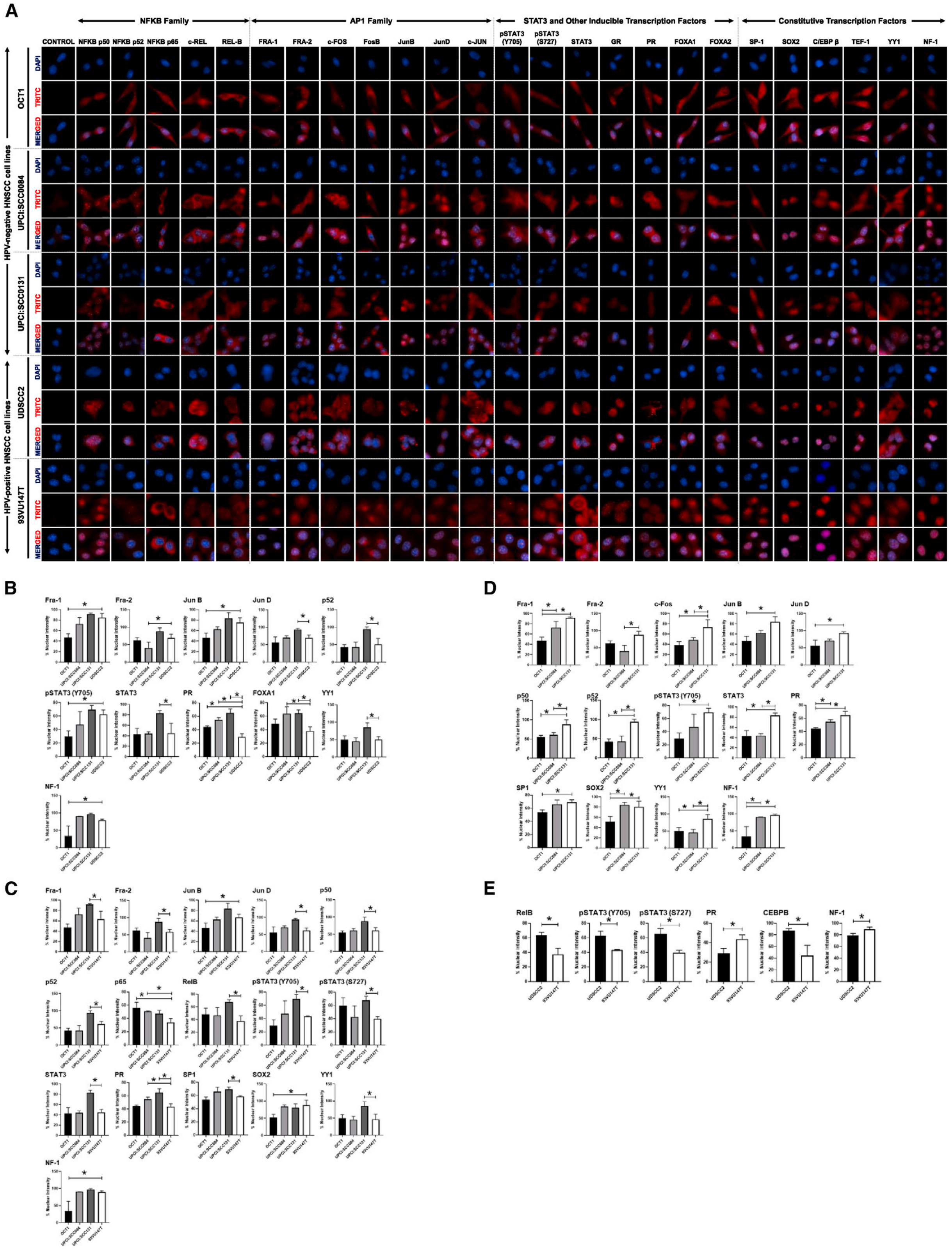

(legend on next page)

Expression analysis of constitutive HPV-related transcription factors in an HNSCC cell line

Immunoblot analysis of SP1, SOX2, CEBPB, TEF-1, YY1, and NF-1 showed variable band intensities of these transcription factors in HNSCC that did not correlate with their HPV status and showed no specific difference in band intensities group wise (Figure 2F). Immunoblotting revealed a detectable expression of SP1 in both HPV-positive and HPV-negative HNSCC cells. SP1 was elevated in HPV-negative HNSCC cells. However, the band intensity of SP1 bands in HPV-negative cells showed a higher normalized mean density as compared with HPV-positive HNSCC cells. The difference was found but it did not cross the level of significance. There were low levels of SOX2 and TEF-1 in these cells, except 93VU147T. Similarly, CEBPB was typically high in UPCI:SCC084. In contrast, YY1 levels were consistently low or undetectable in all HNSCC cell lines except UPCI:SCC084 and UPCI:SCC131. High levels of NF-1 were detectable in only a subset of HPV-negative (UPCI: SCC084 and UPCI: SCC131) and HPV-positive (93VU147T) HNSCC cells.

Analysis of subcellular localization of inducible HPV-related transcription factors in an HNSCC cell line

In the next phase of our investigation, we examined the nuclear involvement of these transcription factors in different HPV-positive and HPV-negative HNSCC cell lines using immunocytochemistry (Figure 3). High-resolution images can be accessed in Figure S1.

AP-1

There was a heterogeneous distribution of signals of different AP-1 members (Figure 3A). However, many members of the AP-1 family showed discrete nuclear positivity, the nuclear positivity ranged from nearly 40% (c-Fos in UPCI:SCC131) to 95% (Fra-1 and Fra-2 in UPCI:SCC131). Analysis of all the AP-1 members tested through immunocytochemistry revealed no quantitative differences in the fraction of signal detected in the nucleus when compared with groups of HPV-positive and HPV-negative HNSCC cells (Figure S2A). We examined HPV-negative cells as a group w.r.t. status of nuclear positivity of respective transcription factors in each HPV-positive HNSCC cell individually (Figures 3B and 3C). As compared with UDSCC2, only OCT1 showed a reduced nuclear positivity for Fra-1 and JunB, whereas only UPCI:SCC084 showed characteristically lower nuclear positivity for Fra-2. In contrast, UPCI:SCC131 showed higher nuclear positivity for JunD. In contrast, when compared with 93VU147T, UPCI:SCC131 showed higher nuclear positivity for Fra-1, Fra-2, and JunD, whereas lower nuclear positivity of JunB was detected in OCT1. All rest of the members showed minor variations in nuclear positivity that were statistically not significant. Within HPV-negative HNSCC cell lines, a significant variation was noted in nuclear positivity of Fra-1, Fra-2, c-Fos, JunB, and JunD (Figure 3D). In contrast, there was no such variation observed in HPV-positive HNSCC cells (Figure S2A).

NF-κB

There was a heterogeneous distribution of signals of different NF-κB members (Figure 3A). However, many members of the NF-κB family showed discrete nuclear positivity; the nuclear positivity ranged from nearly 40% (c-Rel in 93VU147T) to 95% (p52 in UPCI:SCC131). Analysis of all NF-κB members tested through immunocytochemistry revealed no quantitative differences in the fraction of signal detected in the nucleus when compared in groups of HPV-positive and HPV-negative HNSCC cells (Figure S2B). We examined HPV-negative cells as group w.r.t. status of nuclear positivity of respective transcription factors in comparison with different HPV-positive HNSCC cells (Figures 3B and 3C). As compared with UDSCC2, only UPCI:SCC131 showed a higher nuclear positivity for p52. Further, UPCI:SCC131 showed higher nuclear positivity for p50, p52, and RelB as compared with 93VU147T, whereas higher nuclear positivity was detected for p65 in OCT1 and UPCI:SCC084. The remaining members exhibited only minor variations in nuclear positivity, none of which were statistically significant. Within HPV-negative HNSCC cell lines, a significant increase in nuclear positivity was noted for p50 and p52 in UPCI:SCC131 (Figure 3D). However, within HPV-positive HSNCC cells, RelB was typically higher in UDSCC2 ($p < 0.05$) (Figure 3E).

STAT3

A heterogeneous subcellular distribution of STAT3 and its active forms (phosphorylated at Y705 and S727) was observed (Figure 3A). STAT3 showed discrete nuclear positivity, the nuclear positivity ranged from nearly 30% to 80% in HNSCC cells. Analysis of STAT3 and its active form tested through immunocytochemistry revealed no quantitative differences in the fraction of signal detected in the nucleus when compared with groups of HPV-positive and HPV-negative HNSCC cells (Figure S2C). We examined quantitative differences in HPV-negative cells as group w.r.t. status of nuclear positivity of respective pools of STAT3 transcription factors [STAT3, pSTAT3(Y705), and pSTAT3(S727)] in comparison with different HPV-positive HNSCC cells (Figures 3B and 3C). As compared with

Figure 3. Quantitative estimation and analyses of nuclear positivity of LCR-associated transcription factors in HNSCC cell lines

(A) Representative fluorescence photomicrographs showing subcellular localization of LCR-associated transcription factors in HNSCC cell lines. Cells were fixed, stained with respective antibodies and detected with goat anti-rabbit IgG-Alexa-594. Cells were stained for AP-1 family (Fra-1, Fra-2, c-FOS, Fos-B, JunB, JunD, and c-Jun), NF-κB family (for p-50, p52, p65, c-Rel, and Rel-B), pSTAT3 (Y705), pSTAT3 (S727), STAT3, other inducible transcription factors (GR, PR, FOXA1, and FOXA2), and constitutive transcription factors (SP1, SOX2, CEBPB, TEF1, YY1, and NF-1); Red. All cells were counterstained with DAPI; Blue (scale bar, 10 μm). (B) The bar graphs represent the percentage nuclear positivity of transcription factors in HPV-negative HNSCC cells w.r.t. individual HPV-positive HNSCC cells (UDSCC2). (C) The bar graphs represent the percentage nuclear positivity of transcription factors in HPV-negative HNSCC cells w.r.t. individual HPV-positive HNSCC cells (93VU147T). (D) The bar graphs represent the percentage nuclear positivity of AP-1 family members within HPV-negative HNSCC cells. (E) The bar graphs represent the percentage nuclear positivity of AP-1 family members within HPV-positive HNSCC cells (right). Data plotted as mean of percentage nuclear positivity ±SD of the representative experiment out of three independent experiments. *$p < 0.05$. (For high-resolution images, refer Figure S1).

UDSCC2, OCT1 showed lower nuclear positivity for pSTAT3(Y705). In contrast, UPCI:SCC131 showed a higher nuclear positivity for STAT3. Further, UPCI:SCC131 showed higher nuclear positivity for pSTAT3(Y705), pSTAT3(S727), and STAT3 as compared with 93VU147T. Within HPV-negative HNSCC cell lines, a significant variation was noted in nuclear positivity of pSTAT3(Y705) and total STAT3 (Figure 3D). However, within HPV-positive HSNCC cells, pSTAT3(Y705) and pSTAT3(S727) were higher in UDSCC2 ($p < 0.05$) (Figure 3E).

Other inducible transcription factors

GR, PR, FOXA1, and FOXA2 showed discrete nuclear positivity with nuclear positivity ranging from nearly 50% (FOXA1 in OCT1) to 80% (GR in UPCI:SCC131) in HNSCC cells (Figure 3A). Analysis of inducible transcription factors tested through immunocytochemistry revealed no quantitative differences in the fraction of signal detected in the nucleus when compared in groups of HPV-positive and HPV-negative HNSCC cells (Figure S2D). We examined HPV-negative cells as group w.r.t. status of nuclear positivity of respective transcription factors in comparison to different HPV-positive HNSCC cells (Figures 3B and 3C). As compared with UDSCC2, UPCI:SCC084 and UPCI:SCC131 showed higher nuclear positivity for PR and FOXA1. OCT1 also showed a higher nuclear positivity for PR. Further, UPCI:SCC084 and UPCI:SCC131 showed higher nuclear positivity for PR as compared with 93VU147T. The remaining showed minor variations in nuclear positivity that were not statistically significant. Within HPV-negative HNSCC cell lines, a significant variation was noted in nuclear positivity of PR (Figure 3D). However, within HPV-positive HSNCC cells, PR was higher in 93VU147T ($p < 0.05$) (Figure 3E).

Analysis of subcellular localization of constitutively active HPV-related transcription factors in the HNSCC cell line

A heterogeneous distribution of nuclear positivity of different constitutive transcription factors SP1, SOX2, CEBPB, TEF-1, YY1, and NF-1 was observed (Figure 3A). A discrete nuclear positivity ranged from nearly 30% (NF-1 in OCT1) to 100% (SOX2 in UPCI:SCC131, as well as NF-1 in 93VU147T). Analysis of all the AP-1 members tested through immunocytochemistry revealed no quantitative differences in the fraction of signal detected in the nucleus when compared with groups of HPV-positive and HPV-negative HNSCC cells (Figure S2E). We examined HPV-negative cells as group w.r.t. status of nuclear positivity of respective transcription factors in comparison with different HPV-positive HNSCC cells (Figures 3B and 3C). As compared with UDSCC2, only UPCI:SCC131 showed a higher nuclear positivity for YY1, whereas only OCT1 showed characteristically lower nuclear positivity for NF-1. Further, UPCI:SCC131 showed higher nuclear positivity for SP1 and YY1 as compared with 93VU147T, whereas lower nuclear positivity was detected in OCT1 for SOX2 and NF-1. The rest of the members showed minor variations in nuclear positivity that were statistically not significant. Within HPV-negative HNSCC cell lines, a significant variation was noted in the nuclear positivity of SP1, SOX2, YY1, and NF-1 (Figure 3D), wherein the factors were significantly expressed low in OCT1. However, within HPV-positive HSNCC cells, nuclear positivity for CEBPB and NF-1 was higher and lower in UDSCC2, respectively ($p < 0.05$) (Figure 3E).

To characterize the expression profiles of LCR-specific transcription factors in HNSCC patients

Expression analysis of LCR-associated transcription factors in HNSCC patients

To check the clinical relevance of inducible and constitutive transcription factors transcripts in HNSCC patients, we examined the HNSCC transcriptome dataset available at The Cancer Genome Atlas (TCGA) for expression and survival analysis in corresponding HNSCC patients (Figure 4A). In the AP-1 family, the expression of Fra-1 transcripts was low whereas JunB and JunD transcripts were comparatively high in HPV-positive HNSCC tumors (Figure 4B). However, transcripts for the other AP-1 members—Fra-2, c-Fos, FosB, and c-JUN—displayed variability in both groups, though the differences were not statistically significant. In the NF-κB family, the expression of p50, p52, and RelB transcripts was high in HPV-positive HNSCC tumors (Figure 4C). Whereas, transcripts for only RelA showed no variation in both tumors. The data for c-Rel were unavailable. The expression of STAT3 and FOXA1 transcripts was also high in HPV-positive HNSCC tumors in comparison with HPV-negative tumors (Figure 4D). Among transcripts of other constitutive transcription factors, GR, PR, and FOXA2 were similar in HPV-positive and HPV-negative tumors. Among constitutive transcription factors, SOX2, SP1, and NF-1 transcripts were high, while CEBPB transcripts were low in HPV-positive HNSCC tumors (Figure 4E). Transcripts of TEF-1 in HPV-positive tumors were low, but the difference did not cross the level of significance, whereas the transcript levels of YY1 in both HNSCC tumors were similar.

Prognostic association analysis of LCR-associated transcription factors

The expression levels of various transcription factors were associated with survival outcomes in HNSCC, with notable differences based on HPV status (Figure 5). In overall cohort, the high expression of the AP-1 family (Fra-1, Fra-2, FosB, JunB, and c-JUN), NF-κB family (RelA), PR, and FOXA1 were associated with poor survival. In contrast, high expression of NF-κB family (p50, p52, and RelB), and SP1 were associated with better survival in overall cohort. Based on HPV-positivity by p16 staining, high expression of AP-1 family (Fra-1), NF-κB family (p50, p52), and GR in HPV-negative cohort were associated with poor survival. On the contrary, increased expression of FOXA2 and SP1 were associated with better survival outcomes. In the HPV-positive cohort, low expression of AP-1 family (JunB), NF-κB family (p50, p52, RelA, and RelB), STAT3, and CEBPB were associated with poor survival, while low expression of FOXA1 was associated with better survival.

To evaluate HPV16 LCR reporter activity in HPV-positive and HPV-negative HNSCC cell lines

Structural analysis of HPV16 LCR plasmids

To assess LCR reporter activity, plasmids were verified for the presence of the LCR region (Figure S3). The vector pGL4-LCR-HPV16

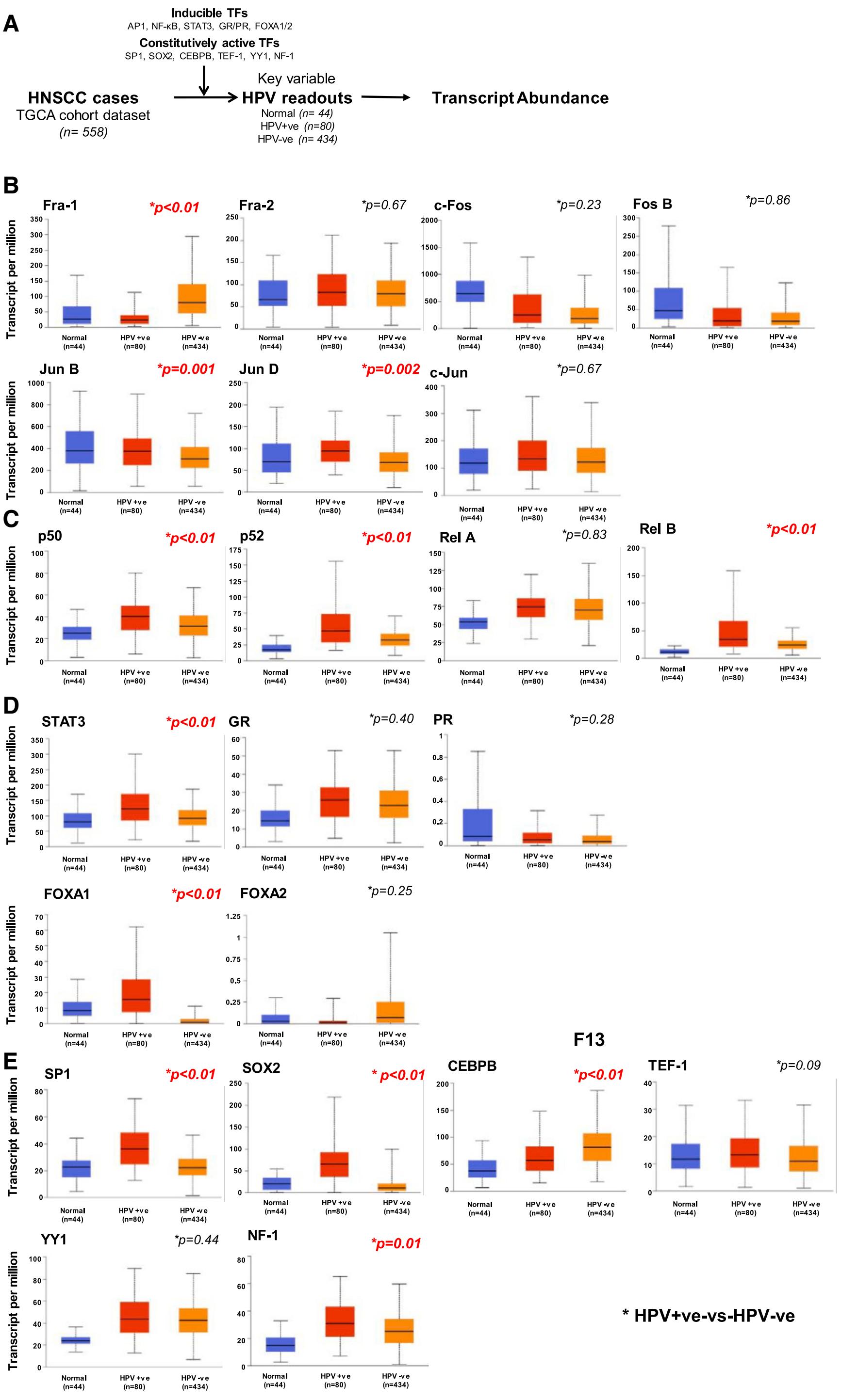

Figure 4. Status of LCR-associated transcription factors in TCGA HNSCC cohort

(A) Schematic representation of the workflow. (B–E) Box-whisker plots representation of the validated inducible transcription factors. Blue, orange, and yellow bars represent data from Normal, HPV-positive, and HPV-negative cohorts, respectively. Expression of the LCR-specific transcription factors by the application of UALCAN analysis and visualization platform based on TCGA. The data were grouped based on HPV status [negative (N = 434) and positive (N = 80)], Welch's T test applied to estimate the significance of differences in expression levels between normal and tumor subgroups based on HPV status and the adjusted $\beta\beta$ values were generated by the online platform with a p value of <0.05 was considered significant. *(* HPV-positive v/s HPV-negative).*

(nt 7000-100), containing nucleotides 7,000 to 100 of the HPV16 genome (RefSeq: NC_001526.1), was inserted into the SacI/BglII sites of pGL4.20, along with pGL4-LCR-HPV16 (nt 7000-85), which contains nucleotides 7,000 to 85 of the same region. In pGL4-LCR-HPV16 (nt 7000-85), nucleotides 4–18 of the E6 gene were missing. Linearized plasmid digested with *SacI* confirmed the size of plasmids. HPV16-LCR specific PCR performed on LCR confirmed the presence of LCR region in both LCR plasmids.

Evaluation of HPV16 LCR reporter activity in HNSCC cells

For a more comprehensive understanding of transcriptional control of the HPV16-LCR by the transcriptional milieu of HPV-negative and HPV-positive HNSCC cell lines, relative light units of plasmids were analyzed (Figure 6A). These constructs were transfected in different HNSCC cell lines and the LCR activity was detected in both HPV-positive and HPV-negative HNSCC cells in comparison to empty vector pGL4 except in UPCI:SCC084 (Figure 6B). pGL4-LCR-HPV16 (nt 7000-100)-luciferase consistently showed better LCR activity; however, the difference crossed the level of significance only in UDSCC2. Both the constructs showed LCR activity in HPV-positive HNSCC cells as expected. Interestingly, LCR activity was also detected in HPV-negative HNSCC cells. The phenomenon was reproducible as two independent constructs showed similar results. In a cumulative analysis of both LCR, plasmids showed similar LCR activity in HPV-positive and HPV-negative HNSCC cell groups (Figure 6C). Further, the LCR activity of HPV-negative HNSCC cells was evaluated in comparison with HPV-positive cells for both constructs (Figure 6D). UPCI:SCC131 was comparable with UDSCC2, while OCT1 and UPCI:SCC084 exhibited reduced LCR activity w.r.t. UDSCC2, whereas the LCR activity of UPCI:SCC131 and OCT1

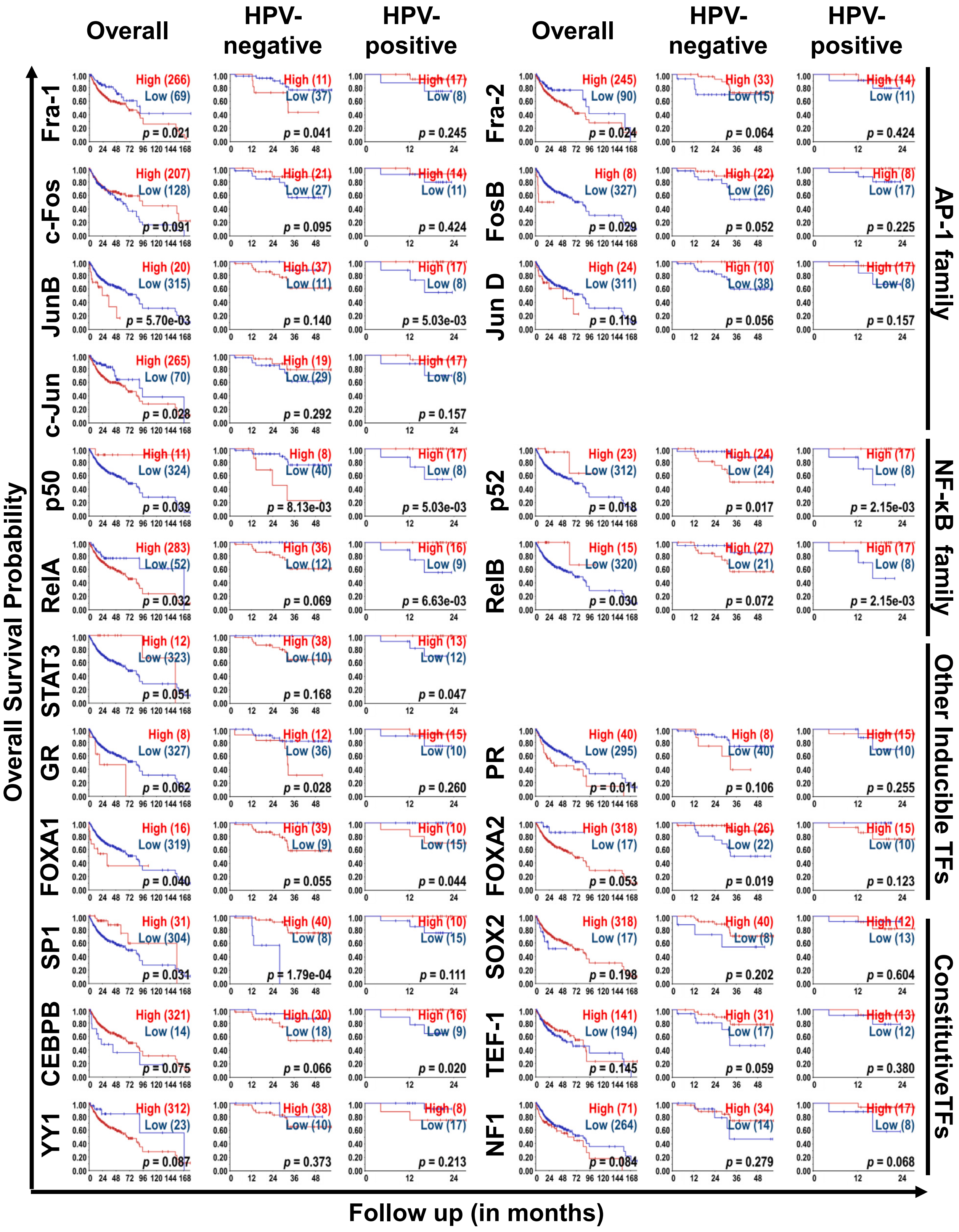

(legend on next page)

was high in comparison with 93VU147T and UPCI:SCC084 exhibited similar LCR activity w.r.t. 93VU147T.

Assessment of HPV16 LCR reporter activity in HNSCC cells for tissue of origin

The LCR activity pattern exhibited inverse correlation with the aggressiveness of their tumor of origin (Figure 6E). Despite their common origin (oral cavity) of HPV-negative HNSCC cells, UPCI:SCC131 and UPCI:SCC084 displayed contrasting LCR activities. Notably, UPCI:SCC131 was from new primary well-differentiated tumors that had high LCR activity lesions, and UPCI:SCC0084 was from a recurrent aggressive tumor and had low LCR activity. Similarly, HPV-positive cells were evaluated according to their tissue of origin (Figure 6F). UDSCC2, derived from the hypopharyngeal region, exhibited higher HPV16 LCR activity compared with 93VU147T, which originated from the oral cavity.

Analysis to reveal correlation between protein expression, nuclear positivity and LCR activity

Multiple linear regression analysis was conducted to determine whether expression activity as observed by immunoblotting or nuclear positivity as observed by immunofluorescence could predict the LCR activity of HNSCC cell lines or not (Table 1). When the findings of multiple linear regression analysis were examined, it revealed that out of entire set of 25 LCR transcription factors, only pSTAT3(S727)s' expression activity as well as nuclear positivity predicted LCR activity significantly. In this regard, a comparable LCR activity was shown between UPCI:SCC131 and UDSCC2 (Figure 6B). The same observation was observed in cases of nuclear positivity, wherein no significant difference was observed between UPCI:SCC131 and UDSCC2, and pSTAT3(S727)'s nuclear positivity of UPCI:SCC131 was significantly different with respect to 93VU147T (Figure S2C). Additionally, the significant difference in expression levels of this transcription factor between HPV-positive and HPV-negative groups in Figure 2 is attributed to the cumulative expression levels of the cell lines within each respective group.

DISCUSSION

The present study investigated the transcriptional milieu of well-characterized HPV-negative (OCT1, UPCI:SCC084, and UPCI:SCC131) and HPV-positive (UDSCC2 and 93VU147T) HNSCC cell lines. A literature review of LCR of the HPV16 genome revealed the presence of potential binding motifs for a set of seven inducible transcription factors (AP-1 family, NF-κB family, STAT3, GR, PR, FOXA1, and FOXA2) and six constitutively active transcription factors (SP1, SOX2, CEBPB, TEF-1, YY1, and NF-1). The expression and localization of these transcription factors in HNSCC cell lines were assessed by immunoblotting and immunocytochemistry, respectively. Upon assessment of LCR activities in HPV-positive and HPV-negative cell lines using HPV16 LCR luciferase constructs, it was found that UPCI:SCC131 (HPV-negative) showed comparable HPV16 LCR activity with the HPV-positive HNSCC cell line. Further, validation of HPV16 LCR-specific transcription factors using TCGA-based UALCAN software indicated upregulation of six transcription factors, namely, NF-κB p50, NF-κB p52, RelB, FOXA1/2, SOX2, and NF-1, in the HPV-positive cohort compared with normal and the HPV-negative cohort. In addition, Kaplan-Meier analysis unveiled strong correlation with overall survival in HNSCC patients.

Building on previous research conducted by our laboratory and other groups, which delineated the involvement of transcription factors in regulating the early phase of HPV infection in host cells,[35,39,40] our present investigation delves deeper into the transcriptional milieu of both HPV-negative and HPV-positive HNSCC cell lines, specifically examining their HPV16 LCR activity, which was further validated by TCGA cohort. Previously, we manually analyzed the HPV16 reference genome to look into the binding sites of various transcription factors present on HPV16 LCR.[6] Collectively, our results and other reports point to an involvement of a set of seven inducible and six constitutively active transcription factors in the transcriptional regulation of HPV oncoproteins via putative binding sites, as predicted on HPV16 LCR. These transcription factors, known for their binding affinity to the HPV16 LCR, have been investigated in cervical cancer.[41,42] Studies have shown the importance of c-Jun/AP-1 in driving HPV transcription in HPV-positive cervical cancer[43] and STAT3 in driving replication in HPV-containing keratinocytes.[44] These previous observations may be in contrast with our study in terms of HPV LCR binding, but remain highly relevant considering the significance of STAT3 and NF-κB/AP-1 in HN carcinogenesis.[45,46] Similarly, YY1 has been shown to be crucial for HPV transcriptional regulation via chromatin looping, as shown by transfection and mutation of pGEMII-HPV18 in primary human keratinocytes.[47] However, similar transcriptional milieu analysis data are still lacking in the case of HNSCC.

While dissecting the expression of AP1 family in HNSCC cell lines using immunoblotting, Fra-1, JunD, and c-Jun exhibited distinct HPV-positivity-specific expression. Upregulated expression of Fra-2 and JunB was observed in all HNSCC cell lines, irrespective of HPV positivity. Interestingly, Fra-2, c-Fos, and FosB in UPCI:SCC131 showed similar expression patterns to that of the HPV-positive HNSCC cell lines. In the same line, similar results were observed in HNSCC for differential expression of JunB, c-Fos, Fra-1, and JunD.[48,49] Our observations, supported by previous studies,[8,40,50,51] revealed the absence of c-Jun in the AP1

Figure 5. Kaplan-Meier survival analysis of LCR-associated transcription factors and their prognostic association

The survival curves comparing the HNSCC patient data in overall (N = 335), HPV-negative (N = 48), and HPV-positive (N = 25) cohorts displaying high (red) and low (blue) expression of AP-1 family (Fra-1, Fra-2, c-FOS, Fos-B, JunB, JunD, and c-Jun), NF-κB family (for p-50, p52, p65, c-Rel, and Rel-B), pSTAT3 (Y705), pSTAT3 (S727), STAT3, other inducible transcription factors (GR, PR, FOXA1, and FOXA2,) and constitutive transcription factors (SP1, SOX2, CEBPB, TEF1, YY1, and NF-1) ($p < 0.05$).

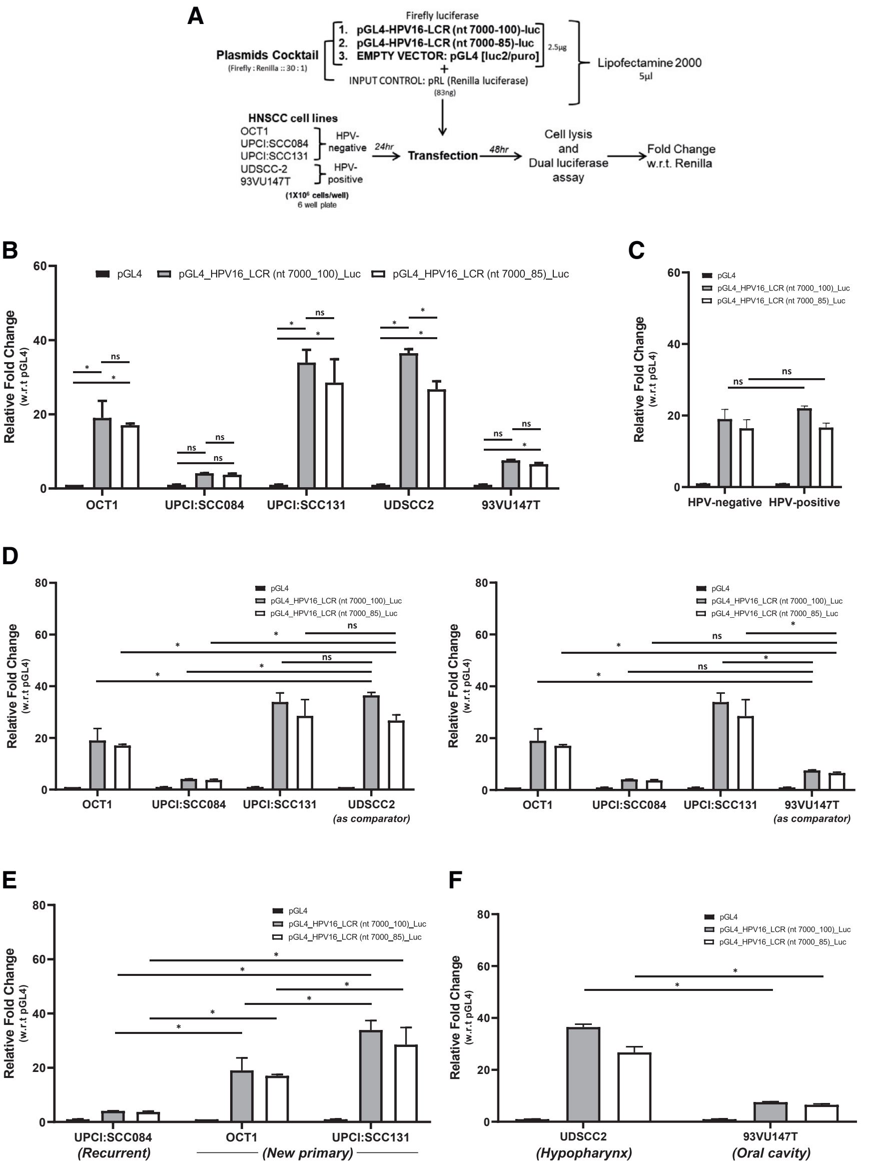

Figure 6. HPV16-LCR construct specific LCR activity differences in HPV-negative and HPV-positive HNSCC cells and cell type-specific and HNSCC region-specific differences of HPV16-LCR activities

(A) Schematic representation of the workflow. (B) Luciferase reporter assays were conducted to explore the inherent promoter activities of HPV16 LCRs in both HPV-negative and HPV-positive HNSCC cells. The cells were transfected with 25 ng of an empty reporter plasmid (pGL4.20), or HPV16-LCR reporter plasmids along with the input control vector pRL. After 48 h, cell lysates were prepared and analyzed for luciferase activities. The luciferase activities are presented relative to those of pGL4.20. The data represent the mean of three independent experiments, each performed in duplicate ± SD. (C) The bar graphs represent aggregated relative fold change of HPV16-LCR constructs [top: pGL4-LCR-HPV16 (nt 7000-100) and bottom: pGL4-LCR-HPV16 (nt 7000-100)] w.r.t. pGL4 of the luciferase activity, that were grouped into HPV-positive and HPV-negative as per the HPV status of the cells. Data plotted as mean of relative fold change HPV16-LCR constructs w.r.t. pGL4 ± SEM ($n = 3$ for HPV-negative and $n = 2$ for HPV-positive) of the representative experiment out of three independent experiments *$p < 0.05$. (D) The bar graphs represent the relative fold change of HPV16-LCR constructs [left: pGL4-LCR-HPV16 (nt 7000-100) and right: pGL4-LCR-HPV16 (nt 7000-100)] w.r.t. pGL4 of the luciferase activity, in HPV-negative HNSCC cells w.r.t. HPV-positive HNSCC cells (top: UDSCC2 and bottom: 93VU147T). Data plotted as mean of relative fold change HPV16-LCR constructs w.r.t. pGL4 ± SD of the representative experiment out of three independent experiments. *$p < 0.05$. (E) The bar graphs represent the relative fold change of HPV16-LCR constructs [left: pGL4-LCR-HPV16 (nt 7000-100) and right: pGL4-LCR-HPV16 (nt 7000-100)] w.r.t. pGL4 of the luciferase activity, in OSCC new primary and recurrent cells with HPV-negative status. Data plotted as mean of relative fold change HPV16-LCR constructs w.r.t. pGL4 ± SD of the representative experiment out of three independent experiments *$p < 0.05$. (F) The bar graphs represent the relative fold change of HPV16-LCR constructs [left: pGL4-LCR-HPV16 (nt 7000-100) and right: pGL4-LCR-HPV16 (nt 7000-100)] w.r.t. pGL4 of the luciferase activity, in HPV-positive HNSCC cells of the hypopharynx and oral cavity region. Data plotted as mean of relative fold change HPV16-LCR constructs w.r.t. pGL4 ± SD of the representative experiment out of three independent experiments. *$p < 0.05$.

complex of HPV-positive tumors. Notably, irrespective of HPV status, c-Jun demonstrated a poor prognosis in OSCC.[52]

Further, the expression of the NF-κB family was evaluated in HNSCC cell lines using immunoblotting, revealing that only p65 displayed distinct HPV-positivity-specific expression. Conversely, upregulated expression of p50 and p52 was evident in all HNSCC cell lines, regardless of HPV positivity. Intriguingly, UPCI:SCC131 exhibited a similar expression pattern for p50 and p52, as observed in HPV-positive HNSCC cell lines. Constitutive activation of NF-κB was noted in all HNSCC cell lines, a finding consistent with previous research.[36,38,53,54] Notably, UPCI:SCC084, derived from a recurrent SCC, demonstrated constitutively active NF-κB, as demonstrated earlier.[55]

Moreover, while dissecting the expression of HPV16 LCR-specific inducible transcription factors in HNSCC cell lines using immunoblotting, pSTAT3(S727) and pSTAT3(Y705) exhibited distinct HPV-positivity-specific expression. Conversely, downregulated expression of pSTAT3-(S727), STAT3, GR, and PR was observed in all HNSCC cell lines irrespective of HPV positivity. As previously shown, the expression of active STAT3 correlated with HPV-negative

Table 1. Association of LCR activity (dependent variable) with expression and nuclear positivity (independent variables) of respective transcription factors using multiple regression analysis

	Expression Analysis				Nuclear positivity				Multiple R	Adjusted R^2	Significance *F* value
Transcription factor	r^2	S.E.	*t*	*p* value	r^2	SE	*t*	*p* value			
NF-KB P50	−1.29	0.64	−1.99	0.18	0.68	0.41	1.65	0.24	0.86	0.48	0.26
NF-KB P52	−3.56	2.04	−1.74	0.22	0.36	0.27	1.33	0.31	0.83	0.38	0.30
NF-KB P65	−5.67	13.48	−0.42	0.71	0.58	1.11	0.52	0.65	0.46	−0.58	0.79
CREL	−3.65	2.98	−1.22	0.34	1.11	0.47	2.36	0.14	0.92	0.70	0.15
RELB	−0.62	1.14	−0.54	0.64	1.02	0.32	3.15	0.09	0.92	0.71	0.14
FRA-1	−9.37	10.13	−0.92	0.45	0.56	0.41	1.37	0.30	0.73	0.07	0.46
FRA-2	1.10	8.60	0.13	0.91	0.75	0.38	1.97	0.19	0.85	0.45	0.28
C-FOS	−12.30	10.68	−1.15	0.37	0.09	0.64	0.14	0.90	0.71	0.036	0.48
FOSB	−19.12	12.46	−1.53	0.26	0.66	0.83	0.79	0.51	0.74	0.09	0.45
JUNB	−75.42	32.72	−2.30	0.15	2.14	0.75	2.86	0.10	0.90	0.62	0.19
JUND	−11.77	12.90	−0.91	0.46	0.86	0.64	1.34	0.31	0.69	−0.04	0.52
CJUN	−15.40	8.24	−1.87	0.20	1.90	1.19	1.60	0.25	0.81	0.33	0.33
pSTAT3(Y705)	−0.05	27.09	−0.001	0.998	0.61	0.49	1.23	0.34	0.66	−0.12	0.56
pSTAT3(S727)	−13.29	2.05	−6.45	0.02	1.09	0.05	20.24	0.002	0.99	0.99	0.004
STAT3	−23.50	28.06	−0.84	0.49	0.47	0.44	1.07	0.39	0.68	−0.08	0.54
SOX2	−2.89	30.01	−0.09	0.93	−0.21	0.71	−0.30	0.79	0.21	−0.90	0.95
SP1	−12.92	7.57	−1.70	0.23	1.12	1.20	0.93	0.45	0.77	0.18	0.40
GR	9.18	27.13	0.34	0.76	1.25	0.64	1.94	0.19	0.80	0.30	0.34
PR	−8.04	28.34	−0.28	0.80	−0.07	0.83	−0.09	0.93	0.25	−0.87	0.93
CEBPB	−39.34	44.85	−0.88	0.47	0.48	0.47	1.03	0.41	0.70	−0.004	0.50
TEF-1	24.96	44.72	0.56	0.63	3.92	2.36	1.65	0.23	0.76	0.16	0.42
YY-1	−48.36	34.61	−1.39	0.30	0.43	0.35	1.21	0.34	0.82	0.36	0.31
NF-1	−16.68	7.90	−2.10	0.17	0.50	0.33	1.51	0.27	0.83	0.38	0.31
FOXA1	−14.40	19.41	−0.74	0.53	−0.52	0.59	−0.87	0.47	0.59	−0.29	0.64
FOXA2	70.20	26.86	2.61	0.12	4.48	1.36	3.29	0.08	0.92	0.70	0.15

r^2: Regression coefficient, t: Student's T test statistic value. Only pSTAT3(S727) showed direct association of LCR activity with its expression and nuclear positivity.

HNSCC cell lines.[35,40] Despite some studies reporting elevated STAT3 in HPV-positive HNSCC,[56,57] similar to HPV-induced cervical carcinogenesis,[58,59] the reasons behind these contrasting observations remain unclear. Notably, high PR expression in HPV-negative OSCC tumors has been associated with locoregional recurrence-free and poor disease-specific survival.[60] However, no specific study examines the relationship between GR and HNSCC.

While scrutinizing the constitutively active transcription factors specific to HPV16 LCR in HNSCC cell lines through immunoblotting, a distinct downregulation in SOX2 expression was evident in HPV-positive HNSCC cell lines, consistent with findings from another research group.[61] Downregulated expression of CEBPB and TEF-1 was observed in all HNSCC cell lines, irrespective of HPV positivity. Interestingly, SP1 and YY1 in UPCI:SCC131 showed a similar expression pattern to that of HPV-positive HNSCC cell lines. It is worth noting that there are no HPV-specific studies available for SP1 in HNSCC. Conversely, elevated expression of YY1 impedes the transcriptional activation of HPV in pre-neoplastic tissues, resulting in a fate resembling that of HPV-negative tumors.[62–64]

Additionally, to examine the cellular localization of these transcription factors, immunocytochemistry was conducted, revealing cell-line-specific localization for the majority of factors. Notably, c-Fos, c-Rel, GR, FOXA1, FOXA2, and YY1 exhibited similar cellular localization in UPCI:SCC084, UPCI:SCC131, and UDSCC2. Similarly, pSTAT3 (Y705), pSTAT3 (S727), and NF-1 displayed comparable localization in UPCI:SCC084, UPCI:SCC131, and 93VU147T. SP1 exhibited similar expression patterns in UPCI:SCC131 and 93VU147T. The expression of TEF-1 in UPCI:SCC084 correlated with UDSCC2, while UPCI:SCC131 correlated with 93VU147T. Furthermore, the expression of CEBPB in UPCI:SCC131 correlated with HPV-positive HNSCC cell lines. These transcription factors play a crucial role in binding to HPV16 LCR, facilitating the

transcriptional activation and regulation of functional HPV16 oncoproteins (E6/E7), which are essential for the establishment of carcinogenesis. Consequently, no specific correlation in the localization of various transcription factors was observed in HNSCC cell lines.

Further validation of *in vitro* data using TCGA database (N = 554; normal, N = 44; HPV-positive, N = 80; HPV-negative, N = 434) revealed the upregulation of six key transcription factors: p50, p52, RelB, FOXA1, SOX2, and NF-1 in the HPV-positive cohort in comparison with both normal and the HPV-negative cohorts. Furthermore, to understand the correlation between the expression levels of LCR-associated transcription factors and the survival outcomes in HNSCC patients, Kaplan-Meier survival analysis of the R2 database was used. Of the two available HPV-classification tracks in R2 database for survival analyses, p16 immunohistochemistry and *in situ* hybridization (ISH), p16 staining was selected. This decision was driven by the limited availability of ISH data, which was restricted to a small subset of the dataset, thereby reducing its statistical strength and overall reliability. Moreover, a 98% positive correlation has been reported between these two techniques, thereby highlighting p16 staining as an equally effective surrogate marker for HPV detection in HNSCC.[65] Our prognostic findings align with prior reports highlighting the association of high Fra-1 expression with poor prognosis and shorter survival across *in vitro*, *in vivo*, and clinical studies.[52,66,67] c-Fos showed context-dependent effects, predicting poor outcomes in most OSCC studies[68–70] but better prognosis in specific nasopharyngeal carcinoma (NPC) cohorts.[71,72] Elevated expression of Jun family members (JunB, c-Jun, and JunD) generally correlated with poor outcomes.[52,70,73–75] NF-κB subunits (P50, P52, RelA, P65, and RelB) were mostly linked to poor prognosis, although high P65 levels indicated improved survival in HPV-positive cases.[38,76–78] Although high expression of STAT3[38,76–78] was associated with better survival, as observed in the present study, in contrast with our present finding of FOXA1, FOXA1[79,80] overexpression was associated with better outcomes in overall cohort. In contrast with our findings, high SP1,[81] CEBPB,[82] and YY1[83] levels were linked to a poor prognosis, especially in HPV-negative or advanced cancers. Although most studies primarily focused on overall survival outcomes, a few reports explored HPV-associated survival outcomes in connection with LCR-specific transcription factors. For instance, elevated levels of Fra-2 in HPV-negative tongue SCC were linked to aggressive tumor phenotypes and poorer prognosis.[8] Similarly, increased expression of SOX2 enhanced tumorigenicity in HPV-negative OSCC patients.[61] However, these observations were not reflected in our study. In contrast, our results aligned with findings on RelA, where its involvement in HPV-positive HNSCC patients was associated with improved differentiation and better prognosis.[38] Nevertheless, these transcription factors related with HPV-infection showed strong correlation with HNSCC patient survival and may play an independent role in carcinogenesis apart from regulating the HPV oncogene expression.

Structural analysis of LCR-specific plasmid constructs verified the existence of the HPV16-LCR region in both pGL4-LCR-HPV16 (nt 7000-100) and pGL4-LCR-HPV16 (nt 7000-85) plasmids.[84] Currently, there is no study that show LCR activity in HNSCC cells with respect to HPV; however, LCR constructs have been widely evaluated in cervical cancer cell lines.[85–87] Apart from studies based on HPV16 LCR constructs, LCR activity has also been assessed in other HPV subtypes.[88–92] To assess HPV16 LCR activity, HPV16 LCR constructs were transfected in HPV-negative HNSCC cell lines. These cell lines showed a distinct pattern of transcription factors. Interestingly, LCR activity was detected in HPV-negative HNSCC cells.

None of the transcription factors showed any characteristic difference in HNSCC cell lines with respect to their HPV status. Incidentally, the protein level profile of transcription factors as well as nuclear positivity in UPCI:SCC131 (HPV-negative) and UDSCC2 (HPV-positive) matched closely. These two cell types also activated HPV16 LCR to a comparable level. Notably, the nuclear positivity of the AP-1 family was alike in UPCI:SCC131 and UDSCC2, except for JunD. Conversely, Fra-1, Fra-2, and JunD demonstrated significant variations compared with 93VU147T. Within the NF-κB family, 93VU147T displayed uniquely elevated c-Rel levels. UPCI:SCC131 exhibited comparable nuclear positivity to UDSCC2 for all NF-κB members, except p52. The nuclear positivity of RelB showed similarities between HPV-positive HNSCC cells, with UPCI:SCC131 and UDSCC2 displaying analogous nuclear localization. Total STAT3 nuclear localization was notably high in UPCI:SCC131 among all tested HNSCC cells. However, the nuclear localization of phosphorylated forms of STAT3 in UPCI:SCC131 was similar to UDSCC2 and higher compared with 93VU147T. No correlation was found for the nuclear positivity of GR, PR, FOXA1, and FOXA2 in UPCI:SCC131 and UDSCC2. The nuclear positivity for SP1 in UPCI:SCC131 mirrored that of UDSCC2 but differed from 93VU147T, suggesting a possible role of SP1 in LCR transcription regulation. Other constitutive transcription factors did not exhibit such correlations. In summary, members of AP-1, NF-κB, STAT3, and SP1 are likely to play crucial roles in regulating HPV16 transcription. To date, very few studies have shown role of transcription factors in regulation of LCR activity.

In another analysis, LCR activity patterns correlated with the aggressiveness of their tumor of origin, e.g., OCT1, UPCI:SCC131, and UPCI:SCC084, despite having the same origin (oral cavity), displayed contrasting LCR activities, where OCT1 and UPCI:SCC131 were from new primary well-differentiated lesions and UPCI:SCC084 was from a recurrent aggressive tumor. LCR activity in both new primary oral cavity origin showed higher LCR activity in comparison with the recurrent one. Similarly, HPV-positive cells also showed a variable LCR activity. The data suggest that HPV16-LCR can be activated by the cell lacking HPV16 infection and they display similar transcription factor profile as HPV-positive HNSCC.

Overall, the present study provides a collective assessment of transcription factors possessing putative binding sites on HPV16 LCR that could give mechanistic insights into role of transcriptional milieu in establishment of HPV-associated HNSCC. The expression of NF-κB p50, NF-κB p52, SP1, and YY1 in UPCI:SCC131 was found to be similar to that of HPV-positive cell line that implicates a close link of

these transcription factors to HPV integration into its host genome. Fra-1, SP1, GR, and FOXA2, collectively form clinically useful set of biomarkers for prognostication and effective management of HPV-positive vs. HPV-negative HNSCC. Our study underscores the pivotal role of pSTAT3(S727) in predicting LCR activity within the intricate network of transcription factors. By unraveling its regulatory mechanisms, we pave the way for deeper insights into cellular processes and potential clinical applications in disease management. Therefore, our results demonstrated functional downstream implications of HPV-negative transcriptional milieu favoring the establishment of HPV in their genome and could be an indicator of better prognosis in HNSCC.

MATERIALS AND METHODS

Materials

Cell lines

Human HNSCC cell lines used in the present study along with their key characteristics and HPV status are listed in Table S3. HPV-negative HNSCC cell lines, OCT1 was a kind gift from Dr. Anant Narayan Bhatt, INMAS, DRDO, India, and UPCI:SCC084, UPCI:SCC131 were kind gifts from Dr. Susanne M. Gollin, University of Pittsburgh. HPV-positive HNSCC cell lines UDSCC2 and 93VU147T were kind gifts from Dr. Kathrin Scheckenbach, University of Dusseldorf, Germany, and Dr. Steenbergen, Vrije Universiteit, Netherlands, respectively.

Primers

Primers used for genomic and RT-PCR, their amplicon size, and the annealing temperatures for characterization of HNSCC cell lines are listed in Table S4. High-performance liquid chromatography-purified primers used for genomic and RT-PCR were custom synthesized and procured either from Microsynth, Eurogentec, or Eurofins Scientific.

Cancer transcriptomic data

In this study, transcript analysis was performed on the cancer transcriptome data, publicly made available by the TCGA. A total of 558 datasets of primary HNSCC tumor (N = 514) and normal HN tissues (N = 44) were analyzed using the online tool UALCAN (http://ualcan.path.uab.edu) hosted by the University of Alabama at Birmingham Cancer.[93] The categorization of samples was based on HPV readcounts. HPV status was assessed at the Broad Institute by DNA sequencing and PathSeq algorithm.[94] Specifically, a total of 80 datasets showed presence of HPV transcripts in HNSCC tissues, whereas 434 were confirmed negative for HPV transcripts. HPV status was not available for six samples; hence, they were excluded from the analysis. For Kaplan-Meier survival analyses, another HNSCC dataset “TCGA: Mixed Head and Neck (2022-v32)-tcga-548-tpm-gencode36” (N = 548) available on R2 Genomics and Visualization Platform (http://r2platform.com) was used.

Antibodies

Various antibodies used in the detection of oncoproteins and transcription factors in HNSCC cells via immunoblotting and immunocytochemistry experiments are listed in Table S5. All antibodies were procured from Santa Cruz Biotechnology Inc., BD Biosciences, or Cloud Clone Corporation.

Plasmids

Plasmids used in the present study and their key characteristics are listed in Table S6. The plasmids pGL4-HPV16-LCR (nt 7000-100)-luciferase (*p5193*) harboring HPV16 LCR region from *nt* 7000-100, whereas pGL4-HPV16-LCR (nt 7000-85)-luciferase (*p6239*) harboring HPV16 LCR region from *nt* 7000-85, empty vector pGL4.20[luc2/Puro] and Renilla luciferase (Rluc) used as input control reporter vector with HSV-thymidine kinase promoter were kind gifts from Prof. Ashok Kumar, University of Houston.

All-important reagents media and commercial kits used in the present are listed in Table S7. All reagents used were of analytical or molecular biology grade and procured from M/s Sigma unless specified.

Cell culture and characterization

Different HNSCC cell lines were maintained in their respective medium (Table S3) supplemented with 10% heat-inactivated fetal bovine serum and 1× antibiotic antimycotic solution. All cells were grown in a humidified incubator at 37°C with 5% CO_2. All cells were periodically checked for their morphology (cobblestone: characteristics of SCC) (Figure S4A), growth of culture by calculating cell population doubling time (Figures S4B and S4C).

Cell population doubling

HNSCC (5×10^5) cells/well were seeded in a six-well plate with 2 mL of the respective medium. Viable cell count was taken using the Trypan blue dye exclusion method at different time points including 48 h. A Neubauer counting chamber (Rohem) was used for cell counting. The cell doubling time was calculated at 48hr using the formula: T*ln2/ln(Xe/Xb), where T = time of incubation, Xe = number of cells at the end of incubation time, and Xb = number of cells at the beginning of the incubation time and absolute cell number was plotted (Figure S4C).

Genomic DNA isolation

For genomic DNA isolation, HNSCC cells (1×10^6) were seeded in a 100-mm plate, at 80% confluence, cells were trypsinized and used for genomic DNA isolation using QIAamp DNA mini kit as per the manufacturer’s instructions. Briefly, pellets were suspended in 200 μL 1× PBS (137 mM NaCl, 2.7 mM KCl, 10 mM Na_2HPO_4, and 1.8 mM KH_2PO_4) followed by the addition of 20 μL proteinase K solution and 200 μL of buffer AL (provided with the kit) into the mixture, mixed by pulse-vortexing, and incubated at 56°C for 10 min with occasional pulse-vortexing during the incubation. After a mini spin, 200 μL ethanol (96%–100%) was added to the mixture mixed again by pulse-vortexing, and loaded onto the spin columns. Spin columns were centrifuged at 6,000×*g* for 1 min at room temperature, flow through was discarded, and columns were washed with 500 μL of buffer AW1 and then with 500 μL of buffer AW2. We used 50 μL buffer AE to elute the genomic DNA obtained using the kit. The concentration of eluted DNA was quantified using NanoDrop One-C

(Thermo Scientific) using buffer AE as blank, and quality was assured as per the readings of A260/A280 and A260/A230 ratios. Isolated genomic DNA was subjected to 1% agarose gel electrophoresis for 1 h at 80 V in 0.5× Tris-borate buffer in the presence of ethidium bromide (EtBr) (0.1 μg/mL) and visualized on Amersham Imager 600 (Cytiva) (Figures S5A and S5B).

HPV consensus and HPV16 type-specific genomic PCR

HPV infection was detected by PCR-based method using PGMY (09/11) L1 consensus primers[95] and HPV16 type-specific primers for E6, E7, and LCR regions as described earlier.[96] Briefly, 100 ng of HNSCC cell DNA was amplified using PCR master mix with 80 nM of PGMY09 and PGMY11 each set in BioMetra TAdvanced Thermal Cycler (Analytik Jena). The temperature profile used for amplification constituted an initial denaturation at 95°C for 9 min followed by 35 cycles of denaturation at 95°C for 30 s, annealing at 55°C for 90 s, extension at 72°C for 2 min, which was extended for 5 min at the final cycle. Similarly, following the same reaction conditions, the HPV16-specific PCR was performed using primers specific for HPV16-E6, E7, LCR, and β-actin (Table S5).

The PCR products were analyzed using 2% agarose gel stained with EtBr (0.1 μg/mL) and visualized on the Amersham gel documentation system (Cytiva). UDSCC2 and 93VU147T showed positivity for HPV16, whereas OCT1, UPCI:SCC084, and UPCI:SCC131 were negative for HPV16 primers (Figures S5C and S5D). International standard for HPV16 DNA from whom was used as positive control whereas nuclease-free water was taken as negative control.

Mycoplasma screening

Periodically cells were tested by custom PCR to be mycoplasma contamination free as shown in Figure S5D (last panel). All cells were ensured to be mycoplasma-free culture.

Evaluation of HPV transcripts in HNSCC cells

RNA isolation

Total RNA was extracted from HNSCC cells (1×10^6 cells) using the RNeasy Mini Kit according to the manufacturer's instructions. Briefly, added buffer RLT to the cell pellet and thoroughly mix the contents. Transfer this mixture to an RNeasy Mini spin column to bind the RNA through centrifugation at maximum speed. Subsequently, perform a series of wash steps by sequentially washing the column with Buffer RW1 and Buffer RPE, followed by an additional wash with Buffer RPE. Place the spin column in a new collection tube and elute the RNA by adding RNase-free water directly to the membrane. Centrifuge the column to collect the eluted RNA. The RNA was quantified spectrophotometrically using Nanodrop One-C (Thermo). Finally, store the isolated RNA at −80°C refrigerator (Haier) for future use or proceed with downstream applications.

cDNA preparation and RT-PCR

RNA from HPV-positive and HPV-negative HNSCC were isolated using TRIzol RNA isolation reagent as described earlier.[97] Complementary DNA (cDNA) was prepared from 10 μg of RNA sample in a 20 μL reaction using a High-Capacity Verso cDNA synthesis kit. For amplification of the HPV16 E6 and E7 gene and GAPDH was used as internal control, PCR was performed on Biometra Tadvanced Thermal Cycler (Analytik Jena) in a 25 μL reaction system. The PCR was programmed with initial denaturation for 5 min at 95°C, 40 cycles of denaturation at 95°C for 10 s, annealing at for 15 s as per annealing temperature standardized for each primer, followed by polymerization at 72°C for 10 s and final extension at 65°C for 1 min. The primer sequences and other details are listed in Table S4.

LCR-associated transcription factors

Manual mapping of consensus binding sites of transcription factors on HPV16 LCR

The consensus sites identified through a literature survey were manually mapped onto the FASTA format of HPV16 LCR (RefSeq: NC_001526.4) (Figure S6A). The literature survey encompassed experimentally proven, bioinformatically analyzed, and putative binding sites for transcription factors reported to interact with the HPV genome (previously published in Aggarwal et al[6]).

Find individual motif occurrences analysis

The binding motif enrichment analysis for LCR-associated transcription factors on HPV16 reference (RefSeq: NC_001526.4) genome was performed using find individual motif occurrences (FIMO) from the MEME package (https://meme-suite.org/meme/doc/fimo.html)[98] (Figure S6B). The nucleotide sequence of HPV16 LCR was retrieved from the NCBI database in FASTA format. The binding motifs for various LCR-associated transcription factors were downloaded from the JASPAR database. For input, the LCR-associated transcription factors available in literature consensus binding motif in meme format and HPV16 LCR sequence in FASTA format was uploaded. FIMO analysis was performed with the following parameters: # Scan: DNA motif on both strands; # Match $p < 0.01$. Only sequences with at least one consensus binding motif with a p value of <0.01 were considered as possible targets.

TFBIND analysis

The unsupervised exploration for potential LCR-associated transcription factor sequences within the HPV16 LCR sequence in FASTA format was initiated by uploading the LCR data on TFBIND online tool (https://tfbind.hgc.jp/) (Figure S6C). The potential transcriptional binders of the HPV16 LCR sequence was obtained.

Expression profiling of LCR-associated transcription factors

Isolation of cellular protein and immunoblotting

Total cellular proteins were isolated from HNSCC cells as described earlier.[99] Briefly, 1×10^6 cells were re-suspended in the cell lysis buffer [20 mM Tris (pH 7.4), 250 mM NaCl, 2 mM EDTA (pH 8.0), 0.1% Triton X-100, 0.01 mg/mL aprotinin, 0.005 mg/mL leupeptin, 0.4 mM PMSF, and 4 mM Na_3VO_4]. Lysates were spun at 14,000 rpm in a microfuge for 10 min to remove insoluble material and clear supernatant for each sample was collected. The concentration of total proteins was determined by the Bradford spectrophotometric evaluation at 595 nm against a BSA standard curve. Proteins

were stored in small aliquots at −80°C until further use. Proteins (25 μg/lane) were resolved in 10% polyacrylamide gel using 2× Laemmli buffer (100 mM Tris–HCL pH 8.0, 20 mM EDTA pH 8.0, 4% SDS, 20% glycerol, 10% β-mercaptoethanol, 0.02% bromophenol blue) and transferred to PVDF membranes (0.45 μm; Millipore) by semi-dry transfer method at 25 V for 2 h using *trans*-blot SD Semi-dry transfer cell (Bio-Rad). Membranes were blocked with 5% (w/v) bovine serum albumin in tris-buffered saline (TBS: 150 mM NaCl, 50 mM Tris-HCl, pH 7.6) supplemented with Tween 20 (0.1%) (TBST) for 2 h, and incubated with pre-standardized dilution of primary antibodies in TBST overnight at 4°C. Antibodies and their specific dilution in the blocking solution used in the study are described in Table S5. Membranes were washed with TBST and were incubated with horseradish peroxidase-conjugated secondary antibodies diluted in 5% BSA in TBST for 60 min at room temperature. The blot was re-probed subsequently with other 1° antibodies for the next round of detection or with β-actin. The absence of leftover signals after stripping was ascertained at the end of each cycle before the next reprobing (maximum of seven cycles of stripping and probing was performed). The western blot membranes were stripped at each interval using mild stripping buffer (1.5% glycine, 0.1% SDS, 1% Tween 20 pH-2.2) for 15 min at room temperature followed by re-blocking. Immuno-active bands were detected on a ChemiDoc-XRS (Bio-Rad) imaging system or Amersham Imager 600 after 5 min treatment of the blot with an enhanced chemiluminescent substrate luminol detection kit. Levels of β-actin protein in each sample were used as an internal control. The quantitative densitometric analysis of the bands was performed using ImageJ software. β-actin band intensity was used for normalizing the band intensity of oncoproteins and transcription factors. For comparison purposes, the relative protein level of aggregated normalized fold change in band intensities of oncoproteins and transcription factors, OCT1 was uniformly taken as a reference and its value was taken as 1.

Subcellular localization of LCR-associated transcription factors

Immunocytochemistry and fluorescence microscopy

Subcellular localization of host transcription factors and viral oncoproteins was determined by ICC as described earlier[100] with minor modifications. HNSCC cells were seeded on coverslips in 24 well plates at a density of 2,000 cells/well. Following 24 h, the medium was removed, and cells were rinsed with 1X PBS for 2 times. Cells were fixed in chilled methanol for 15 min and washed with 1X PBS. Cells were blocked with 5% FBS in PBS with Tween 20 (0.2%) (PBST) for 2 h and incubated overnight with respective primary antibodies listed in Table S5 for overnight followed by incubation with fluorescence-tagged secondary antibodies for 2 h. Counterstaining and mounting were done with Fluoroshield with DAPI on a microscope slide. Preparations were visualized using an ECLIPSE Ti2R microscope (Nikon, Minato City, Japan) on 405nm and 561nm lasers with 100× objective, total magnification 1000X.

Image analysis

Fluorescence intensity analyses were performed using Fiji, an image processing package of ImageJ software (https://imagej.net/software/fiji/).[101] To calculate percentage of nuclear intensity, nuclear and cytoplasmic intensities were measured for three independent field of view as per described earlier[102] and outlined in Figure S7. Nuclear intensity was calculated using the formula:

$$\text{Percentage nuclear intensity} = \frac{\text{Nuclear intensity}}{(\text{Nuclear intensity}+\text{Cytoplasmic intensity})} \times 100$$

Expression and survival analysis of LCR-Associated transcription factors in HNSCC patients

Expression analysis

UALCAN is a comprehensive, user-friendly, interactive web resource for analyzing cancer OMICS data. We used the UALCAN database (http://ualcan.path.uab.edu/analysis.html) to analyze LCR-specific transcription factor gene expression in HNSCC and normal tissues in the TCGA database based on HPV readcounts' perspective. Further, the statistical p values were noted.[93]

Kaplan-Meier survival analysis

For the present study, the dataset "Mixed Head and Neck (2022-v32)-tcga-548-tpm-gencode36" (N = 548) was used. Kaplan–Meier survival analysis was conducted to evaluate the prognostic significance of inducible and constitutive LCR-associated transcription factors in relation to HPV status. The analysis was performed using the R2 Genomics and Visualization Platform (http://r2platform.com). The 'Single Gene – Kaplan Meier' analysis option was applied, using the 'Separate by' setting to stratify patients survival based on expression levels of individual LCR-associated transcription factors genes. HPV classification track used for data separation, "hpv_status_by_p16_testing (3cat)", categorized samples into three groups: HPV-positive (N = 32), HPV-negative (N = 74), and not-defined (N = 442). To identify the optimal prognostic threshold, the Kaplan scan feature was employed, which determined the most significant mRNA expression cut-off to segregate patients (with available data) into good and poor prognosis groups.

Evaluation of HPV16 LCR reporter activity in HNSCC cells

Competent cell preparation

Colonies of *E. coli-DH5α* are isolated from glycerol stock. Single colonies will be inoculated in 2 mL media overnight. Inoculate approximately 100 μL of the cultures into 50 mL of pre-warmed respective media to facilitate growth to the early log phase. Monitor the optical

density (OD600) using a spectrometer (Cytiva) until the OD reaches 0.4. Subsequently, promptly cool the culture on ice for 15 min and proceed with centrifugation. The resulting pellets will be resuspended in CaCl2 to induce the chemical competence of the cells. All centrifugation steps will be performed at 3200 g using NEYA 16R refrigerated centrifuge (Remi, Mumbai, India) at 4°C. The aliquots of competent cells will be snap frozen in a −80°C refrigerator.

Transformation of plasmids in E. coli *strains*

For the transformation of plasmids, DH5α was employed following the standard protocol. Initially, competent DH5α *E. coli* was thawed on ice. Subsequently, 100 ng of plasmid was added to 10 μL of competent cells, followed by incubation on ice for 5 min. The pellets were then subjected to heat shock at 47°C in a water bath for 45s. After the heat shock, the tubes were immediately placed on ice for 5 min. Later, 1 mL of LB was added and the mixture was placed in an incubator at 37°C with a shaking speed of 200 rpm for 1 h for outgrowth. The culture was then centrifuged, and the pellet was plated on an LB agar plate with a suitable antibiotic.

Expansion of endotoxin-free plasmid

The expansion of plasmids was performed using the EndoFree Plasmid Maxi Kit following the manufacturer's instructions. Briefly, a single colony from a freshly streaked selective plate was inoculated into a 100 mL LB culture with the appropriate selective antibiotic. The culture was then incubated overnight at 37°C with vigorous shaking. After incubation, the bacterial culture was centrifuged at 6000 x g for 15 min at 4°C. The pellet was resuspended in 10 mL Buffer P1, followed by the addition of 10 mL Buffer P2. The tube was inverted 4–6 times and incubated at room temperature for 5 min. Subsequently, 10 mL chilled Buffer P3 was added, mixed immediately, and the cap was screwed onto the outlet nozzle of the QIAfilter Maxi Cartridge. The lysate was poured into the QIAfilter Cartridge barrel and incubated at room temperature for 10 min without inserting the plunger. Following this, the plunger was gently inserted into the QIAfilter Maxi Cartridge, and the lysate was filtered into a 50 mL tube. For DNA elution, 2.5 mL Buffer ER was added, mixed, and incubated on ice for 30 min. The eluate was then applied to a QIAGEN-tip 500, washed with Buffer QC, and DNA was eluted with Buffer QN. Finally, DNA precipitation was performed by adding isopropanol, followed by centrifugation, washing the pellet with ethanol, air-drying, and redissolving in Buffer TE. The concentration of eluted plasmid DNA was quantified using NanoDrop One-C (Thermo) using buffer TE as blank, and quality was assured as per the readings of A260/A280 and A260/A230 ratios. The purified endotoxin-free plasmid DNA was stored at 4°C.

Restriction digestion of plasmids

The plasmids were verified through restriction digestion using SacI and BamHI. For the digestion, 1 μg of plasmid DNA and 10 units of the respective restriction enzyme were used. The reaction was prepared in CutSmart 10X buffer and incubated for 1 h at 37°C in a water bath. The digested plasmids were run on 1% agarose gel electrophoresis in 1X TAE buffer in the presence of EtBr (0.1 μg/mL) and visualized using the Amersham Imager 600 (Cytiva).

HPV16 LCR-specific genomic PCR

The previously described HPV16 LCR-specific PCR was executed. Validation of the HPV16-LCR region in both pGL4-HPV16-LCR (nt 7000-100)-Luciferase and pGL4-HPV16-LCR (nt 7000-85)-Luciferase was carried out through LCR-specific PCR.

Transient transfections

Twenty-four hours before transfections, OCT1, UPCI:SCC084, UPCI:SCC131, UDSCC2, and 93VU147T cells were seeded in 12-well plates at densities of 10^6, 10^6, 10^6, 2×10^6, and 1.5×10^6 cells/well, respectively, to result in subconfluent monolayers. Cells were transfected with Lipofectamine 2000/DNA complexes (a total of 1μg LCR reporter plasmid/vector control and 25 ng of renilla reporter (pRL) in 3 μL Lipofectamine 2000) at 90–95% confluence. Following a 6-h interval, the medium was exchanged with fresh medium, and the cells were then incubated for an additional 48 h.

Dual luciferase assay

Dual luciferase assay was performed as per the manufacturer's instructions. Briefly, the transfected cells were washed with 1X PBS followed by addition of 250 μL of 1X passive lysis buffer. After lysis for 15 min, a 10 μL aliquot was used for luminescence measurements with a Synergy HTX Multimode Reader (BioTek, Winooski, U.S.). 100 μL of the firefly luciferase reagent (LARII) was added to the lysed sample, with a 10s equilibration time and measurement of luminescence with a 10s integration time, followed by addition of 100 μL of the Rluc reagent and firefly quenching (Stop and Glo), 10s equilibration time, and measurement of luminescence with a 10s integration time. The data are represented as the ratio of Firefly to Rluc activity (Fluc/Rluc). The luciferase activity of HPV16 LCR plasmids was normalized with respect to the empty vector pGL4.20 [luc2/puro] as follows:

$$\textbf{Fold Change}\ (\mathrm{FC}) = \frac{\text{Firefly Luciferase value}}{\text{Renilla Luciferase value}} \quad \textbf{Relative Fold Change} = \frac{\text{FC of HPV16 LCR plasmid}}{\text{FC of Empty vector}}$$

Statistical analysis

All experiments were performed in triplicate. The data analysis was performed using GraphPad Prism (Version 8.0.2 for Windows) (www.graphpad.com) and Microsoft Excel 2016. For statistical analysis, Normality and Lognormality test, Multiple Regression Analysis,

One-way ANOVA, two-way ANOVA, the Student t test (two-tailed distribution and two-sample unequal variance), Welch's t test, and multiple comparisons were applied. In the analysis of transcriptomic data, Welch's t test was used to assess the significance of expression level variations between normal and primary tumors (based on HPV read counts). In all cases, a p value of ≤ 0.05 was considered statistically significant.

DATA AND CODE AVAILABILITY

The data that support the findings of this study are available on request from the corresponding author, Prof. Alok Chandra Bharti.

ACKNOWLEDGMENTS

The study was partly supported by research grants from Indian Council of Medical Research (ICMR-ICRC) to A.C.B. (No.5/13/4/ACB/ICRC/2020/NCD-III), CCRH to A.C.B. (F.No.1-46/2022-23/CCRH/Tech./DM-RCT-Telomerase/Part-I/1588), Institution of Eminence University of Delhi (Ref. No./IoE/2023-24/12/FRP), ICMR-AdHOC to A.C.B. (2021-10573/GENOMIC/ADHOC-BMS), CSIR to N.A. (09/045(1622)/2018-EMR-I), D.J. (09/0045/(11635)/2021-EMR-1), A.Chaudhary (09/0045(12901)/2022-EMR-1), to J.Y. (09/045(1629)/2019-EMR-I), CSIR-UGC to T.T. (764/(CSIR-UGC NET JUNE 2019) and A.Chhokar [573(CSIR-UGC NET JUNE 2017)]. The authors thank Prof. Ashok Kumar, Professor of Pharmacology, Else and Philip Hargrove Endowed Professor of Drug Discovery, Department of Pharmacological and Pharmaceutical Sciences, University of Houston College of Pharmacy, Houston, Texas, for their cooperation in providing plasmids. The authors thank Mr. Robinsh Kamboj at the University of Delhi (USIC facility) for kind help.

AUTHOR CONTRIBUTIONS

A.C.B. and N.A. participated in conceptualization, formal writing and manuscript preparation. D.J. and A.Chaudhary contributed to manuscript preparation. U.J., T.T., C.C.K., J.Y., and A.Chhokar: advisory in manuscript preparation. A.C.B. conceived the presented idea and designed the manuscript, and critically reviewed, drafted the manuscript.

DECLARATION OF INTERESTS

The authors declare that they have no known competing financial interests or personal relationships that could have appeared to influence the work reported in this paper.

SUPPLEMENTAL INFORMATION

Supplemental information can be found online at https://doi.org/10.1016/j.omton.2024.200926.

REFERENCES

1. Sung, H., Ferlay, J., Siegel, R.L., Laversanne, M., Soerjomataram, I., Jemal, A., and Bray, F. (2021). Global Cancer Statistics 2020: GLOBOCAN Estimates of Incidence and Mortality Worldwide for 36 Cancers in 185 Countries. CA. Cancer J. Clin. *71*, 209–249. https://doi.org/10.3322/caac.21660.

2. Herrero, R., Castellsagué, X., Pawlita, M., Lissowska, J., Kee, F., Balaram, P., Rajkumar, T., Sridhar, H., Rose, B., Pintos, J., et al. (2003). Human papillomavirus and oral cancer: the International Agency for Research on Cancer multicenter study. J. Natl. Cancer Inst. *95*, 1772–1783. https://doi.org/10.1093/jnci/djg107.

3. Syrjänen, K., Syrjänen, S., Lamberg, M., Pyrhönen, S., and Nuutinen, J. (1983). Morphological and immunohistochemical evidence suggesting human papillomavirus (HPV) involvement in oral squamous cell carcinogenesis. Int. J. Oral Surg. *12*, 418–424. https://doi.org/10.1016/s0300-9785(83)80033-7.

4. Syrjänen, S. (2018). Oral manifestations of human papillomavirus infections. Eur. J. Oral Sci. *126*, 49–66. https://doi.org/10.1111/eos.12538.

5. Syrjänen, S., Andersson, B., Juntunen, L., and Syrjanen, K. (1992). Polymerase chain reaction for producing biotinylated human papillomavirus DNA probes for in situ hybridization. Sex. Transm. Dis. *19*, 140–145. https://doi.org/10.1097/00007435-199205000-00006.

6. Aggarwal, N., Yadav, J., Thakur, K., Bibban, R., Chhokar, A., Tripathi, T., Bhat, A., Singh, T., Jadli, M., Singh, U., et al. (2020). Human Papillomavirus Infection in Head and Neck Squamous Cell Carcinomas: Transcriptional Triggers and Changed Disease Patterns. Front. Cell. Infect. Microbiol. *10*, 537650. https://doi.org/10.3389/fcimb.2020.537650.

7. Kreimer, A.R., Clifford, G.M., Boyle, P., and Franceschi, S. (2005). Human papillomavirus types in head and neck squamous cell carcinomas worldwide: a systematic review. Cancer Epidemiol. Biomarkers Prev. *14*, 467–475. https://doi.org/10.1158/1055-9965.EPI-04-0551.

8. Gupta, S., Kumar, P., Kaur, H., Sharma, N., Saluja, D., Bharti, A.C., and Das, B.C. (2015). Selective participation of c-Jun with Fra-2/c-Fos promotes aggressive tumor phenotypes and poor prognosis in tongue cancer. Sci. Rep. *5*, 16811. https://doi.org/10.1038/srep16811.

9. Hammarstedt, L., Lindquist, D., Dahlstrand, H., Romanitan, M., Dahlgren, L.O., Joneberg, J., Creson, N., Lindholm, J., Ye, W., Dalianis, T., and Munck-Wikland, E. (2006). Human papillomavirus as a risk factor for the increase in incidence of tonsillar cancer. Int. J. Cancer *119*, 2620–2623. https://doi.org/10.1002/ijc.22177.

10. Pintos, J., Franco, E.L., Black, M.J., Bergeron, J., and Arella, M. (1999). Human papillomavirus and prognoses of patients with cancers of the upper aerodigestive tract. Cancer *85*, 1903–1909. https://doi.org/10.1002/(sici)1097-0142(19990501)85:9<1903::aid-cncr4>3.0.co;2-6.

11. Ang, K.K., Harris, J., Wheeler, R., Weber, R., Rosenthal, D.I., Nguyen-Tân, P.F., Westra, W.H., Chung, C.H., Jordan, R.C., Lu, C., et al. (2010). Human papillomavirus and survival of patients with oropharyngeal cancer. N. Engl. J. Med. *363*, 24–35. https://doi.org/10.1056/NEJMoa0912217.

12. Fakhry, C., Westra, W.H., Li, S., Cmelak, A., Ridge, J.A., Pinto, H., Forastiere, A., and Gillison, M.L. (2008). Improved survival of patients with human papillomavirus-positive head and neck squamous cell carcinoma in a prospective clinical trial. J. Natl. Cancer Inst. *100*, 261–269. https://doi.org/10.1093/jnci/djn011.

13. Fakhry, C., Zhang, Q., Nguyen-Tan, P.F., Rosenthal, D., El-Naggar, A., Garden, A.S., Soulieres, D., Trotti, A., Avizonis, V., Ridge, J.A., et al. (2014). Human papillomavirus and overall survival after progression of oropharyngeal squamous cell carcinoma. J. Clin. Oncol. *32*, 3365–3373. https://doi.org/10.1200/JCO.2014.55.1937.

14. Smith, E.M., Ritchie, J.M., Summersgill, K.F., Hoffman, H.T., Wang, D.H., Haugen, T.H., and Turek, L.P. (2004). Human papillomavirus in oral exfoliated cells and risk of head and neck cancer. J. Natl. Cancer Inst. *96*, 449–455. https://doi.org/10.1093/jnci/djh074.

15. Gillison, M.L., Koch, W.M., Capone, R.B., Spafford, M., Westra, W.H., Wu, L., Zahurak, M.L., Daniel, R.W., Viglione, M., Symer, D.E., et al. (2000). Evidence for a causal association between human papillomavirus and a subset of head and neck cancers. J. Natl. Cancer Inst. *92*, 709–720. https://doi.org/10.1093/jnci/92.9.709.

16. Shroyer, K.R., and Greer, R.O., Jr. (1991). Detection of human papillomavirus DNA by in situ DNA hybridization and polymerase chain reaction in premalignant and malignant oral lesions. Oral Surg. Oral Med. Oral Pathol. *71*, 708–713. https://doi.org/10.1016/0030-4220(91)90279-l.

17. Scheurlen, W., Stremlau, A., Gissmann, L., Höhn, D., Zenner, H.P., and zur Hausen, H. (1986). Rearranged HPV 16 molecules in an anal and in a laryngeal carcinoma. Int. J. Cancer *38*, 671–676. https://doi.org/10.1002/ijc.2910380509.

18. Snijders, P.J., Cromme, F.V., van den Brule, A.J., Schrijnemakers, H.F., Snow, G.B., Meijer, C.J., and Walboomers, J.M. (1992). Prevalence and expression of human papillomavirus in tonsillar carcinomas, indicating a possible viral etiology. Int. J. Cancer *51*, 845–850. https://doi.org/10.1002/ijc.2910510602.

19. Boscolo-Rizzo, P., Pawlita, M., and Holzinger, D. (2016). From HPV-positive towards HPV-driven oropharyngeal squamous cell carcinomas. Cancer Treat Rev. *42*, 24–29. https://doi.org/10.1016/j.ctrv.2015.10.009.

20. O'Connor, M., Chan, S.Y., and Bernard, H.U. (1995). Transcription Factor Binding Sites in the Long Control Region of. Genital *HPVs; part III-A*, 21–40.

21. Fontaine, V., van der Meijden, E., de Graaf, J., ter Schegget, J., and Struyk, L. (2000). A functional NF-kappaB binding site in the human papillomavirus type 16 long control region. Virology *272*, 40–49. https://doi.org/10.1006/viro.2000.0363.

22. Martínez-Ramírez, I., Del-Castillo-Falconi, V., Mitre-Aguilar, I.B., Amador-Molina, A., Carrillo-García, A., Langley, E., Zentella-Dehesa, A., Soto-Reyes, E., García-Carranca,

A., Herrera, L.A., and Lizano, M. (2017). SOX2 as a New Regulator of HPV16 Transcription. Viruses *9*, 175. https://doi.org/10.3390/v9070175.

23. Gloss, B., Bernard, H.U., Seedorf, K., and Klock, G. (1987). The upstream regulatory region of the human papilloma virus-16 contains an E2 protein-independent enhancer which is specific for cervical carcinoma cells and regulated by glucocorticoid hormones. EMBO J. *6*, 3735–3743.

24. Gloss, B., and Bernard, H.U. (1990). The E6/E7 promoter of human papillomavirus type 16 is activated in the absence of E2 proteins by a sequence-aberrant Sp1 distal element. J. Virol. *64*, 5577–5584.

25. Ishiji, T., Lace, M.J., Parkkinen, S., Anderson, R.D., Haugen, T.H., Cripe, T.P., Xiao, J.H., Davidson, I., Chambon, P., and Turek, L.P. (1992). Transcriptional enhancer factor (TEF)-1 and its cell-specific co-activator activate human papillomavirus-16 E6 and E7 oncogene transcription in keratinocytes and cervical carcinoma cells. EMBO J. *11*, 2271–2281.

26. Bauknecht, T., See, R.H., and Shi, Y. (1996). A novel C/EBP beta-YY1 complex controls the cell-type-specific activity of the human papillomavirus type 18 upstream regulatory region. J. Virol. *70*, 7695–7705.

27. Sichero, L., Sobrinho, J.S., and Villa, L.L. (2012). Identification of novel cellular transcription factors that regulate early promoters of human papillomavirus types 18 and 16. J. Infect. Dis. *206*, 867–874. https://doi.org/10.1093/infdis/jis430.

28. Bauknecht, T., Angel, P., Royer, H.D., and zur Hausen, H. (1992). Identification of a negative regulatory domain in the human papillomavirus type 18 promoter: interaction with the transcriptional repressor YY1. EMBO J. *11*, 4607–4617.

29. Gloss, B., Yeo-Gloss, M., Meisterenst, M., Rogge, L., Winnacker, E.L., and Bernard, H.U. (1989). Clusters of nuclear factor I binding sites identify enhancers of several papillomaviruses but alone are not sufficient for enhancer function. Nucleic Acids Res. *17*, 3519–3533. https://doi.org/10.1093/nar/17.9.3519.

30. Chong, T., Apt, D., Gloss, B., Isa, M., and Bernard, H.U. (1991). The enhancer of human papillomavirus type 16: binding sites for the ubiquitous transcription factors oct-1, NFA, TEF-2, NF1, and AP-1 participate in epithelial cell-specific transcription. J. Virol. *65*, 5933–5943.

31. Dong, X.P., Stubenrauch, F., Beyer-Finkler, E., and Pfister, H. (1994). Prevalence of deletions of YY1-binding sites in episomal HPV 16 DNA from cervical cancers. Int. J. Cancer *58*, 803–808. https://doi.org/10.1002/ijc.2910580609.

32. Pande, S., Jain, N., Prusty, B.K., Bhambhani, S., Gupta, S., Sharma, R., Batra, S., and Das, B.C. (2008). Human papillomavirus type 16 variant analysis of E6, E7, and L1 genes and long control region in biopsy samples from cervical cancer patients in north India. J. Clin. Microbiol. *46*, 1060–1066.

33. Xi, J., Chen, J., Xu, M., Yang, H., Luo, J., Pan, Y., Wang, X., Qiu, L., Yang, J., and Sun, Q. (2017). Genetic variability and functional implication of the long control region in HPV-16 variants in Southwest China. PLoS One *12*, e0182388. https://doi.org/10.1371/journal.pone.0182388.

34. Angel, P., and Karin, M. (1991). The role of Jun, Fos and the AP-1 complex in cell-proliferation and transformation. Biochim. Biophys. Acta *1072*, 129–157. https://doi.org/10.1016/0304-419x(91)90011-9.

35. Gaykalova, D.A., Manola, J.B., Ozawa, H., Zizkova, V., Morton, K., Bishop, J.A., Sharma, R., Zhang, C., Michailidi, C., Considine, M., et al. (2015). NF-kappaB and stat3 transcription factor signatures differentiate HPV-positive and HPV-negative head and neck squamous cell carcinoma. Int. J. Cancer *137*, 1879–1889. https://doi.org/10.1002/ijc.29558.

36. Gupta, S., Kumar, P., Kaur, H., Sharma, N., Gupta, S., Saluja, D., Bharti, A.C., and Das, B. (2018). Constitutive activation and overexpression of NF-kappaB/c-Rel in conjunction with p50 contribute to aggressive tongue tumorigenesis. Oncotarget *9*, 33011–33029. https://doi.org/10.18632/oncotarget.26041.

37. Mishra, A., Bharti, A.C., Saluja, D., and Das, B.C. (2010). Trans-activation and expression patterns of jun and fos/AP-1 super-family proteins in human oral cancer. Int. J. Cancer *126*, 819–829.

38. Mishra, A., Bharti, A.C., Varghese, P., Saluja, D., and Das, B.C. (2006). Differential expression and activation of NF-kappaB family proteins during oral carcinogenesis: Role of high risk human papillomavirus infection. Int. J. Cancer *119*, 2840–2850. https://doi.org/10.1002/ijc.22262.

39. Mishra, A., Kumar, R., Tyagi, A., Kohaar, I., Hedau, S., Bharti, A.C., Sarker, S., Dey, D., Saluja, D., and Das, B. (2015). Curcumin modulates cellular AP-1, NF-kB, and HPV16 E6 proteins in oral cancer. Ecancermedicalscience *9*, 525. https://doi.org/10.3332/ecancer.2015.525.

40. Verma, G., Vishnoi, K., Tyagi, A., Jadli, M., Singh, T., Goel, A., Sharma, A., Agarwal, K., Prasad, S.C., Pandey, D., et al. (2017). Characterization of key transcription factors as molecular signatures of HPV-positive and HPV-negative oral cancers. Cancer Med. *6*, 591–604. https://doi.org/10.1002/cam4.983.

41. Mori, S., Ishii, Y., Takeuchi, T., and Kukimoto, I. (2023). Nuclear proinflammatory cytokine S100A9 enhances expression of human papillomavirus oncogenes via transcription factor TEAD1. J. Virol. *97*, e0081523. https://doi.org/10.1128/jvi.00815-23.

42. Thakur, K., Janjua, D., Aggarwal, N., Chhokar, A., Yadav, J., Tripathi, T., Chaudhary, A., Senrung, A., Shrivastav, A., and Bharti, A.C. (2023). Physical interaction between STAT3 and AP1 in cervical carcinogenesis: Implications in HPV transcription control. Biochim. Biophys. Acta, Mol. Basis Dis. *1869*, 166817. https://doi.org/10.1016/j.bbadis.2023.166817.

43. Morgan, E.L., Scarth, J.A., Patterson, M.R., Wasson, C.W., Hemingway, G.C., Barba-Moreno, D., and Macdonald, A. (2021). E6-mediated activation of JNK drives EGFR signalling to promote proliferation and viral oncoprotein expression in cervical cancer. Cell Death Differ. *28*, 1669–1687. https://doi.org/10.1038/s41418-020-00693-9.

44. Morgan, E.L., Wasson, C.W., Hanson, L., Kealy, D., Pentland, I., McGuire, V., Scarpini, C., Coleman, N., Arthur, J.S.C., Parish, J.L., et al. (2018). STAT3 activation by E6 is essential for the differentiation-dependent HPV18 life cycle. PLoS Pathog. *14*, e1006975. https://doi.org/10.1371/journal.ppat.1006975.

45. Geiger, J.L., Grandis, J.R., and Bauman, J.E. (2016). The STAT3 pathway as a therapeutic target in head and neck cancer: Barriers and innovations. Oral Oncol. *56*, 84–92. https://doi.org/10.1016/j.oraloncology.2015.11.022.

46. Nottingham, L.K., Yan, C.H., Yang, X., Si, H., Coupar, J., Bian, Y., Cheng, T.F., Allen, C., Arun, P., Gius, D., et al. (2014). Aberrant IKKalpha and IKKbeta cooperatively activate NF-kappaB and induce EGFR/AP1 signaling to promote survival and migration of head and neck cancer. Oncogene *33*, 1135–1147. https://doi.org/10.1038/onc.2013.49.

47. Pentland, I., Campos-León, K., Cotic, M., Davies, K.J., Wood, C.D., Groves, I.J., Burley, M., Coleman, N., Stockton, J.D., Noyvert, B., et al. (2018). Disruption of CTCF-YY1-dependent looping of the human papillomavirus genome activates differentiation-induced viral oncogene transcription. PLoS Biol. *16*, e2005752. https://doi.org/10.1371/journal.pbio.2005752.

48. Alam, M., Kashyap, T., Pramanik, K.K., Singh, A.K., Nagini, S., and Mishra, R. (2017). The elevated activation of NFkappaB and AP-1 is correlated with differential regulation of Bcl-2 and associated with oral squamous cell carcinoma progression and resistance. Clin. Oral Invest. *21*, 2721–2731. https://doi.org/10.1007/s00784-017-2074-6.

49. Hyakusoku, H., Sano, D., Takahashi, H., Hatano, T., Isono, Y., Shimada, S., Ito, Y., Myers, J.N., and Oridate, N. (2016). JunB promotes cell invasion, migration and distant metastasis of head and neck squamous cell carcinoma. J. Exp. Clin. Cancer Res. *35*, 6. https://doi.org/10.1186/s13046-016-0284-4.

50. Young, M.R., Farrell, L., Lambert, P., Awasthi, P., and Colburn, N.H. (2002). Protection against human papillomavirus type 16-E7 oncogene-induced tumorigenesis by in vivo expression of dominant-negative c-jun. Mol. Carcinog. *34*, 72–77. https://doi.org/10.1002/mc.10050.

51. Badarni, M., Prasad, M., Balaban, N., Zorea, J., Yegodayev, K.M., Joshua, B.Z., Dinur, A.B., Grénman, R., Rotblat, B., Cohen, L., and Elkabets, M. (2019). Repression of AXL expression by AP-1/JNK blockage overcomes resistance to PI3Ka therapy. JCI Insight *5*, e125341. https://doi.org/10.1172/jci.insight.125341.

52. Xu, H., Jin, X., Yuan, Y., Deng, P., Jiang, L., Zeng, X., Li, X.S., Wang, Z.Y., and Chen, Q.M. (2017). Prognostic value from integrative analysis of transcription factors c-Jun and Fra-1 in oral squamous cell carcinoma: a multicenter cohort study. Sci. Rep. *7*, 7522. https://doi.org/10.1038/s41598-017-05106-5.

53. Bano, N., Yadav, M., Mohania, D., and Das, B.C. (2018). The role of NF-kappaB and miRNA in oral cancer and cancer stem cells with or without HPV16 infection. PLoS One *13*, e0205518. https://doi.org/10.1371/journal.pone.0205518.

54. Ondrey, F.G., Dong, G., Sunwoo, J., Chen, Z., Wolf, J.S., Crowl-Bancroft, C.V., Mukaida, N., and Van Waes, C. (1999). Constitutive activation of transcription factors NF-(kappa)B, AP-1, and NF-IL6 in human head and neck squamous cell carcinoma cell lines that express pro-inflammatory and pro-angiogenic cytokines. Mol.

Carcinog. *26*, 119–129. https://doi.org/10.1002/(sici)1098-2744(199910)26:2<119::aid-mc6>3.0.co;2-n.

55. Li, L., and Zhang, Z.T. (2019). Genetic Association between NFKBIA and NFKB1 Gene Polymorphisms and the Susceptibility to Head and Neck Cancer: A Meta-Analysis. Dis. Markers *2019*, 6523837. https://doi.org/10.1155/2019/6523837.

56. Chuerduangphui, J., Pientong, C., Chaiyarit, P., Patarapadungkit, N., Chotiyano, A., Kongyingyoes, B., Promthet, S., Swangphon, P., Wongjampa, W., and Ekalaksananan, T. (2016). Effect of human papillomavirus 16 oncoproteins on oncostatin M upregulation in oral squamous cell carcinoma. Med. Oncol. *33*, 83. https://doi.org/10.1007/s12032-016-0800-6.

57. Mazibrada, J., Longo, L., Vatrano, S., Cappia, S., Giorcelli, J., Pentenero, M., Gandolfo, S., Volante, M., dell'Oste, V., Lo Cigno, I., et al. (2014). Differential expression of HER2, STAT3, SOX2, IFI16 and cell cycle markers during HPV-related head and neck carcinogenesis. New Microbiol. *37*, 129–143.

58. Shukla, S., Jadli, M., Thakur, K., Shishodia, G., Mahata, S., Basir, S.F., Das, B.C., and Bharti, A.C. (2019). Level of phospho-STAT3 (Tyr705) correlates with copy number and physical state of human papillomavirus 16 genome in cervical precancer and cancer lesions. PLoS One *14*, e0222089. https://doi.org/10.1371/journal.pone.0222089.

59. Shukla, S., Shishodia, G., Mahata, S., Hedau, S., Pandey, A., Bhambhani, S., Batra, S., Basir, S.F., Das, B.C., and Bharti, A.C. (2010). Aberrant expression and constitutive activation of STAT3 in cervical carcinogenesis: implications in high-risk human papillomavirus infection. Mol. Cancer *9*, 282. https://doi.org/10.1186/1476-4598-9-282.

60. Mohamed, H., Aro, K., Jouhi, L., Mäkitie, A., Remes, S., Haglund, C., Atula, T., and Hagström, J. (2018). Expression of hormone receptors in oropharyngeal squamous cell carcinoma. Eur. Arch. Oto-Rhino-Laryngol. *275*, 1289–1300. https://doi.org/10.1007/s00405-018-4949-9.

61. Dogan, S., Xu, B., Middha, S., Vanderbilt, C.M., Bowman, A.S., Migliacci, J., Morris, L.G.T., Seshan, V.E., and Ganly, I. (2019). Identification of prognostic molecular biomarkers in 157 HPV-positive and HPV-negative squamous cell carcinomas of the oropharynx. Int. J. Cancer *145*, 3152–3162. https://doi.org/10.1002/ijc.32412.

62. Behera, A.K., Kumar, M., Shanmugam, M.K., Bhattacharya, A., Rao, V.J., Bhat, A., Vasudevan, M., Gopinath, K.S., Mohiyuddin, A., Chatterjee, A., et al. (2019). Functional interplay between YY1 and CARM1 promotes oral carcinogenesis. Oncotarget *10*, 3709–3724. https://doi.org/10.18632/oncotarget.26984.

63. Lyu, X.M., Zhu, X.W., Zhao, M., Zuo, X.B., Huang, Z.X., Liu, X., Jiang, T., Yang, X.X., Li, X., Long, X.B., et al. (2018). A regulatory mutant on TRIM26 conferring the risk of nasopharyngeal carcinoma by inducing low immune response. Cancer Med. *7*, 3848–3861. https://doi.org/10.1002/cam4.1537.

64. Nakayama, Y., Kobayashi, R., Iwai, Y., Noda, K., Yamazaki, M., Kurita-Ochiai, T., Yoshimura, A., Ganss, B., and Ogata, Y. (2019). C/EBPbeta and YY1 bind and interact with Smad3 to modulate lipopolysaccharide-induced amelotin gene transcription in mouse gingival epithelial cells. FEBS Open Bio *9*, 276–290. https://doi.org/10.1002/2211-5463.12566.

65. Holmes, B.J., Maleki, Z., and Westra, W.H. (2015). The Fidelity of p16 Staining as a Surrogate Marker of Human Papillomavirus Status in Fine-Needle Aspirates and Core Biopsies of Neck Node Metastases: Implications for HPV Testing Protocols. Acta Cytol. *59*, 97–103. https://doi.org/10.1159/000375148.

66. Hyakusoku, H., Sawakuma, K., Sano, D., Takahashi, H., Hatano, T., Sato, K., Isono, Y., Shimada, S., Takada, K., Kuwahara, T., et al. (2021). FosL1 Regulates Regional Metastasis of Head and Neck Squamous Cell Carcinoma by Promoting Cell Migration, Invasion, and Proliferation. Anticancer Res. *41*, 3317–3326. https://doi.org/10.21873/anticanres.15119.

67. Chen, S., Youhong, T., Tan, Y., He, Y., Ban, Y., Cai, J., Li, X., Xiong, W., Zeng, Z., Li, G., et al. (2020). EGFR-PKM2 signaling promotes the metastatic potential of nasopharyngeal carcinoma through induction of FOSL1 and ANTXR2. Carcinogenesis *41*, 723–733. https://doi.org/10.1093/carcin/bgz180.

68. Peng, L., Sun, E., Zhang, J., Cai, L., Zheng, J., and Zeng, Y. (2024). The effect of c-Fos on the prognosis, proliferation, and invasion of oral squamous cell carcinoma. Oral Dis. *30*, 4266–4277. https://doi.org/10.1111/odi.14892.

69. Krishna, A., Bhatt, M.L.B., Singh, V., Singh, S., Gangwar, P.K., Singh, U.S., Kumar, V., and Mehrotra, D. (2018). Differential Expression of c-fos Proto-Oncogene in Normal Oral Mucosa versus Squamous Cell Carcinoma. Asian Pac. J. Cancer Prev. APJCP *19*, 867–874. https://doi.org/10.22034/APJCP.2018.19.3.867.

70. Wang, S., Xu, X., Xu, F., Meng, Y., Sun, C., Shi, L., and Zhao, E. (2016). Combined Expression of c-jun, c-fos, and p53 Improves Estimation of Prognosis in Oral Squamous Cell Carcinoma. Cancer Invest. *34*, 393–400. https://doi.org/10.1080/07357907.2016.1217422.

71. Patel, D., Dabhi, A.M., Dmello, C., Seervi, M., Sneha, K.M., Agrawal, P., Sahani, M.H., and Kanojia, D. (2022). FKBP1A upregulation correlates with poor prognosis and increased metastatic potential of HNSCC. Cell Biol. Int. *46*, 443–453. https://doi.org/10.1002/cbin.11741.

72. Liu, Y.T., Zhao, F.P., Miao, H.B., Fu, S.Z., Zhou, S., Zhang, X.G., and Qin, G. (2018). [Expression of C-fos in nasopharyngeal carcinoma and the relationship with chemosensitivity and prognosis]. Lin chuang er bi yan hou tou jing wai ke za zhi = Journal of clinical otorhinolaryngology head and neck surgery *32*, 687–692. https://doi.org/10.13201/j.issn.1001-1781.2018.09.011.

73. Liu, Q., Yang, T., Zhang, Y., Hu, Z.D., Liu, Y.M., Luo, Y.L., Liu, S.X., Zhang, H., and Zhong, Q. (2023). ZIC2 induces pro-tumor macrophage polarization in nasopharyngeal carcinoma by activating the JUNB/MCSF axis. Cell Death Dis. *14*, 455. https://doi.org/10.1038/s41419-023-05983-x.

74. Zhong, J.T., Wang, H.J., Yu, J., Zhang, J.H., Wang, S.F., Yang, X., and Su, W. (2017). Correlations of the expressions of c-Jun and Egr-1 proteins with clinicopathological features and prognosis of patients with nasopharyngeal carcinoma. Cancer Biomarkers *19*, 213–220. https://doi.org/10.3233/CBM-161710.

75. Ghosh, S., Talukdar, P.D., Bhattacharjee, A., Giri, S., Bhattacharyya, N.P., and Chatterji, U. (2021). JunD accentuates arecoline-induced disruption of tight junctions and promotes epithelial-to-mesenchymal transition by association with NEAT1 lncRNA. Oncotarget *12*, 1520–1539. https://doi.org/10.18632/oncotarget.28026.

76. Rades, D., Huttenlocher, S., Seibold, N.D., Gebhard, M.P., Thorns, C., Hasselbacher, K., Wollenberg, B., and Schild, S.E. (2015). Nuclear expression of p65 (RelA) in patients receiving post-operative radiotherapy for locally advanced squamous cell carcinoma of the head and neck. BMC Cancer *15*, 102. https://doi.org/10.1186/s12885-015-1107-2.

77. Balermpas, P., Michel, Y., Wagenblast, J., Seitz, O., Sipek, F., Rödel, F., Rödel, C., and Fokas, E. (2013). Nuclear NF-kappaB expression correlates with outcome among patients with head and neck squamous cell carcinoma treated with primary chemoradiation therapy. Int. J. Radiat. Oncol. Biol. Phys. *86*, 785–790. https://doi.org/10.1016/j.ijrobp.2013.04.001.

78. Giopanou, I., Lilis, I., Papadaki, H., Papadas, T., and Stathopoulos, G.T. (2017). A link between RelB expression and tumor progression in laryngeal cancer. Oncotarget *8*, 114019–114030. https://doi.org/10.18632/oncotarget.23109.

79. Li, J., Wang, W., Chen, S., Cai, J., Ban, Y., Peng, Q., Zhou, Y., Zeng, Z., Li, X., Xiong, W., et al. (2019). FOXA1 reprograms the TGF-beta-stimulated transcriptional program from a metastasis promoter to a tumor suppressor in nasopharyngeal carcinoma. Cancer Lett. *442*, 1–14. https://doi.org/10.1016/j.canlet.2018.10.036.

80. Ammous-Boukhris, N., Ayadi, W., Derbel, M., Allaya-Jaafar, N., Charfi, S., Daoud, J., Sellami-Boudawara, T., and Mokdad-Gargouri, R. (2020). FOXA1 Expression in Nasopharyngeal Carcinoma: Association with Clinicopathological Characteristics and EMT Markers. BioMed Res. Int. *2020*, 4234632. https://doi.org/10.1155/2020/4234632.

81. Wang, J., Kang, M., Qin, Y.T., Wei, Z.X., Xiao, J.J., and Wang, R.S. (2015). Sp1 is over-expressed in nasopharyngeal cancer and is a poor prognostic indicator for patients receiving radiotherapy. Int. J. Clin. Exp. Pathol. *8*, 6936–6943.

82. Xu, C., Shen, Y., Shi, Y., Zhang, M., and Zhou, L. (2022). Eukaryotic translation initiation factor 3 subunit B promotes head and neck cancer via CEBPB translation. Cancer Cell Int. *22*, 161. https://doi.org/10.1186/s12935-022-02578-y.

83. Schnoell, J., Jank, B.J., Kadletz-Wanke, L., Stoiber, S., Spielvogel, C.P., Gurnhofer, E., Kenner, L., and Heiduschka, G. (2021). Transcription factors CP2 and YY1 as prognostic markers in head and neck squamous cell carcinoma: analysis of The Cancer Genome Atlas and a second independent cohort. J. Cancer Res. Clin. Oncol. *147*, 755–765. https://doi.org/10.1007/s00432-020-03482-6.

84. Ottinger, M., Smith, J.A., Schweiger, M.R., Robbins, D., Powell, M.L.C., You, J., and Howley, P.M. (2009). Cell-type specific transcriptional activities among different

papillomavirus long control regions and their regulation by E2. Virology *395*, 161–171. https://doi.org/10.1016/j.virol.2009.09.027.

85. Bojilova, E.D., Weyn, C., Antoine, M.H., and Fontaine, V. (2016). Extrachromosomal HPV-16 LCR transcriptional activation by HDACi opposed by cellular differentiation and DNA integration. Oncotarget *7*, 75526–75538. https://doi.org/10.18632/oncotarget.12263.

86. Pientong, C., Wongwarissara, P., Ekalaksananan, T., Swangphon, P., Kleebkaow, P., Kongyingyoes, B., Siriaunkgul, S., Tungsinmunkong, K., and Suthipintawong, C. (2013). Association of human papillomavirus type 16 long control region mutation and cervical cancer. Virol. J. *10*, 30. https://doi.org/10.1186/1743-422X-10-30.

87. Weyn, C., Vanderwinden, J.M., Rasschaert, J., Englert, Y., and Fontaine, V. (2011). Regulation of human papillomavirus type 16 early gene expression in trophoblastic and cervical cells. Virology *412*, 146–155. https://doi.org/10.1016/j.virol.2010.12.056.

88. Lung, M.S.Y., Zhang, N., and Murray, V. (2012). Site-directed mutagenesis of human papillomavirus 18 promoter elements and tissue-specific expression in cervical carcinoma cells. Virus Gene. *44*, 395–402. https://doi.org/10.1007/s11262-012-0723-z.

89. Silva, R.C.O., Lima, R.d.C.P., Cordeiro, M.N., Dos Santos, D.L., Pena, L.J., Gurgel, A.P.A.D., Batista, M.V.A., Chagas, B.S., and Freitas, A.C. (2020). Functional evaluation of human papillomavirus type 31 long control region variants. Genomics *112*, 5066–5071. https://doi.org/10.1016/j.ygeno.2020.09.008.

90. Gyöngyösi, E., László, B., Szalmás, A., Kónya, J., and Veress, G. (2023). Transcriptional activity of the long control region in human papillomavirus type 33 intratype variants. Virol. J. *20*, 152. https://doi.org/10.1186/s12985-023-02114-y.

91. Lung, M.S.Y., Mak, W.M., and Murray, V. (2011). The use of a human papillomavirus 18 promoter for tissue-specific expression in cervical carcinoma cells. Cell. Mol. Biol. Lett. *16*, 477–492. https://doi.org/10.2478/s11658-011-0018-8.

92. Powell, M.L.C., Smith, J.A., Sowa, M.E., Harper, J.W., Iftner, T., Stubenrauch, F., and Howley, P.M. (2010). NCoR1 mediates papillomavirus E8;E2C transcriptional repression. J. Virol. *84*, 4451–4460. https://doi.org/10.1128/JVI.02390-09.

93. Chandrashekar, D.S., Karthikeyan, S.K., Korla, P.K., Patel, H., Shovon, A.R., Athar, M., Netto, G.J., Qin, Z.S., Kumar, S., Manne, U., et al. (2022). UALCAN: An update to the integrated cancer data analysis platform. Neoplasia *25*, 18–27. https://doi.org/10.1016/j.neo.2022.01.001.

94. Campbell, J.D., Yau, C., Bowlby, R., Liu, Y., Brennan, K., Fan, H., Taylor, A.M., Wang, C., Walter, V., Akbani, R., et al. (2018). Genomic, Pathway Network, and Immunologic Features Distinguishing Squamous Carcinomas. Cell Rep. *23*, 194–212.e6. https://doi.org/10.1016/j.celrep.2018.03.063.

95. Coutlée, F., Gravitt, P., Kornegay, J., Hankins, C., Richardson, H., Lapointe, N., Voyer, H., and Franco, E. (2002). Use of PGMY primers in L1 consensus PCR improves detection of human papillomavirus DNA in genital samples. J. Clin. Microbiol. *40*, 902–907. https://doi.org/10.1128/JCM.40.3.902-907.2002.

96. Bhat, A., Yadav, J., Thakur, K., Aggarwal, N., Chhokar, A., Tripathi, T., Singh, T., Jadli, M., Veerapandian, V., and Bharti, A.C. (2022). Transcriptome analysis of cervical cancer exosomes and detection of HPVE6*I transcripts in exosomal RNA. BMC Cancer *22*, 164. https://doi.org/10.1186/s12885-022-09262-4.

97. Senrung, A., Tripathi, T., Yadav, J., Janjua, D., Chaudhary, A., Chhokar, A., Aggarwal, N., Joshi, U., Goswami, N., and Bharti, A.C. (2023). In vivo antiangiogenic effect of nimbolide, trans-chalcone and piperine for use against glioblastoma. BMC Cancer *23*, 1173. https://doi.org/10.1186/s12885-023-11625-4.

98. Grant, C.E., Bailey, T.L., and Noble, W.S. (2011). FIMO: scanning for occurrences of a given motif. Bioinformatics *27*, 1017–1018. https://doi.org/10.1093/bioinformatics/btr064.

99. Bhat, A., Yadav, J., Thakur, K., Aggarwal, N., Tripathi, T., Chhokar, A., Singh, T., Jadli, M., and Bharti, A.C. (2021). Exosomes from cervical cancer cells facilitate pro-angiogenic endothelial reconditioning through transfer of Hedgehog-GLI signaling components. Cancer Cell Int. *21*, 319. https://doi.org/10.1186/s12935-021-02026-3.

100. Bharti, A.C., Shishodia, S., Reuben, J.M., Weber, D., Alexanian, R., Raj-Vadhan, S., Estrov, Z., Talpaz, M., and Aggarwal, B.B. (2004). Nuclear factor-kappaB and STAT3 are constitutively active in CD138+ cells derived from multiple myeloma patients, and suppression of these transcription factors leads to apoptosis. Blood *103*, 3175–3184. https://doi.org/10.1182/blood-2003-06-2151.

101. Schindelin, J., Arganda-Carreras, I., Frise, E., Kaynig, V., Longair, M., Pietzsch, T., Preibisch, S., Rueden, C., Saalfeld, S., Schmid, B., et al. (2012). Fiji: an open-source platform for biological-image analysis. Nat. Methods *9*, 676–682. https://doi.org/10.1038/nmeth.2019.

102. Kelley, J.B., and Paschal, B.M. (2019). Fluorescence-based quantification of nucleocytoplasmic transport. Methods *157*, 106–114. https://doi.org/10.1016/j.ymeth.2018.11.002.

OMTON, Volume *33*

Supplemental information

Insights into expression and localization of HPV16 LCR-associated transcription factors and association with LCR activity in HNSCC

Nikita Aggarwal, Divya Janjua, Apoorva Chaudhary, Udit Joshi, Tanya Tripathi, Chetkar Chandra Keshavam, Joni Yadav, Arun Chhokar, and Alok Chandra Bharti

Figure S1a

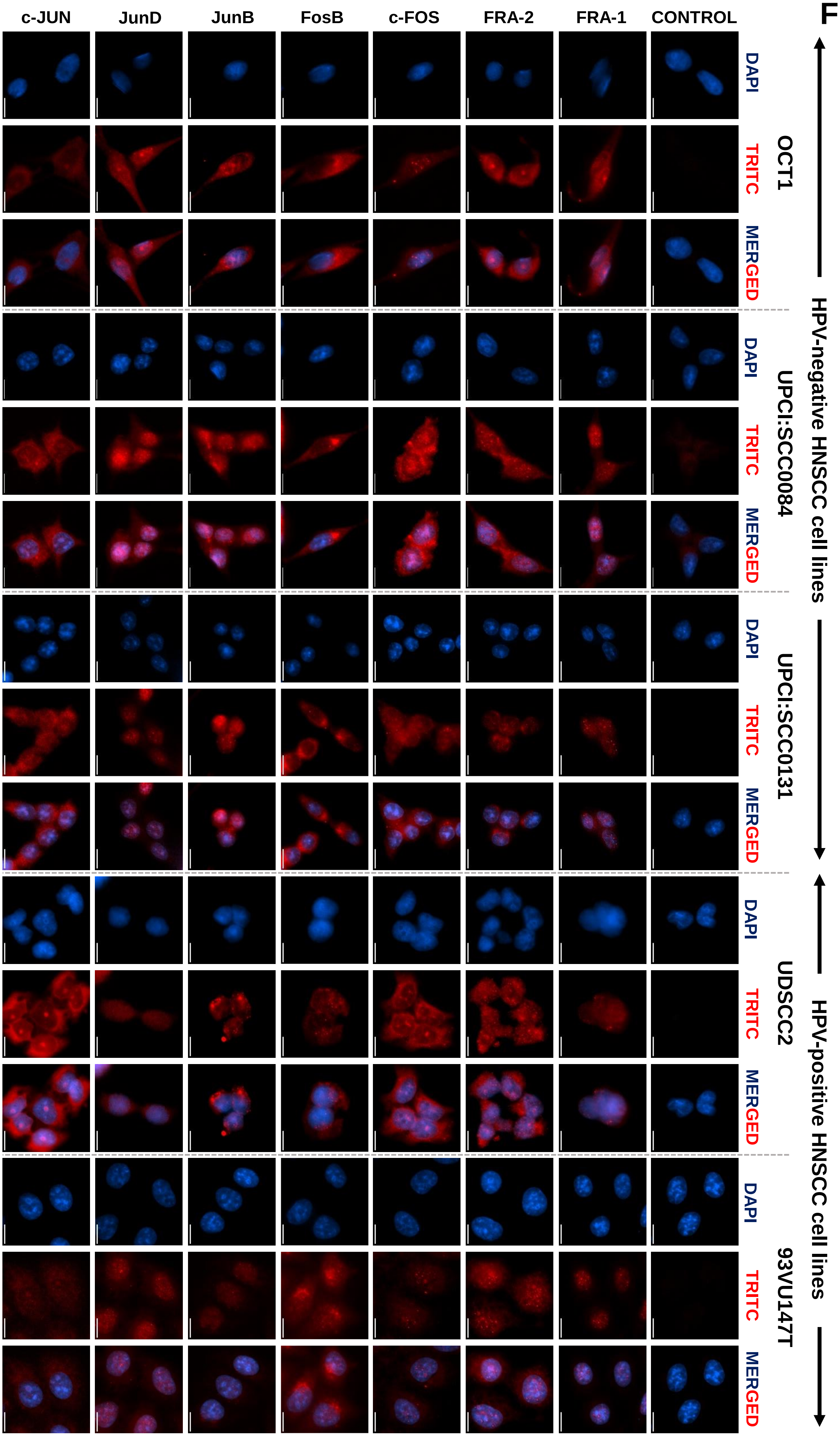
c-JUN
JunD
JunB
FosB
c-FOS
FRA-2
FRA-1
CONTROL
DAPI
TRITC
MERGED
OCT1
UPCI:SCC0084
UPCI:SCC0131
HPV-negative HNSCC cell lines
UDSCC2
93VU147T
HPV-positive HNSCC cell lines

Figure S1b

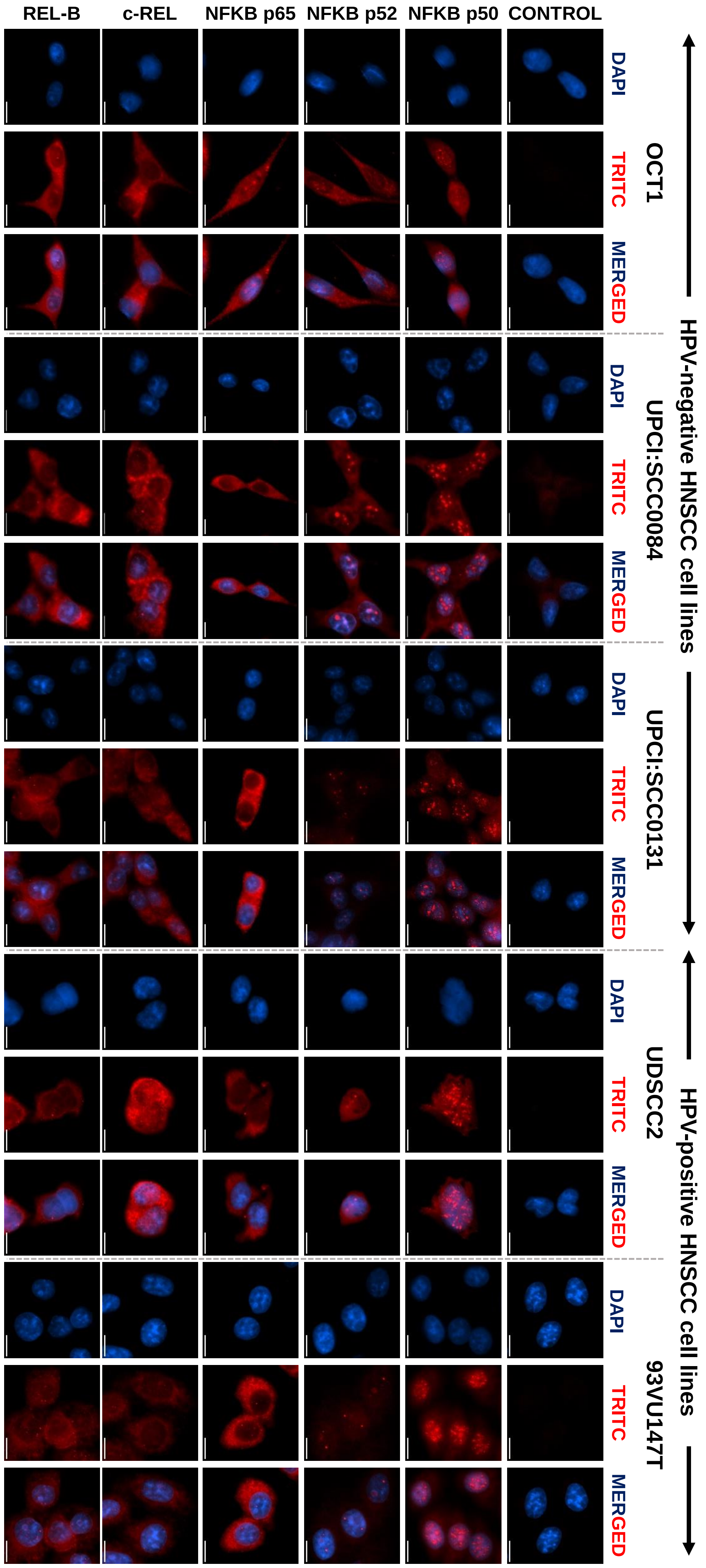
REL-B
c-REL
NFKB p65
NFKB p52
NFKB p50
CONTROL
DAPI
TRITC
MERGED
OCT1
UPCI:SCC0084
UPCI:SCC0131
HPV-negative HNSCC cell lines
UDSCC2
93VU147T
HPV-positive HNSCC cell lines

Figure S1c

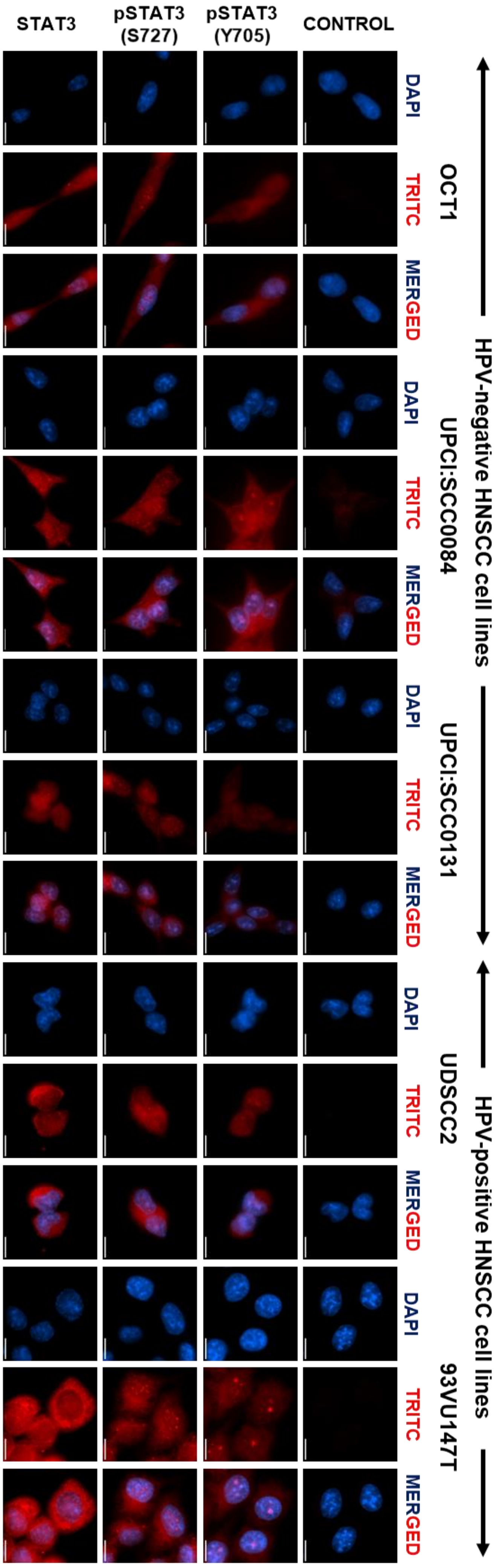
STAT3
pSTAT3 (S727)
pSTAT3 (Y705)
CONTROL
DAPI
TRITC
MERGED
OCT1
UPCI:SCC0084
UPCI:SCC0131
UDSCC2
93VU147T
HPV-negative HNSCC cell lines
HPV-positive HNSCC cell lines

Figure S1d

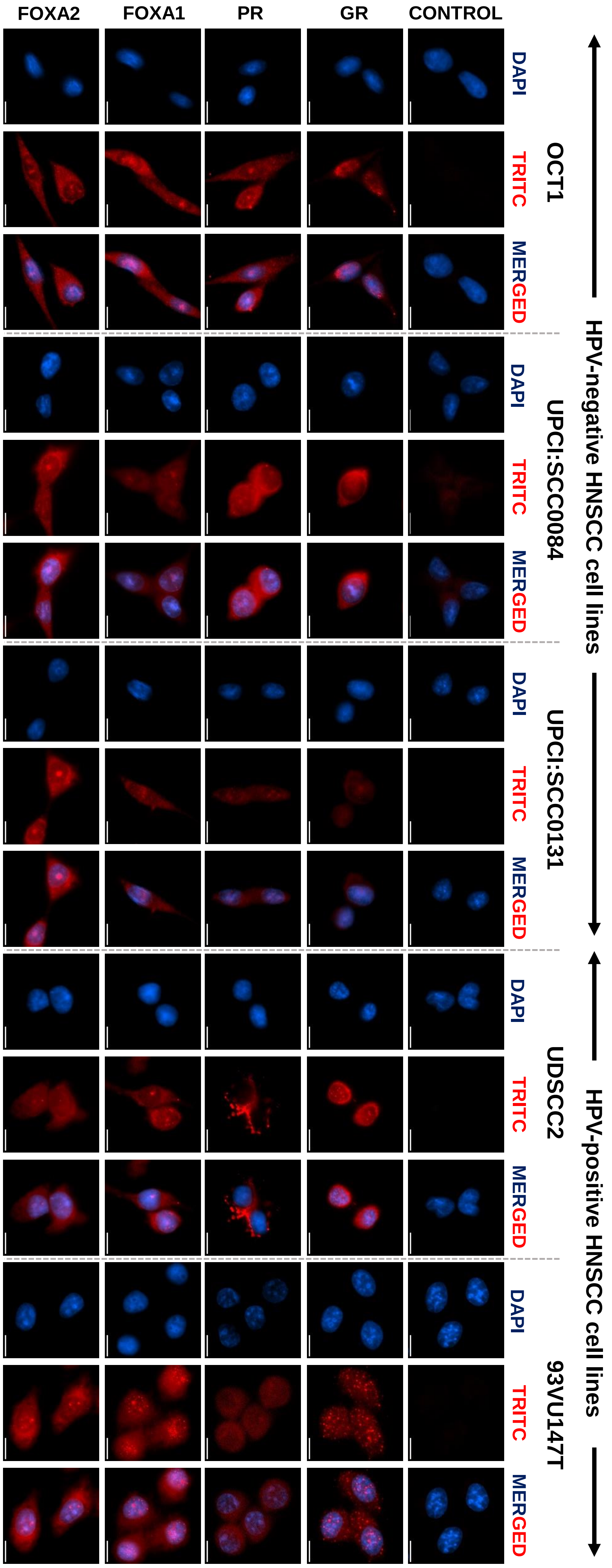
FOXA2
FOXA1
PR
GR
CONTROL
DAPI
TRITC
MERGED
OCT1
UPCI:SCC0084
UPCI:SCC0131
UDSCC2
93VU147T
HPV-negative HNSCC cell lines
HPV-positive HNSCC cell lines

Figure S1e

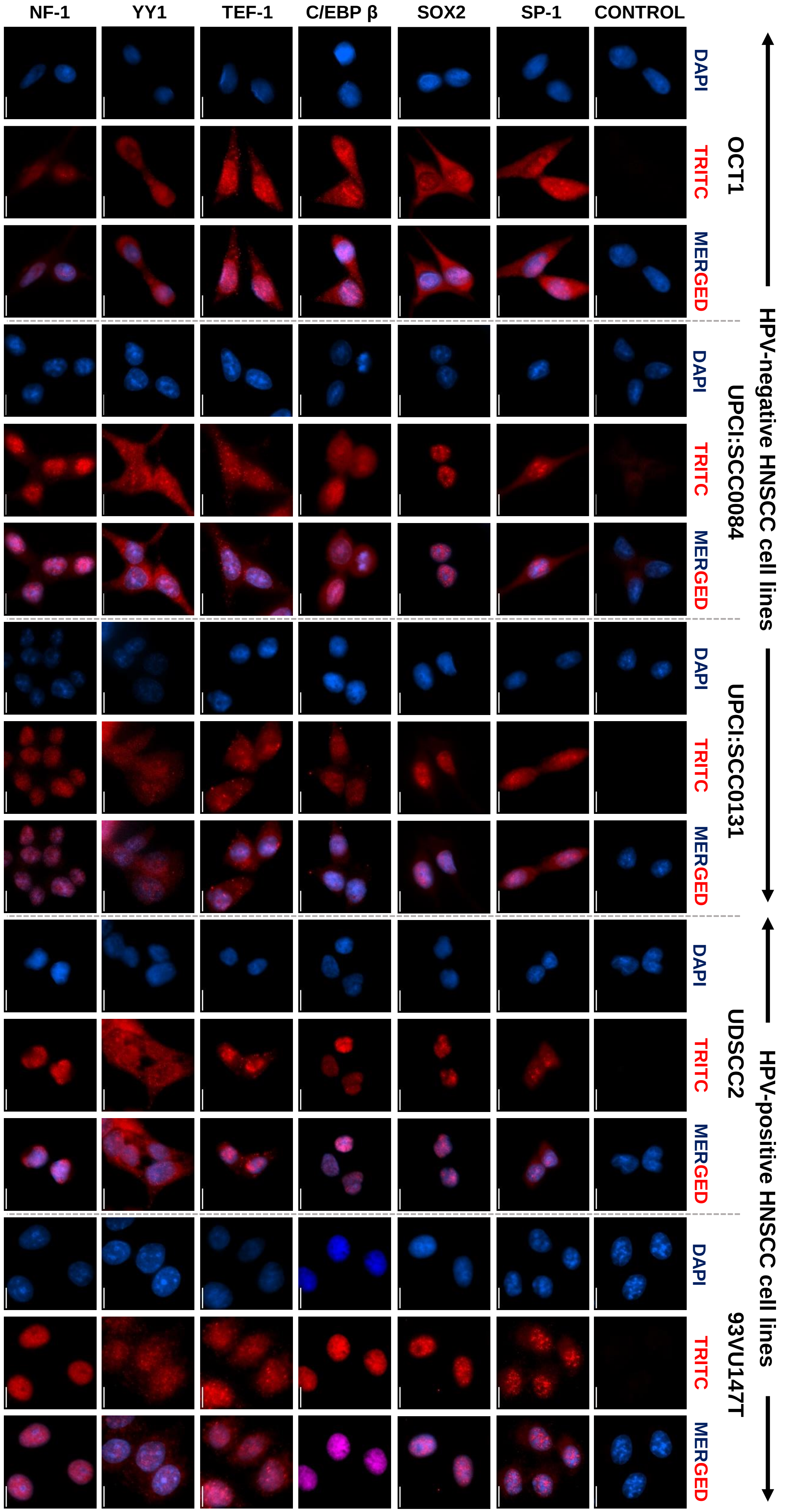

Figure S1: Representative fluorescence photomicrographs showing subcellular localization of LCR-associated transcription factors in HNSCC cell lines. (a) AP-1 family (b) NF-κB family (c) STAT3 and its phosphorylated forms (d) Other inducible transcription factors (e) Constitutive transcription factors *(High resolution images for Figure 3A)*

Figure S2a

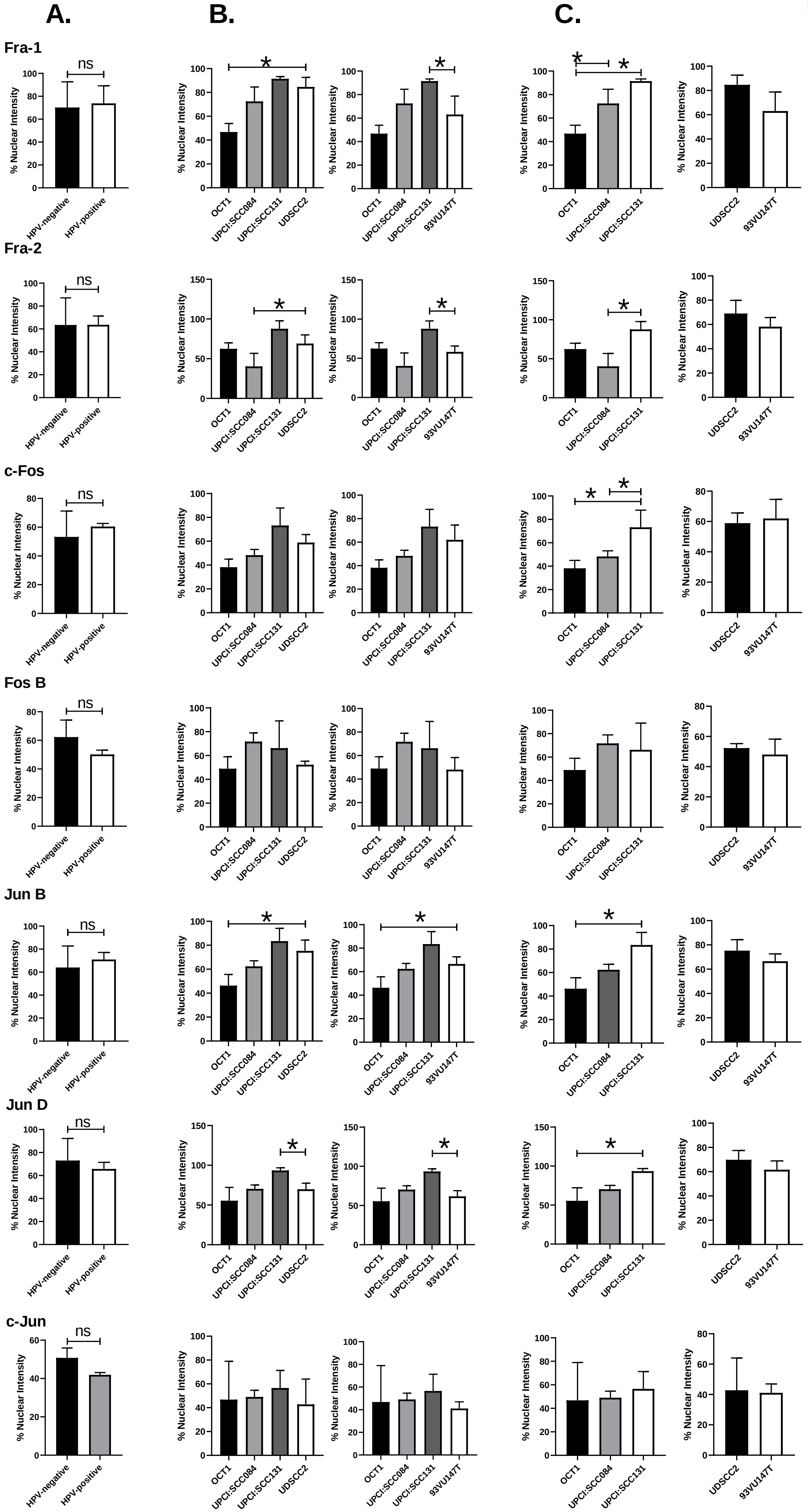
A.
B.
C.
Fra-1
Fra-2
c-Fos
Fos B
Jun B
Jun D
c-Jun
ns
*
% Nuclear Intensity
HPV-negative
HPV-positive
OCT1
UPCI:SCC084
UPCI:SCC131
UDSCC2
93VU147T

Figure S2b

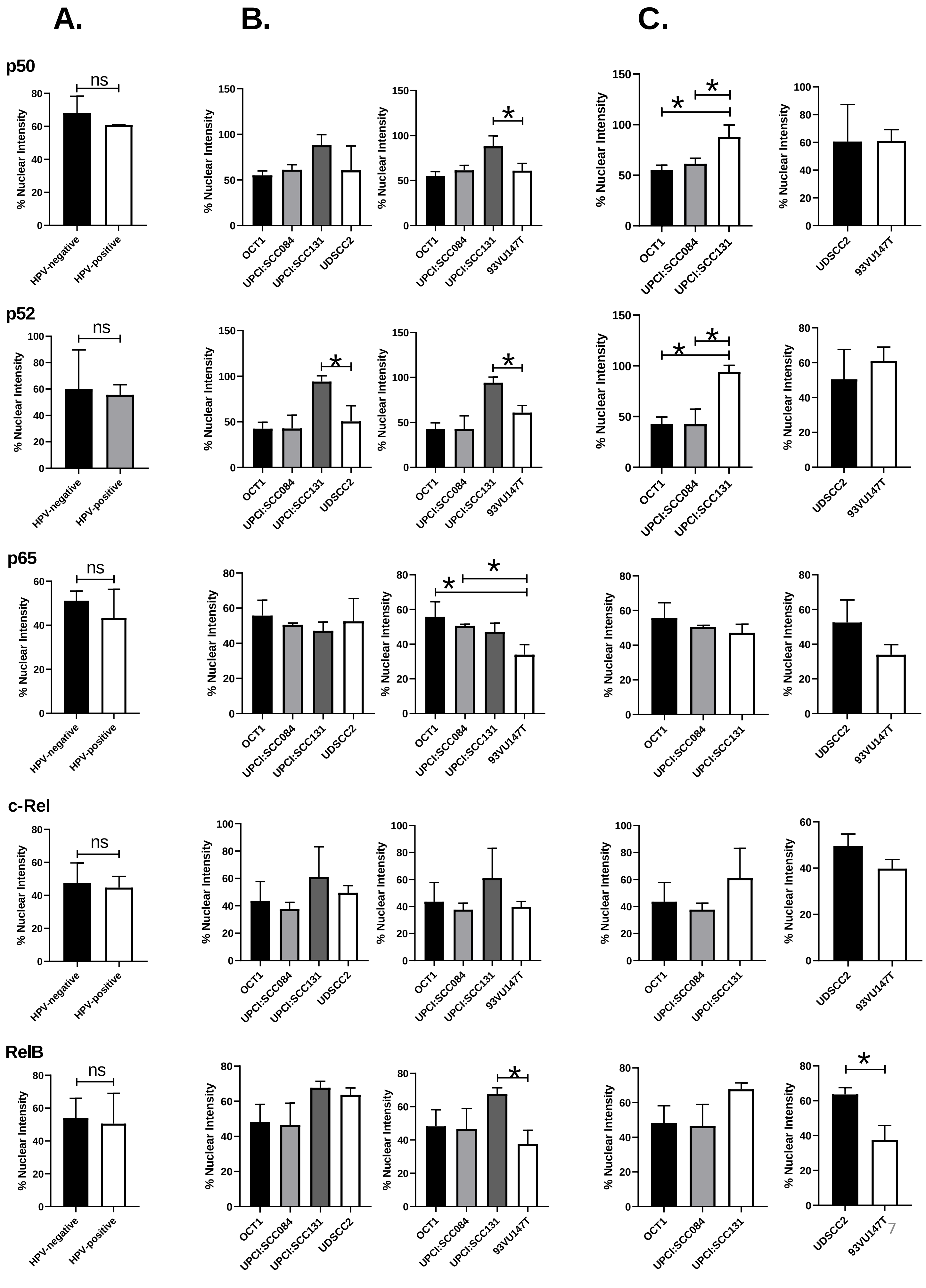
A.
B.
C.
p50
p52
p65
c-Rel
RelB
% Nuclear Intensity
ns
*
HPV-negative
HPV-positive
OCT1
UPCI:SCC084
UPCI:SCC131
UDSCC2
93VU147T

Figure S2c

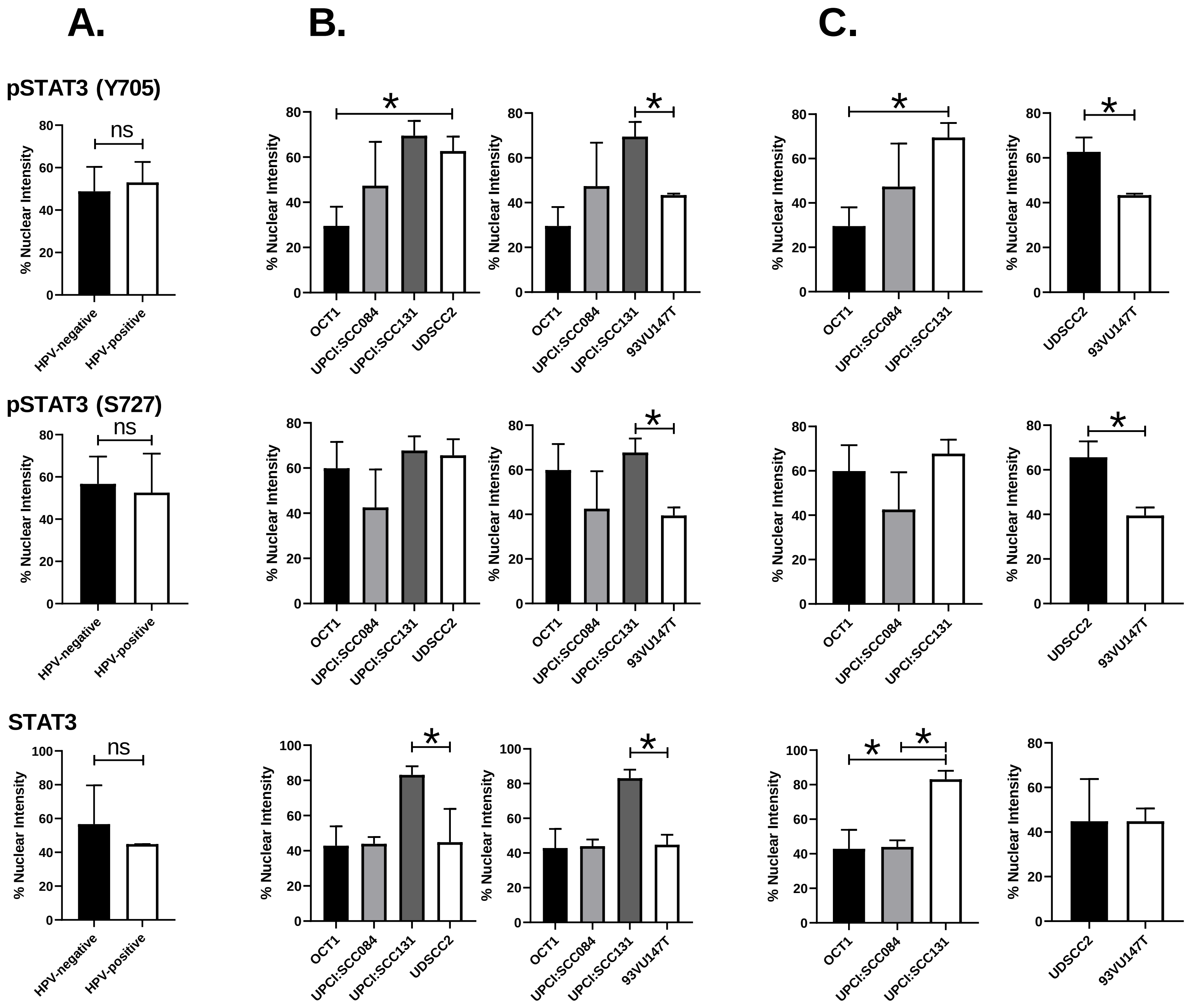
A.
B.
C.
pSTAT3 (Y705)
pSTAT3 (S727)
STAT3
% Nuclear Intensity
ns
*
HPV-negative
HPV-positive
OCT1
UPCI:SCC084
UPCI:SCC131
UDSCC2
93VU147T

Figure S2d

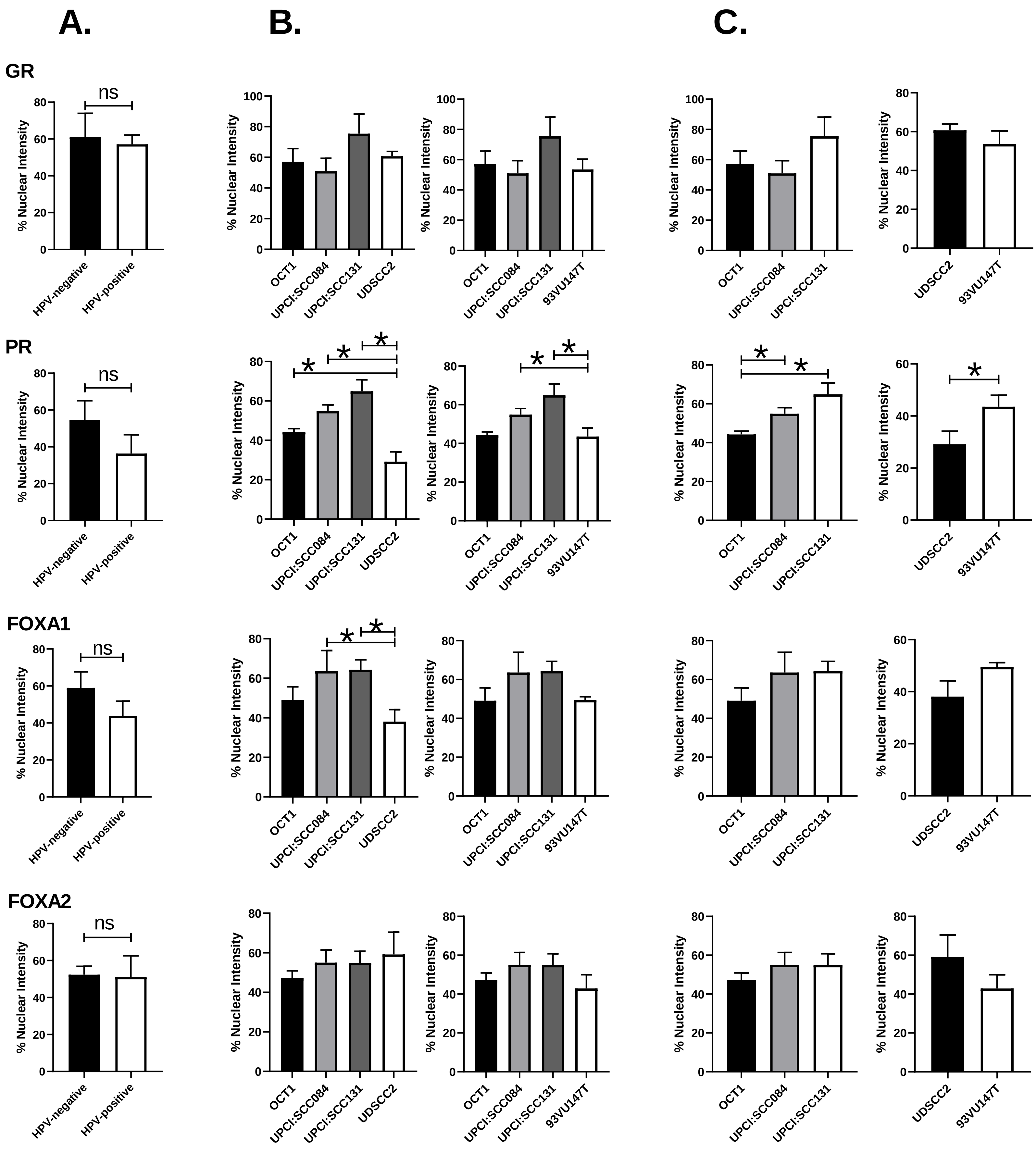
A.
B.
C.
GR
PR
FOXA1
FOXA2
ns
*
% Nuclear Intensity
HPV-negative
HPV-positive
OCT1
UPCI:SCC084
UPCI:SCC131
UDSCC2
93VU147T

Figure S2e

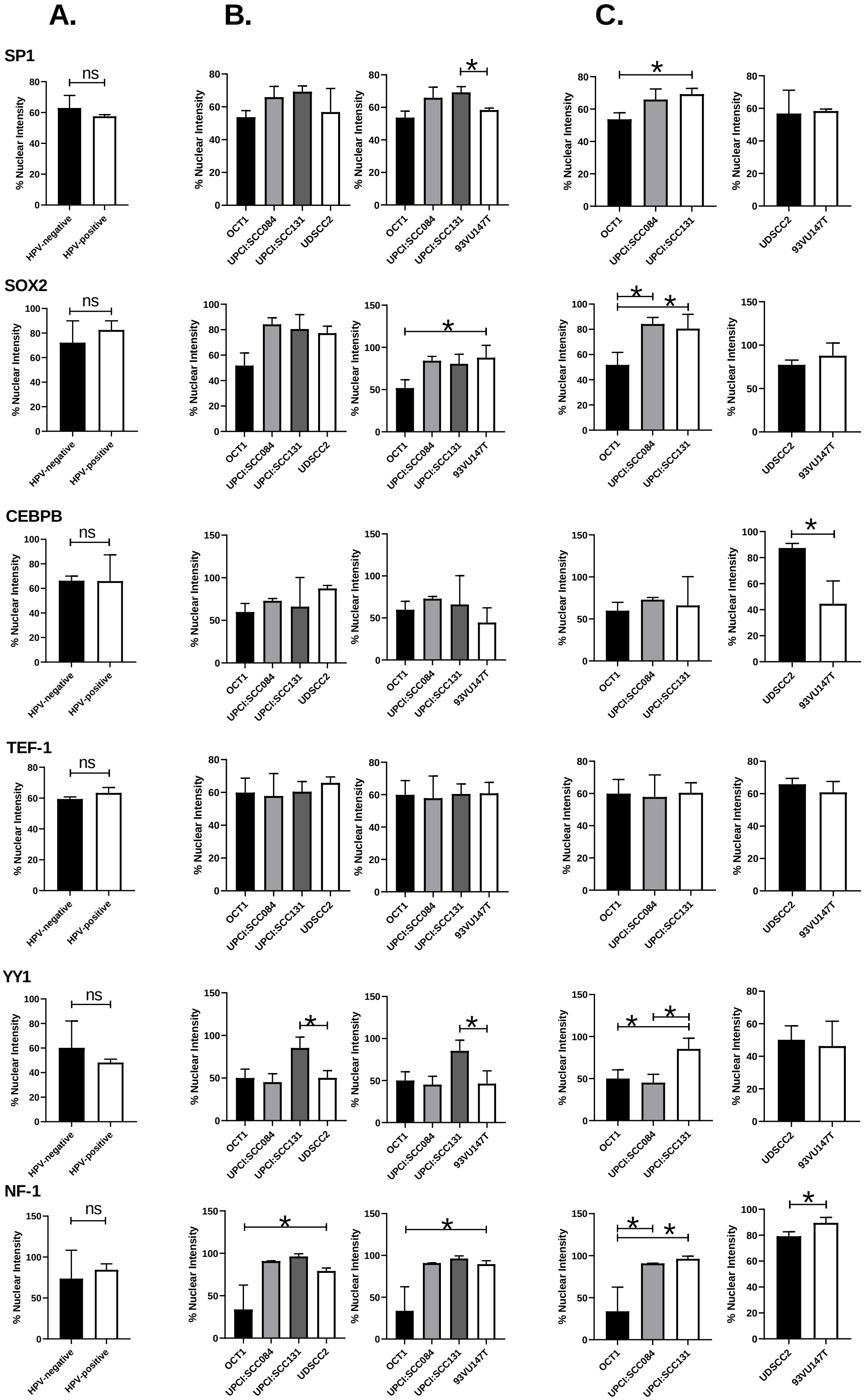

Figure S2:Quantitative estimation of nuclear positivity of LCR-associated transcription factors in HNSCC cell lines. a. AP-1 family **b.** NF-κB family **c.** STAT3 **d.** Other inducible transcription factors **e.** Constitutively-active transcription factors. **A.** The bar graph represents the aggregated normalized nuclear intensity of LCR-associated transcription factor family members that were grouped into HPV-positive and HPV-negative as per the HPV status of the cells. Data plotted as mean band intensity of LCR-associated transcription factor members ± SEM (n=3 for HPV-negative and n=2 for HPV-positive) of the representative experiment out of three independent experiments *p value <0.05. **B.** The bar graphs represent the percentage nuclear positivity of LCR-associated transcription factor family members in HPV-negative HNSCC cells w.r.t. individual HPV-positive HNSCC cells (left panel: UDSCC2 and right panel: 93VU147T). **C.** The bar graphs represent the percentage of nuclear positivity of LCR-associated transcription factor family members within HPV-negative HNSCC cells (left panel) and HPV-positive HNSCC cells (right panel). Data plotted as mean of percentage nuclear positivity ± SD of the representative experiment out of three independent experiments **p value <0.05*.

Figure S3

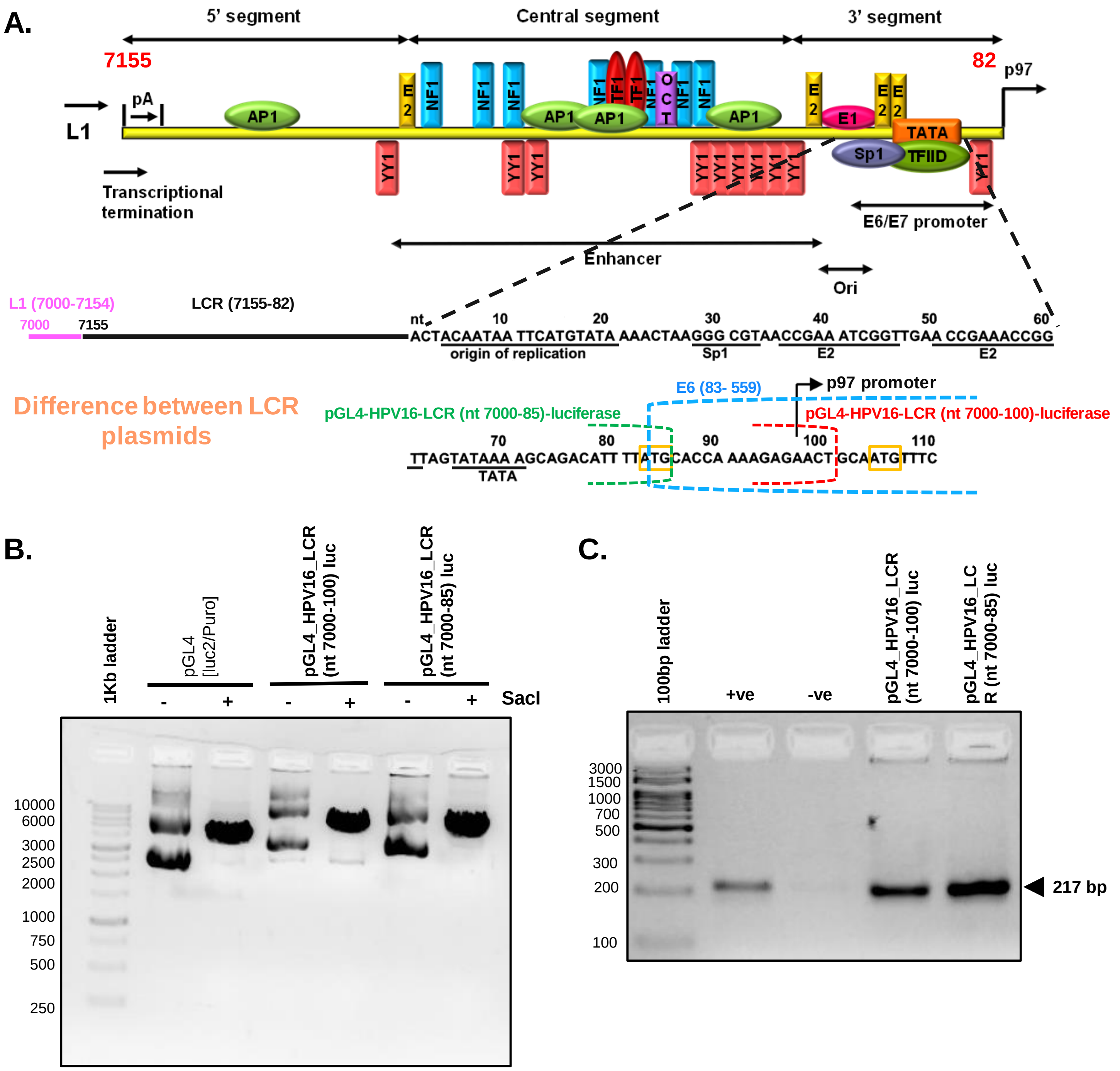

Figure S3: HPV16-LCR plasmids structural analysis, expansion and validation. A. Schematic representation of the key differences in the vector pGL4-LCR-HPV16 (nt 7000-100) and pGL4-LCR-HPV16 (nt 7000-85). **B.** Images of the LCR plasmids digested with *SacI* with undigested plasmids run on 1% agarose gel to check the expected size of the plasmid. **C.** Images of PCR products run on 2% agarose gel showing amplification of specific amplification of HPV16 LCR DNA in pGL4-LCR-HPV16 (nt 7000-100) and pGL4-LCR-HPV16 (nt 7000-85) plasmid. Positive control: International standard of HPV16 (10,000 copy number/μL). Nuclease-free water was used as a negative control. 100ng DNA was used for each PCR. Marker: 100bp ladder.

Figure S4

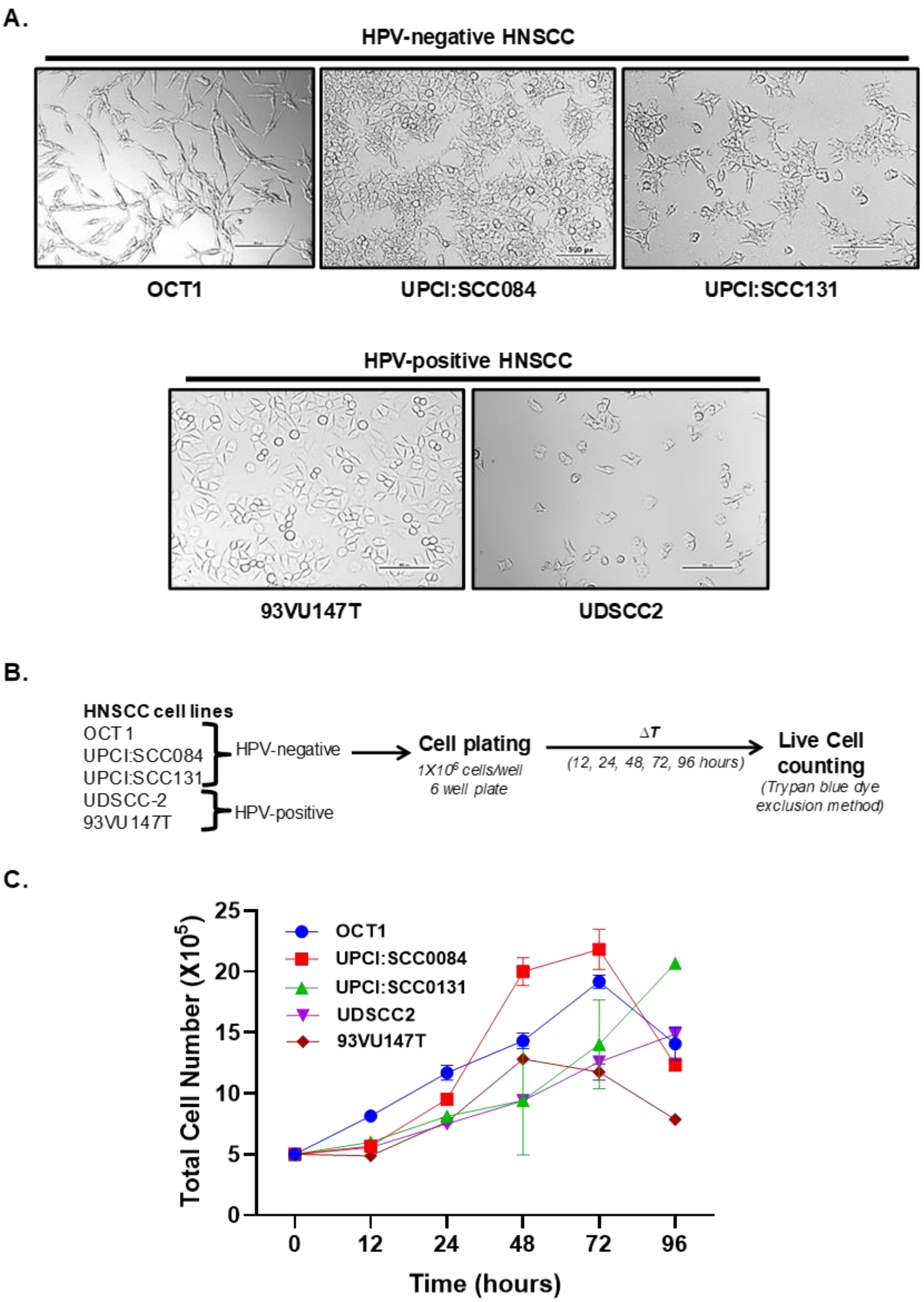

Figure S4: Cell culture characteristics of HNSCC cells used for present study. A. Photomicrographs of cultures of 93UV147T, UDSCC-2 (HPV16-positive) and OCT-1, UPCI:SCC084, UPCI:SCC131 (HPV-negative) captured by ECLIPSE Ti inverted microscope (Nikon) at original magnification, 100X. **B.** Schematic representation of the experiment to check cell growth in HNSCC cells. **C.** Cell growth of HNSCC cell lines. Graph showing the total live cell count of the cultures seeded with initial density of $5x10^5$ cells/well. Values are mean of total cell count ±SD of a representative experiment conducted in triplicate.

Figure S5

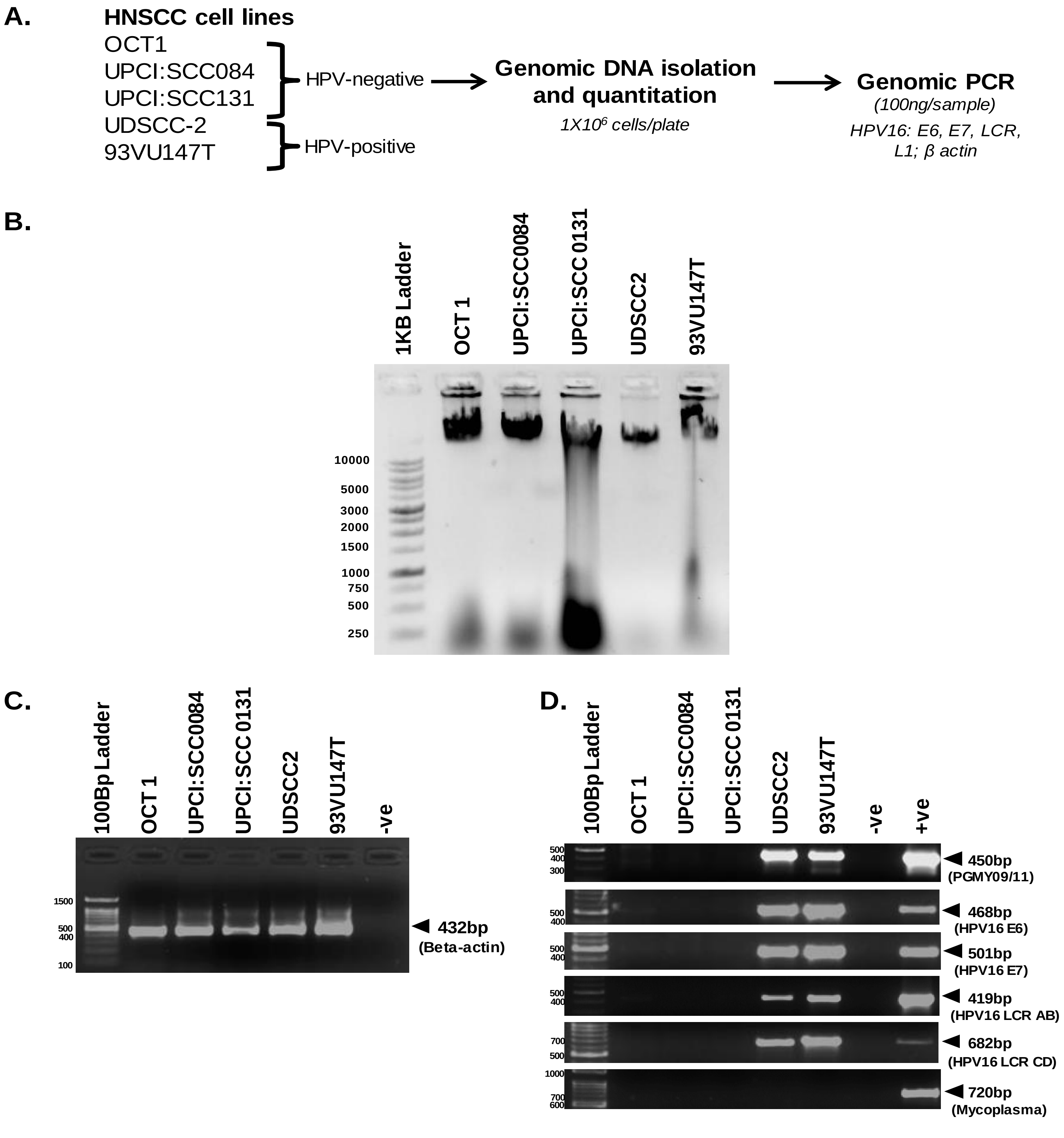

Figure S5: Confirmation of mycoplasma-free cultures and confirmation of HPV-status of HNSCC cell lines using HPV-type specific PCRs. A. Representative image of isolated genomic DNA (1µg/lane) of different HNSCC cells using QIAamp DNA mini kit and run on agarose gel (1%). Marker: 1 Kb ladder. **B.** Images of PCR products run on 2% agarose gel showing amplification of β-actin to check the integrity of genomic DNA. **C.** Images of PCR products run on 2% agarose gel showing amplification of specific amplification of HPV L1 (PGMY09/11), HPV16 E6, E7, LCR, and mycoplasma DNA in HNSCC cell lines. Positive control for HPV-type specific PCRs: International standard of HPV16 (10,000 copy number/µL) and for mycoplasma PCR: known mycoplasma positive cells' DNA. Nuclease free water was used as negative control. 100ng DNA was used for each PCR. Marker: 100bp ladder.

Figure S6

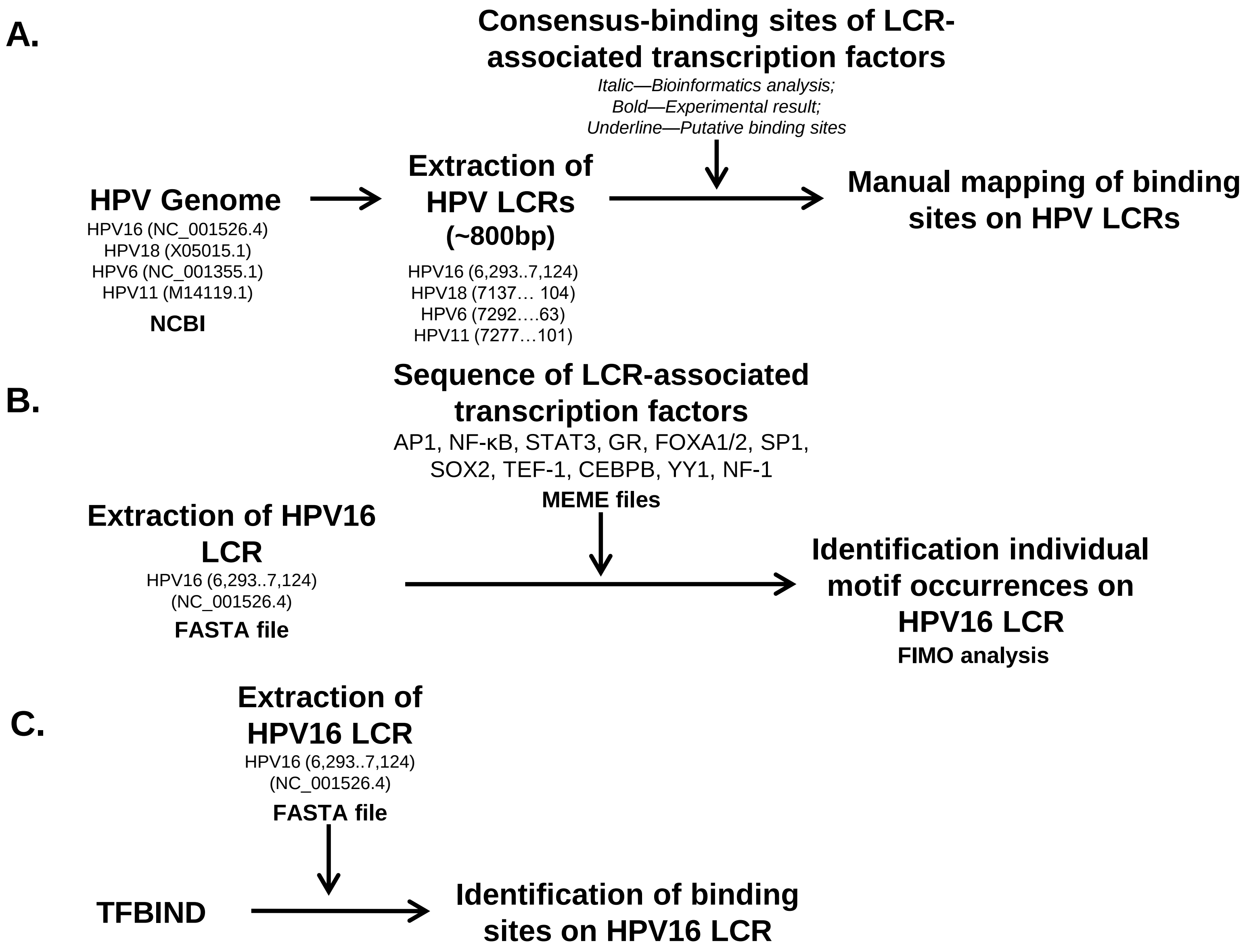

Figure S6: Identification of HPV LCR-associated transcription factor using bioinformatics tools. Schematic representation of the analysis steps followed for manual mapping of transcription factor consensus sites on HPV LCRs (A). Identification of binding sites present on HPV16 LCR using TFBIND (B) and individual motif occurrences of LCR-associated transcription factors on HPV16 LCR using FIMO analysis (C). LCR: Long Control Region.

Figure S7

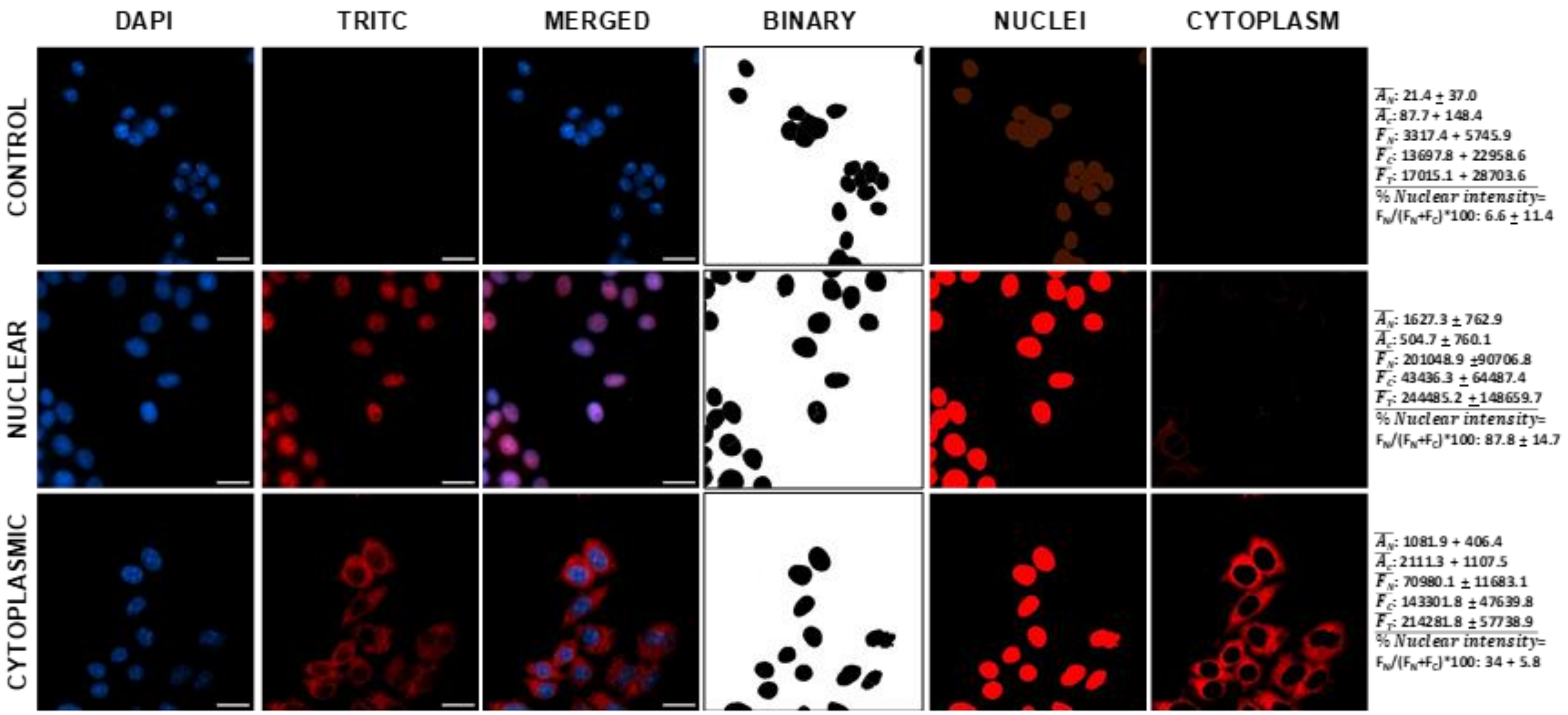

Figure S7: Representative images showing image processing for determination of nuclear localization of respective transcription factors. Representative images of control (upper panel), nuclear (middle panel), and cytoplasmic (lower panel) localization of transcription factor using ImageJ. Binary images were used for the generation of masks to mark the nuclear region of interest. The nuclear mask was overlayed over the cytoplasmic mask to determine the integrated density of the nucleus and cytoplasm, respectively. *(Scale: 20μm).*

Table S1: List of transcription factors motif occurrence on HPV16 LCR (NC_001526.4) using FIMO analysis.

Table S2: List of transcription factors motif occurrence on HPV16 LCR (NC_001526.4) using TFBIND

Table S3: List of different human HNSCC cell lines used in the present study.

Cell line	Tissue of origin	Key Characteristics	Cell Doubling Time (Cellosaurus) (hours)	Culture Media	Source	Ref.
**OCT1**	Lateral Tongue	**HPV-negative**<br>Morphology: epithelial<br>Growth property: adherent	NA	DMEM low glucose	INMAS, DRDO	**(Bhatt *et al.* 2015)**
**UPCI: SCC084**	Oral cavity, retromolar trigone	**HPV–negative**<br>Morphology: epithelial<br>Growth property: adherent<br>Male, 52-year-old	~22	DMEM F12	University of Pittsburgh	**(Telmer *et al.* 2003)**
**UPCI: SCC131**	Floor of mouth	**HPV-negative**<br>Morphology: epithelial<br>Growth property: adherent<br>Male, 73-year-old	~50-60	DMEM F12	University of Pittsburgh	**(White *et al.* 2007)**
**UDSCC2**	Hypophary nx	**HPV16-positive**<br>Morphology: epithelial<br>Growth property: adherent<br>Male, 58-year-old	42	MEM	University of Dusseldorf	**(Ballo *et al.* 1999)**
**93VU147T**	Floor of mouth	**HPV16-positive**<br>Morphology: epithelial<br>Growth property: adherent<br>Male, 58-year-old	52	DMEM high glucose	Vrije Universiteit	**(Steenbergen *et al.* 1995)**

Abbreviations: DMEM: Dulbecco's Modified Eagle's Medium; HPV: Human Papillomavirus; INMAS: Institute of Nuclear Medicine & Allied Sciences-DRDO; MEM: Minimum Essential Medium; NA: Not available

Table S4: List of primers used for genomic and reverse transcriptase PCR, and their key characteristics.

**Primer sets** (Lab ID)	**Target**	**PCR Type**	**Primer sequence**	**Amplicon size (bp)**	**Annealing temp. (°C)**
**β –actin** (P1019A P1019B)	Human β - actin	Genomic	F 5'-CGGCCAGCCAGGTCCAGACG-3'<br>R 5'-CCAGGCACCAGGGCGTGATG-3'	436	55
**Mycoplasma** (P1014A, P1014B)	Mycoplasma	Genomic	F 5'-ACTCCTACGGGAGGCAGCAGTA-3'<br>R 5'-TGCACCATCTGTCACTCTGTTAACCTC-3'	720	55
**HPV16 E6** (P1008A P1008B)	E6	Genomic	F 5'-GAAACCGGTTAGTATAAAAGCAGAC– 3'<br>R 5'-AGCTGGGTTTCTCTACGTGTTCT– 3'	468	55
**HPV16 E7** (P1009A P1009B)	E7	Genomic	F 5'-CCATAATATAAGGGGTCGGTGGA-3'<br>R 5'-TTTTTCACTACAGCCTCTACAT-3'	501	55
**PGMY09/11** (P1001A-R)	L1	Genomic	F: PGMY09-F: 5'-CGTCCCAAAGGAAACTGATC-3'<br>PGMY09-G: 5'-CGACCTAAAGGAAACTGATC-3'<br>PGMY09-H: 5'-CGTCCAAAAGGAAACTGATC-3'<br>PGMY09-I: 5'-GCCAAGGGGAAACTGATC-3'<br>PGMY09-J: 5'-CGTCCCAAAGGATACTGATC-3'<br>PGMY09-K: 5'-CGTCCAAGGGGATACTGATC-3'<br>PGMY09-L: 5'-CGACCTAAAGGGAATTGATC-3'<br>PGMY09-M: 5'-CGACCTAGTGGAAATTGATC-3'<br>PGMY09-N: 5'-CGACCAAGGGGATATTGATC-3'<br>PGMY09-P: 5'-GCCCAACGGAAACTGATC-3'<br>PGMY09-Q: 5'-CGACCCAAGGGAAACTGGTC-3'<br>PGMY09-R: 5'-CGTCCTAAAGGAAACTGGTC-3'<br>R: PGMY11-A: 5'-GCACAGGGACATAACAATGG-3'<br>PGMY11-B: 5'-GCGCAGGGCCACAATAATGG-3'<br>PGMY11-C: 5'-GCACAGGGACATAATAATGG-3'<br>PGMY11-D: 5'-GCCCAGGGCCACAACAATGG-3'<br>PGMY11-E: 5'-GCTCAGGGTTTAAACAATGG-3'	450	55
**LCR AB** (P1259A P1259B)	LCR	Genomic	F 5'-CCTCATCTACCTCTACAACTGCTAAACGG-3'<br>R 5'-GTTTAAACCATAGTTGCTGACATAGAAC-3'	419	56
**LCR CD** (P1259C P1259D)	LCR	Genomic	F 5'-GCTTCAACCGAATTCGGTTGCATG-3'<br>R 5'-CGTCGCAGTAACTGTTGCTTGCAGTACACAC-3'	682	56
**RT-HPV16 E6** (P1290A P1290B)	HPV16 E6	Transcript	F 5'-AATGTTTCAGGACCCTACGG-3'<br>R 5'-TCAGGACACAGTGGCTTTTG-3'	157	55
**RT-HPV16 E7** (P1291 A P1291 B)	HPV16 E7	Transcript	F 5'-TTTGCAACCAGAGACAACTGA-3'<br>R 5'-GCCCATTAACAGGTCTTCCA-3'	214	55
**RT-GAPDH** (P1284 A P1284 B)	Human GAPDH	Transcript	F 5'-GTCTCCTCTGACTTCAACAGCG-3'<br>R5'-ACCACCCTGTTGCTGTAGCCAA-3'	131	60

Abbreviations: bp: base pair; β-actin: Beta-Actin; E6/E7: Early protein 6/7; GAPDH: Glyceraldehyde 3-phosphate dehydrogenase; HPV: Human Papillomavirus; LCR: Long Control Region.

Table S5: List of antibodies used for immunoblotting and immunocytochemistry experiments.

Antibodies (Lab ID)	Source (Cat. No.)	Origin and type	Dilution*	Dilution#
**HPV 16/18 E6** (#397)	Santa Cruz (SC-460)	Mouse monoclonal IgG	1:5000	1:100
**HPV 16 E7** (#356)	Abcam (ab-20191)	Mouse monoclonal IgG	1:5000	1:100
**Fra-1** (#40)	Santa Cruz (SC-605)	Rabbit polyclonal IgG	1:5000	1:100
**Fra-2** (#51)	Santa Cruz (SC-171)	Rabbit polyclonal IgG	1:5000	1:100
**c-Fos** (#49)	Santa Cruz (SC-7202)	Rabbit polyclonal IgG	1:5000	1:100
**Fos-B** (#54)	Santa Cruz (SC-48)	Rabbit polyclonal IgG	1:5000	1:100
**JunB** (#55)	Santa Cruz (SC-73)	Rabbit polyclonal IgG	1:5000	1:100
**JunD** (#48)	Santa Cruz (SC-74)	Rabbit polyclonal IgG	1:5000	1:100
**c-Jun** (#288)	Santa Cruz (SC-45)	Rabbit polyclonal IgG	1:5000	1:100
**NF-κB p50**(#18)	Santa Cruz (SC-7178)	Rabbit polyclonal IgG	1:5000	1:100
**NF-κB p52** (#22)	Santa Cruz (SC-298)	Rabbit polyclonal IgG	1:5000	1:100
**NF-κB p65** (#404)	Santa Cruz (SC-8008)	Mouse monoclonal IgG	1:5000	1:100
**c-Rel** (#28)	Santa Cruz (SC-70)	Rabbit polyclonal IgG	1:5000	1:100
**RelB** (#26)	Santa Cruz (SC-226)	Rabbit polyclonal IgG	1:5000	1:100
**pSTAT3 (Y705)** (#132)	BD Bio. (612543)	Mouse monoclonal IgG	1:5000	1:100
**pSTAT3 (s727)** (#211)	BD Bio. (612357)	Mouse Monoclonal IgG	1:5000	1:100
**STAT3** (#134)	BD Bio.(610190)	Mouse monoclonal IgG	1:5000	1:100
**GR** (#407)	Santa Cruz (SC-393232)	Mouse monoclonal IgG	1:5000	1:100
**PR** (#408)	Santa Cruz (SC-166169)	Mouse monoclonal IgG	1:5000	1:100
**FOXA1** (#409)	Santa Cruz (SC-101058)	Mouse monoclonal IgG	1:5000	1:100
**FOXA2** (#402)	Santa Cruz (SC-374376)	Mouse monoclonal IgG	1:5000	1:100
**SP1** (#405)	Santa Cruz (SC-17824)	Mouse monoclonal IgG	1:5000	1:100
**SOX2** (#406)	Santa Cruz (SC-365823)	Mouse monoclonal IgG	1:5000	1:100
**CEBPB** (#401)	Santa Cruz (SC-7962)	Mouse monoclonal IgG	1:5000	1:100
**TEF-1** (#403)	Santa Cruz (SC-393976)	Mouse monoclonal IgG	1:5000	1:100
**YY1** (#78)	Santa Cruz (SC-1703)	Rabbit polyclonal IgG	1:5000	1:100
**NF-1** (#86)	Santa Cruz (SC-5567)	Rabbit polyclonal IgG	1:5000	1:100
**Beta Actin** (#383)	Santa Cruz (SC-47778)	Mouse monoclonal IgG	1:10000	-
**Anti-mouse-HRP** (#395)	Santa Cruz (SC-2031)	Goat anti-mouse IgG	1:5000	-
**Anti- Rabbit-HRP** (#362)	Santa Cruz (SC-2030)	Goat anti-rabbit IgG	1:5000	-
**Anti-mouse-AlexaFluor 594** (#268)	Invitrogen (A11005)	Goat polyclonal IgG	-	1:250
**Anti-rabbit-AlexaFluor 594** (#269)	Invitrogen (A11037)	Goat polyclonal IgG	-	1:250

***Dilution in Immunoblotting #Dilution in Immunocytochemistry **Abbreviation:** CEBPB: CCAAT enhancer binding protein beta; c-Fos: proto-oncogene c-FBJ murine osteosarcoma; E6/E7: Early protein 6/7; Fos-B: FBJ murine osteosarcoma viral oncogene homolog B; FOXA: Forkhead box A; Fra-1: Fos-related antigen 1; GR: Glucocorticoid receptor; HPV: Human Papillomavirus; HRP: Horseradish peroxidase; NF-1: Nuclear Factor 1; NF-κB: Nuclear factor kappa B; PR: Progesterone receptor; pSTAT3(Y701): phospho- Signal Transducer And Activator Of Transcription 3; RelB: V-rel reticuloendotheliosis viral oncogene homolog B; SOX2: SRY-related HMG-box 2; SP1: Specificity protein 1; STAT3: Signal Transducer And Activator Of Transcription 3; TEF-1: Translation elongation factor-1; YY1: Yin Yang 1.

Table S6: List of different plasmids used in the present study.

**Plasmids** (Lab ID)	**Source**	**Cat No.**	**Vector Backbone**	**Size**	**Cloning Sites**	**Bacterial Resistance**	**Growth Strain (Temperature)**
**pGL4-HPV16-LCR (nt 7000-85)-Luciferase** (V0020)	Addgene	32888	pGL4	*~5kb*	*SacI BglII*	Ampicillin, 100µg/mL	DH5alpha (37ºC)
**pGL4-HPV16-LCR (nt 7000-100)-Luciferase** (V0021)	Addgene	32889	pGL4	*~5kb*	*SacI BglII*	Ampicillin, 100µg/mL	DH5alpha (37ºC)
**pRL-TK Vector** (V0025)	Promega	E2241	pRL	4045	*BamHI*	Ampicillin, 100µg/mL	DH5alpha (37ºC)
**pGL4.20[luc2/Puro]** (V0024)	Promega	E6751	pGL4	4242	*SacI BglII*	Ampicillin, 100µg/mL	DH5alpha (37ºC)

Abbreviation: DH5alpha: Douglas Hanahan 5 alpha; HPV: Human Papillomavirus; LCR: Long Control Region.

Table S7: List of the reagents used (with their company and catalog number).

Material	Company	Catalog no.
Dulbecco's Modified Eagle Medium (DMEM), High glucose, L-Glutamine, Sodium bicarbonate and Sodium pyruvate	HiMEDIA	AL007A
Dulbecco's Modified Eagle Medium (DMEM), Low glucose, L-Glutamine, Sodium bicarbonate and Sodium pyruvate	HiMEDIA	AL006
Minimum Essential Medium (MEM), w/ Earle's salts, L-Glutamine, Sodium pyruvate, NEAA and Sodium bicarbonate	HiMEDIA	AL047S
Dulbecco's Modified Eagle Medium / Nutrient Mixture F-12 Ham (DMEM / F12, 1:1 mixture) L-Glutamine, HEPES buffer, Sodium pyruvate and Sodium bicarbonate	HiMEDIA	AL140S
Gibco™ Fetal Bovine Serum, qualified, Brazil	Thermo Scientific™	11573397
Antibiotic Antimycotic Solution 100X Liquid, Endotoxin tested	HiMEDIA	A002A
Trypsin Phosphate Versene Glucose (TPVG)	HiMEDIA	TCL022
QIAamp DNA mini kit	QIAGEN	56304
PCR Master Mix	Thermo Scientific™	K0171
100bp DNA Ladder RTU	GeneDirex	DM001-R500
1Kb DNA Ladder RTU	GeneDirex	DM010-R500
RNeasy Mini Kit	QIAGEN	74104
Verso cDNA Synthesis Kit	Thermo Scientific™	AB1453A
Precision Plus Protein Dual Color Standards	Bio-Rad	1610374
Luminol detection kit	Santa Cruz Biotechnology	sc-2048
Fluoroshield™ with DAPI	Merck	F6057
EndoFree Plasmid Maxi Kit	Qiagen	12362
SacI-HF	New England Biolabs	R3156
BamHI-HF	New England Biolabs	R3136
Lipofectamine™ 2000 Transfection Reagent	Invitrogen™	11668019
Dual-luciferase reporter assay system	Promega	E1910

Abbreviations: cDNA: copy DNA; DAPI: (2-(4-Amidinophenyl)-6-indolecarbamidine dihydrochloride); DMEM: Dulbecco's Modified Eagle Medium; DNA: Deoxyribonucleic Acid; HEPES: (4-(2-hydroxyethyl)-1-piperazineethanesulfonic acid); MEM: Minimum Essential Medium Eagle; NEAA: Non-Essential Amino Acid; PCR: Polymerase Chain Reaction; RPMI: Roswell Park Memorial Institute; RTU: Ready to use; TPVG: Trypsin Phosphate Versene Glucose.
